# Untargeted/Targeted 2D Gas Chromatography/Mass Spectrometry Detection of the Total Volatile Tea Metabolome

**DOI:** 10.3390/molecules24203757

**Published:** 2019-10-18

**Authors:** Joshua Morimoto, Marta Cialiè Rosso, Nicole Kfoury, Carlo Bicchi, Chiara Cordero, Albert Robbat

**Affiliations:** 1 Department of Chemistry, Tufts University, Medford, MA 02155, USA; joshua.morimoto@tufts.edu (J.M.); nicole.kfoury@tufts.edu (N.K.); 2 Dipartimento di Scienza e Tecnologia del Farmaco, Università degli Studi di Torino, 10125 Turin, Italy; marta.cialierosso@unito.it (M.C.R.); carlo.bicchi@unito.it (C.B.); chiara.cordero@unito.it (C.C.)

**Keywords:** tea, metabolomics, GC/MS, software, database, MS subtraction, spectral deconvolution, 2DGC, volatilomics

## Abstract

Identifying all analytes in a natural product is a daunting challenge, even if fractionated by volatility. In this study, comprehensive two-dimensional gas chromatography/mass spectrometry (GC×GC-MS) was used to investigate relative distribution of volatiles in green, pu-erh tea from leaves collected at two different elevations (1162 m and 1651 m). A total of 317 high and 280 low elevation compounds were detected, many of them known to have sensory and health beneficial properties. The samples were evaluated by two different software. The first, GC Image, used feature-based detection algorithms to identify spectral patterns and peak-regions, leading to tentative identification of 107 compounds. The software produced a composite map illustrating differences in the samples. The second, Ion Analytics, employed spectral deconvolution algorithms to detect target compounds, then subtracted their spectra from the total ion current chromatogram to reveal untargeted compounds. Compound identities were more easily assigned, since chromatogram complexities were reduced. Of the 317 compounds, for example, 34% were positively identified and 42% were tentatively identified, leaving 24% as unknowns. This study demonstrated the targeted/untargeted approach taken simplifies the analysis time for large data sets, leading to a better understanding of the chemistry behind biological phenomena.

## 1. Introduction

Obtaining the total metabolome of a natural or biological sample is a significant challenge. Even when analyzed by both gas and liquid chromatography (GC, LC), analyzing complex mixtures results in the detection of hundreds, if not thousands, of compounds. For example, we found that climate-induced tea plant (*Camellia sinensis* (L.) Kuntze) interactions cause significant differences in both the presence and concentration of secondary metabolites [1,2,3]. Abiotic pressures alone influence the relative distribution of more than 750 volatile compounds, with ~450 detected in any given sample. Differences in elevation, seasonal temperature, and rainfall from 2013 to 2016 caused two-thirds of the metabolites to either increase or decrease in concentration, one-third of them by more than 100%. We also measured nonvolatile concentration differences, including the catechins (flavan-3-ol derivatives with high antioxidant power). Only five days after the onset of the East Asian monsoon rains began, catechin concentrations decreased while total polyphenols and antioxidant potential increased. In these studies, we consider the tea “environmental,” since we microwaved the leaves in the field to stop enzymatic oxidation; they were not processed to produce commercial tea.

We previously relied on automated sequential, 2D gas chromatography/mass spectrometry (GC-GC/MS) to build the environmental tea database and on GC/MS to quantify the analyte distribution in the samples. In this study, we used comprehensive 2D GC/MS (GC×GC/MS) to accomplish both tasks, building from the existing database. When comparing each technique, library-building by GC-GC/MS relied upon fifty 1-min sample transfers (heart-cuts) from the first (polar) to the second (nonpolar) column to obtain clean spectra. The total time to obtain 50 data files was 4.5 days. In contrast, GC×GC/MS, produced one data file in one hour. Although runtimes significantly differed, data analysis of the respective files for library-building purposes took about the same amount of time.

The terms targeted, untargeted, and feature detection are often applied to GC/MS and LC/MS data. In targeted analysis, a predetermined list of compounds is selected for the analysis, whereas untargeted involves the evaluation of all detectable compounds in the sample [4,5]. The following criteria affirm compound identity [6,7,8]. Positive identification requires confirmation by at least two independent measurements, such as sample and reference compound mass spectra and linear retention indices (LRI). Tentative identification is based on an acceptable match between sample and commercial library, database, or literature spectra. Because natural products and biological metabolomes contain hundreds to thousands of compounds [9], it is impractical to purchase reference standards to confirm all sample identities. Therefore, assignments are typically tentative for most compounds.

Given the numerous and diverse compounds in natural products and in metabolomic datasets, feature-based peak detection algorithms have become popular. *m/z* peak-retention time pairs are used to assess peak region commonalities and differences in large data sets to reduce data complexity. Because feature-based peak detection is overly sensitive to algorithmic parameters, coelution and instrument noise, it produces many more features than actual compounds [10,11,12], with less than 2% yielding identifiable analytes [13]. Although peak detection tools can differentiate samples [11,14,15], identifying why they differ is difficult, since features themselves provide no biological context [16,17]. Despite these drawbacks, feature-based detection software is popular among the metabolomics community [15].

In this study, we used GC×GC/MS to reveal compositional differences in farmer-processed green, pu-erh teas (herein called pu-erh), collected from the same farm, plants, elevations, and time-period as environmental tea. 2D data were analyzed by GC Image and Ion Analytics. The first was adopted to detect 2D-peaks and to delineate peak-regions covering the full chromatographic space. Similarities and differences between high and low elevation samples were highlighted by both peak-region feature distribution and visual feature rendering [18]. Additionally, we identified compounds where possible. The second provided an untargeted/targeted workflow, based on spectral deconvolution and spectral subtraction of analytes found in the sample, to produce clean mass spectra and quantifiable constituent differences. New algorithms facilitated the annotation process.

## 2. Results

### 2.1. Molecular Feature Detection

**Figure 1:**
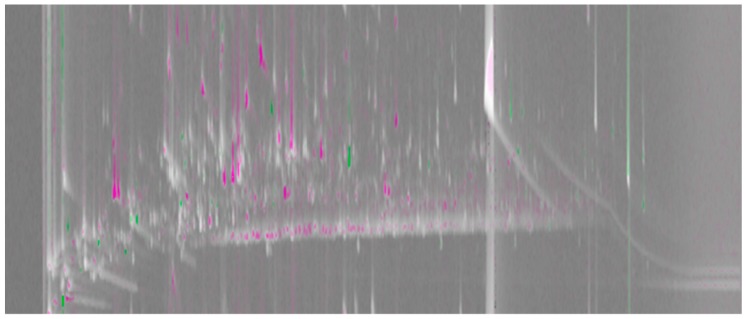
A comparative visualization between composite GC×GC/MS chromatograms of the high (reference image) and low (analyzed image) elevation teas. The magenta and green peak regions show the areas in the chromatogram where relative distribution of common analytes is higher in the high and low elevation teas, respectively. Light grey peak regions correspond to analytes with similar percentage response in the two chromatograms.

Figure 1 shows a comparative visualization, based on a visual features approach [18], highlighting compositional similarities and differences in the high (reference sample) and low (analyzed sample) elevation pu-erh teas. This pair-wise comparison is done on a composite chromatogram obtained by summing each sample’s 2D chromatograms (*n* = 2) after transformation and re-alignment. In total, 1450 peak-regions were delineated; 107 with spectra clean enough to make tentative compound assignments, see Supplementary Table S1. Each pixel in Figure 1 corresponds to detector acquisition points (scans) that show response differences. The algorithm subtracts the pixel value registered in the reference image from the corresponding value in the analyzed image, with the difference divided by the larger of the pixel values. The brighter the pixel, the larger the difference is relative to the analyzed pixel value; the darker the pixel, the larger the difference is relative to the reference pixel value. Medium gray indicates the difference is small relative to the value. The hue of a pixel indicates whether the analyzed image (green) or reference image (magenta) has the higher value. The saturation (color vs. grey) of a pixel indicates the magnitude of the difference ratio between the analyzed and reference images, with grey indicating equal pixel values and bold colors large differences.

### 2.2. Untargeted/Targeted Analysis

**Figure 2:**
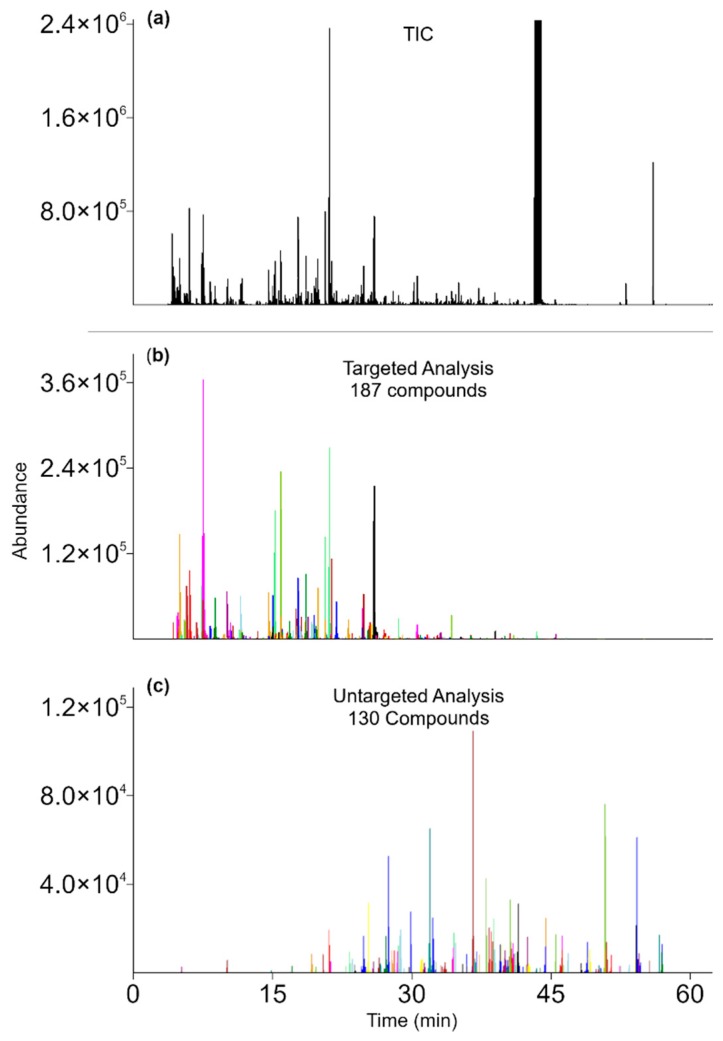
The TIC (**a**) and RIC chromatograms of targeted (**b**, in environmental tea) and untargeted (**c**, due to farmer processing) compounds in high elevation pu-erh tea.

Shown in Figure 2 are the (a) total and (b and c) reconstructed ion current (TIC and RIC) chromatograms for high elevation tea. Using the environmental database as target compounds, spectral deconvolution produced chromatogram (b), which reveals the 187 compounds in pu-erh tea that survived farmer processing. After subtracting the target compound spectra from the TIC chromatogram, untargeted analysis revealed another 130 compounds in the sample (c). Table 1 lists the breakdown of the number of compounds by their identification levels, showing 107, 132, and 78 positively and tentatively identified compounds, and unknowns in the high elevation pu-erh tea, respectively. Although 25% of the volatile constituents are unknowns, should statistical analysis reveal their importance, we know where in the separation to collect the compound by GC-GC for further analysis. In addition to retention and spectral information, the database includes sample type, the sensory characteristics of the compound in the sample, and known health benefits.

Compared to the 450 compounds we typically detect by stir bar sorptive extraction [19], head space solid phase microextraction (HS-SPME) produced a lower extraction yield, as expected, with 317 and 280 compounds in the high and low elevation samples, see Table 1. The high elevation sample contained 42 unique compounds; whereas only five were found in the low elevation teas. Also listed in the table are the number of targeted and untargeted compounds, see Supplementary Table S2 for identities. Once the total, detectable profile was established, GC Image (by pattern recognition) confirmed 272 of the 322 (84%) assignments, see Supplementary Table S1.

## 3. Discussion

Supplementary Figure S1 illustrates the process we use to analyze complex natural products such as botanicals, essential oils, herbs, and spices caused by environmental and/or manufacturing perturbations. In this study, we were interested in learning how sensory-active and health beneficial compounds in pu-erh tea differ in plants grown at high (1651 m) and low (1162 m) elevations. Since the leaves are processed in the same manner, differences in chemistry are primarily due to differences in temperature (Δ3 °C). Target compounds are leaf metabolites that survived farmer processing. Untargeted compounds are those produced by the fire-heated kettle process when making pu-erh tea. Spectral deconvolution of the target (environmental database) compounds, ~60% (187/317 and 166/280) of the constituents in each sample, followed by subtraction of their spectra simplified the analysis.

**Figure 3:**
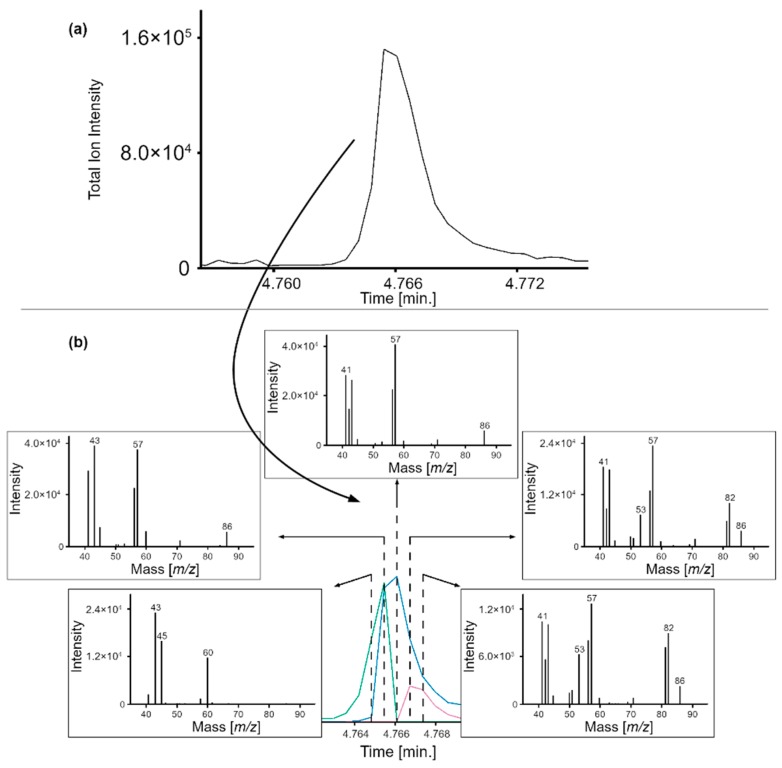
(**a**) Targeted analysis example of high elevation tea. (**b**) Spectral deconvolution of acetic acid (green), hexane (blue), and 2-methylfuran (pink) ions and relative abundances.

Figure 3 shows the TIC peak of three coeluting target compounds (acetic acid, 2-methylfuran, and hexane) in high elevation tea. Although the spectrum at each peak scan is different (Figure 3b), Ion Analytics correctly identified each analyte by deconvolving their spectral signature. Shown in the bottom panel are the RIC peaks for acetic acid (green), 2-methylfuran (pink), and hexane (blue), whose identities were confirmed using reference standards. The RIC peaks provide the means to measure the relative distribution of each analyte in the sample.

**Figure 4:**
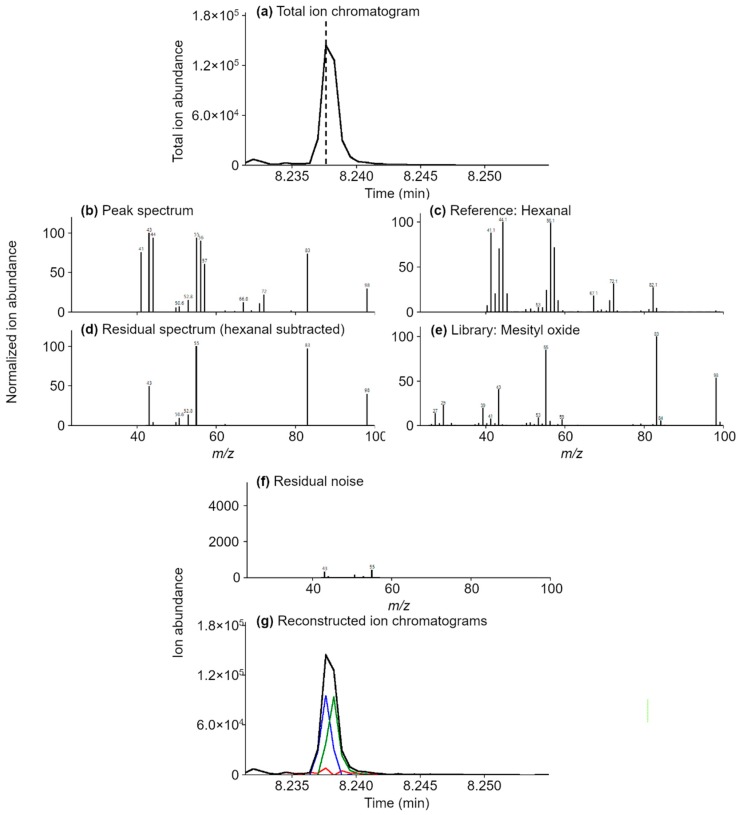
Untargeted analysis example, high elevation tea. Spectral deconvolution and MS subtraction of hexanal, the target compound, ions and relative abundances (**c**) from the TIC (**a**) peak spectra (**b**) yields the residual spectrum (**d**) for mesityl oxide (**e**). Subtraction of the ions and relative abundances of both compounds results in baseline noise (**f**,**g**). The RIC chromatograms for hexanal (blue) and mesityl oxide (green) are also in (**g**). Experimental spectra were acquired in the range of 40–280 *m*/*z*, and therefore, ions below 40 *m*/*z* are missing from spectra (**b**–**d**,**f**).

Figure 4 is an example of an untargeted analysis where the spectrum (c) for the target compound, hexanal, is subtracted from the TIC (a), see example spectra (b), exposing the spectrum of an unknown. Since spectrum (d) is constant at each peak scan, the MS subtraction algorithm reveals another compound. The retention time and mass spectrum match mesityl oxide (e). Subtracting the mesityl oxide reference spectrum from the remaining signal yields baseline noise (f and g), positively confirming the peak assignments and that no other compounds elute in the corresponding peak-region. Also shown in (g) are the RIC peaks for both hexanal (blue) and mesityl oxide (green).

This approach reduces the complexity of each subsequent analysis by using the results of preceding samples as target compounds. By annotating the database, analytes can be tracked independent of sample type and from sample-to-sample, year-to-year. Both software provide data to analyze statistically. How one starts depends on the goal of the investigation. How one ends depends on whether speciation provides meaningful input into the system under investigation, see below.

### Effects of Processing and Elevation on Tea

The most flavorful teas in Yunnan, China, are those grown at the highest elevations [20,21]. Earlier, we showed that high compared to low elevation “environmental” teas contained more and higher concentrations of metabolites that exhibit sweet, floral notes [1]. We assumed, therefore, that the farmer-processed pu-erh tea would as well. The results of this study support this finding. The high elevation tea retained more of the sensory-pleasing environmental metabolites than the low elevation tea, which possessed more of the earthy, harsher tasting aromatics. The comparative chromatogram, depicted in Figure 1, shows more magenta peaks overall, indicating a decrease in volatiles in the low elevation pu-erh compared to the high. Further examination with Ion Analytics showed that of the 275 common analytes, 134 were higher and 19 were lower in abundance in the high elevation tea at the 95% confidence interval of the average relative percent difference, namely, 23% ± 46% (*n* = 2 samples), with 119 and 60 higher than 100% and 200%, respectively. We found substantial concentration differences in the oxygenated monoterpenes. The high elevation tea contained more of the *trans*- (14000%) and *cis*- (3400%) furanoid linalool oxides, compounds characterized as sweet and floral, than the low elevation tea. Similarly, the *cis*- (700%) and *trans*- (750%) pyranoid linalool oxides, which exhibit citrusy, woody notes, were also higher in concentration.

Table 2 lists the 42 compounds unique to high elevation pu-erh and the five compounds unique to low elevation pu-erh, along with their sensory and/or health beneficial properties. Although high elevation spring teas are higher in quality, the occurrence of the summer monsoon rains offsets the elevational (temperature) effect, resulting in less desirable tea, as evidenced by farmers receiving 50% less for the summer compared to spring teas [22]. These findings illustrate why it is important to measure the total volatile profile, especially when evaluating the health benefits of green tea in clinical trials [23,24,25,26].

## 4. Materials and Methods 

### 4.1. Sample Collection

Tea samples (var. *assamica*) were collected from Nannuo Mountain, Menghai County in Yunnan Province, China, in 2014. Low elevation teas, grown at 1162 m, were collected from Xiang Yang Village from June 8—10. High elevation teas, grown at 1651 m, were collected from Ya Kou Old Village from June 10—12. Elevation differences correspond to a 3 °C cooler temperature at high elevation (https://www.spc.noaa.gov/exper/soundings/help/lapse.html). Farmers at the study site processed the samples as green, pu-erh tea [2,22].

### 4.2. Sample Preparation and HS-SPME

Added to a 20 mL glass vial, thermostatted at 50 °C, were 1.5 g of dried plant material and 2 mL of ultrapure water. Volatiles were sampled in the headspace of the vial by solid phase microextraction (SPME) for 50 min. The fibers were coated with 50/30 µm thick divinylbenzene/carboxen on polydimethyl siloxane (PDMS), which was 2 cm long (Supelco, Bellefonte, PA, USA). Fibers were preconditioned according to the manufacturer before the addition of internal standards, α- and β-thujone, which were sampled by exposing the SPME to 5 µL of a stock solution at 1000 mg/L for 20 min at 50 °C. The internal standards were used to normalize the GC×GC/MS peak responses. Two replicates were prepared per sample.

### 4.3. GC×GC/MS Instrumentation

Tea samples were analyzed using a Gerstel (Mülheim an der Ruhr, Germany) MPS-2 multipurpose sampler and Agilent (Santa Clara, CA, USA) models 6890 GC and 5975C MS. The MS, operated in electron ionization mode at 70 eV, scanned from 40 to 280 *m/z* at 30 Hz. The GC×GC was equipped with a two-stage KT 2004 loop thermal modulator (Zoex Corporation, Houston, TX, USA), which was cooled by liquid nitrogen. The hot jet pulse time was 250 ms. The modulation period was 4 s. A mass flow controller linearly reduced the cold-jet total flow from 40% to 8% by the end of the run. A deactivated fused silica capillary loop (1 m × 0.1 mm d_c_) transferred sample portions from the first to the second column. The first column was an SE52 column (95% PDMS, 5% phenyl, 30 m × 0.25 mm × 0.25 µm), which was coupled to an OV1701 column (86% PDMS, 7% phenyl, 7% cyanopropyl, 1 m × 0.1 mm × 0.10 µm) as the second column. Both columns were purchased from Mega (Legnano, Milan, Italy). The temperature program was 50 °C (1 min) to 210 °C at 3 °C/min, then at 280 °C (10 min) at 10 °C/min. The SPME fiber was thermally desorbed into the split/splitless injector for 5 min using a split ratio of 1:5. Helium served as the carrier gas, operating at constant flow (1.3 mL/min) with an initial head pressure of 298 kPa.

### 4.4. GC-GC/MS Instrumentation

Heart-cutting GC-GC/MS instrumentation and parameters for tea library-building have been described in detail in our previous works [1,2,3,19]. Briefly, PDMS-coated stir bars (Gerstel, Mülheim an der Ruhr, Germany) were used to extract volatiles from aqueous tea infusions. A thermal desorption unit (TDU, Gerstel) was used to provide splitless transfer of the sample into the programmable temperature vaporization inlet (CIS, Gerstel), held at −100 °C. The TDU was heated from 40 °C (0.70 min) to 275 °C (3 min) at 600 °C/min under 50 mL/min helium gas flow. After 0.1 min, the CIS was heated to 275 °C at 12 °C/min and held for 5 min. The first GC (Agilent 6890, Santa Clara, CA, USA) housed column 1 (C1, 30 m × 250 µm × 0.25 µm Rtx-Wax, Restek, Bellefonte, PA, USA) and was equipped with a flame ionization detector. The temperature of C1 was programmed from 40 °C (1 min) to 240 °C at 5 °C/min. C1 was connected to the CIS with a TDU on one end and to a five-port crosspiece (Gerstel) on the other. The second oven (Agilent 6890) housed column 2 (C2, 30 m × 250 µm × 0.25 µm Rxi-5MS, Restek), which was connected to the crosspiece through a cryogenic freeze trap (CTS1, Gerstel) on one end and to the MS (Agilent 5975) on the other. The oven temperature was held at 40 °C for 1 min, and then increased to 280 °C at a rate of 5 °C/min. Both columns operated at 1.2 mL/min constant helium flow. The MS operating conditions were: 70 eV electron ionization source, 230 °C ion source, 150 °C quadrupole, and 40 to 250 *m*/*z* scan range. A multipurpose autosampler (MPS, Gerstel) was used for automated sample injection, and a multicolumn switching device (MCS, Gerstel) supplied countercurrent flow to the crosspiece. A heart-cut was made each minute for a total of 40 heart-cuts per sample.

### 4.5. Data Analysis

GC Image v 2.8 (LLC, Lincoln, NE, USA) was used for untargeted/targeted fingerprinting based on peak-region features and for comparative visualization of composite chromatogram pairs for high and low elevation tea samples. Two replicate preparations of each sample were used in this approach. The software identified compounds when the LRI match was within ± 10 units of published values and the NIST spectral match for that compound was >950. After the analysis, positively matched peaks were used to re-align chromatograms [47,48] and combined into a single, composite chromatogram. 2D-peak detection was performed on the composite chromatogram with peak outlines recorded as peak-region objects in the chromatographic plane. Then, all metadata belonging to positively matched peaks and peak-region objects (chemical names, retention times, *m*/*z* fragmentation pattern, retention index, and additional information about matching results) were combined into a template of features.

We used the Ion Analytics software (Gerstel, Mülheim an der Ruhr, Germany and Andover, MA, USA) as follows. First, we selected the environmental tea database to target known compounds in unprocessed tea by spectral deconvolution of the chromatogram. For each target compound, the normalized ion intensity, $I_{i}\left(t\right)$ (relative to the user-defined main ion, usually the base ion, specified by i=1), at scan (t) is

(1) $$I_{i}\left(t\right)=\frac{A_{i}\left(t\right)}{R_{i}A_{1}},$$

where $A_{i}\left(t\right)$ is the i-th qualifier ion intensity at scan (t) and $R_{i}$ is the expected relative ion abundance ratio obtained from reference standards or NIST, Wiley, Adams, etc. All qualifier ions are normalized to the main ion ($I_{1}=1$). The spectral match, ΔI, is calculated as:

(2) $$\Delta I=\frac{\sum_{i=1}^{N-1}\sum_{j=i+1}^{N}Abs\left(I_{i}-I_{j}\right)}{\sum_{i=1}^{N-1}i},$$

where ΔI is the average normalized ion intensity deviation of each of the N specified qualifier ions. As ΔI approaches zero, the quality of the match increases. Between three to five ions, including the main ion, were used for each compound. Another constraint is that the scan-to-scan variance, ΔE, must be ≤ 7, and is calculated as:

(3) $$\Delta E=\Delta I\cdot \log A_{1},$$

Additive ion signal due to coelution is eliminated by comparing all ion ratios against each other. The relative error is computed at each scan. If the ion ratio exceeds the expected ratio, and if all other ions are in agreement, the residual signal is subtracted from the matrix-affected ion. An acceptable match fits the criterion:

(4) $$\Delta I\leq K+\frac{\Delta_{0}}{A_{1}},$$

where K is the adjustable percentage difference and $\Delta_{0}$ is the cumulative error from background signal and/or instrument noise. Target compound identification occurs when ΔE or ΔI is ≤7 in at least three consecutive peak scans. Additionally, the qualifier ion ratio deviation must be ≤ 20% at each scan across the peak to ensure consistency across the peak. The *Q*-value must be ≥90. The *Q*-value measures compound hit quality on a scale of integers 1 to 100, where a higher value indicates a higher quality match between sample and library spectra. The *Q*-value is determined as the maximum of either 100−D or 1, where D is calculated as:

(5) $$D=\frac{100\sum_{i=1}^{N}\left|r_{i}^{e}-r_{i}^{0}\right|\cdot {\left(\log\left(100\cdot r_{i}^{e}+1\right)\right)}^{2}\;}{21.3\sum_{i=0}^{N}r_{i}^{e}},$$

where $r_{i}^{e}$ is the expected qualifier ion ratio for the i-th qualifier ion, $r_{i}^{0}$ is the observed qualifier ion ratio for the i-th qualifier ion, and N is the number of qualifier ions. The *Q*-ratio must also be within ≤ 20% of the relative abundance. The *Q*-ratio is the peak area ratio of the extracted i-th and main ions, calculated as:

(6) $$D=\frac{100\sum_{i=1}^{N}\left|r_{i}^{e}-r_{i}^{0}\right|\cdot {\left(\log\left(100\cdot r_{i}^{e}+1\right)\right)}^{2}\;}{21.3\sum_{i=0}^{N}r_{i}^{e}},$$

where $S_{i}$ is the peak area extracted for the i-th common ion over the hit and $S_{1}$ is the peak area for the main ion over the hit. Since GC×GC produces multiple modulated peaks per compound, the deconvolution algorithm searches for up to five peaks per compound by default, which can be increased when high concentration analytes are found in the sample. 

Then, the mass spectrum of each target compound was subtracted from corresponding peak scans to reveal untargeted compounds. Peak assignments were made by matching sample and library spectra and retention indices, which were then confirmed using available reference standards. If assignments were not possible, numerical identifiers were used to assign peak names. Finally, once all peak assignments were made, the mass spectrum for each compound was subtracted from the TIC chromatogram to reveal missed peaks. This was established when residual signals approximated the baseline (background noise) of the chromatogram.

## 5. Conclusions

Our overall objective is to demonstrate two complimentary data analysis approaches to differentiating complex samples using tea as the model system. First, we used molecular feature detection to show which regions of the chromatograms differed, then untargeted/targeted analysis to identify all compounds in the sample, both in regions of the chromatogram that differed significantly and where it did not. The latter showed the quantifiable differences at the molecular level in high and low elevation tea. From a sensory perspective, our findings point to compounds consistent with local perspectives of quality in high and low elevation green tea. In this paper, we used comprehensive GC×GC/MS, rather than GC/MS or GC-GC/MS, to produce data on the same time scale as GC/MS with the resolution of GC-GC/MS, without taking days to produce data on one sample. The technology and approach taken differentiates complex samples at the molecular level, critically important to the study of systems biology.

## Figures and Tables

**Table 1:** Targeted and untargeted compounds found by Ion Analytics.

	High Elevation		Low Elevation	
Identity Level	Targeted	Untargeted	Targeted	Untargeted
Positive	92	15	82	13
Tentative	78	54	69	43
* Unknown	17	61	15	58
Total	187	130	166	114

* Unknowns are assigned a numerical identifier in the database.

**Table 2:** Unique compounds in high and low elevation pu-erh tea and their sensory active and/or health beneficial properties.

High Elevation Compounds	Aroma *	Health Benefits
furfural	woody, almond, baked bread	—
18	—	—
(2E)-hexenal	green, banana, aldehydic	antimicrobial [27]
2-furanmethanol	sweet, caramel, burnt	—
(2E)-hexenol	leafy, fruity, unripe banana	—
2-heptanol	fruity, oily, fatty	—
2,5-dimethylpyrazine	cocoa, roasted nuts	—
2(5H)-furanone	buttery	—
heptanol	musty, leafy, herbal, peony	cardioprotective [28]
(3E)-hexenoic acid	fruity, honey, acidic	—
101	—	—
(3Z)-hexenyl acetate	green, banana, apple	—
heptanoic acid	rancid, sour, sweat	—
2-methoxyphenol	phenolic, smoke, spice	—
maltol	caramel, cotton candy, fruity	antianxiety [29], antioxidant [30]
114	—	—
511	—	—
(3Z)-hexenyl butyrate	green apple, fruity, wine	—
(2E)-hexenyl butyrate	green, apricot, ripe banana	—
hexyl butyrate	fruity, apple, waxy	—
512	—	—
514	—	—
nerol	neroli, citrus, magnolia	antibacterial [31], antifungal [32], antinociceptive/anti-inflammatory [33]
(3Z)-hexenyl valerate	apple, kiwi, unripe banana, tropical	—
(3Z)-hexenyl isovalerate	green apple, tropical, pineapple	—
phenylethyl acetate	rose, fruity	—
pentyl hexanoate	pineapple, apple, pear	—
1-nitro-2-phenyl ethane	floral, spice	cardioprotective [34]
γ-nonalactone	coconut, creamy, waxy, buttery	—
(3Z)-hexenyl hexenoate	waxy, pear, winey, grassy, pineapple	—
hexyl hexanoate	fresh cut grass, vegetable	—
(2E)-hexenyl caproate	cognac, herbal, waxy	—
(Z)-jasmone	floral, woody, herbal, spicy	antibacterial [35], anticancer [36]
(E,E)-α-farnesene	citrus, lavender, bergamot,	—
2,4-di-tert-butylphenol	phenolic	antioxidant [37]
δ-cadinene	thyme, woody	—
(Z)-calamenene	herb, spice	antimalarial [38], antitumor [39]
dodecanoic acid	fatty, coconut, bay oil	cardioprotective [40], antibacterial/anti-inflammatory [41]
caryophyllene oxide	woody, spicy	anticancer/analgesic/anti-inflammatory [42]
τ-muurolol	herbal, spicy, honey	antibacterial [43], antioxidant [44]
α-cadinol	herbal, woody	antibacterial/antioxidant [43], anticancer [45], anti-inflammatory [45]
bancroftinone	—	—
**Low Elevation Compounds**	Aroma *	**Health Benefit**
ethyl acetate	weedy, green	—
isoamyl alcohol	alcoholic, banana	antifungal [46]
(2E)-pentenal	pungent, green apple, orange, tomato	—
m-ethyltoluene	—	—
118	—	—

* Aroma information was obtained from The Good Scents Company (http://thegoodscentscompany.com/).

## Supplementary Materials

The following are available online, Figure S1: Ion Analytics and GC Image workflow schematic for the analysis of targeted and untargeted compounds, Table S1: Features detected by GC Image and confirmatory analysis, Table S2: Compounds identified by Ion Analytics. The unprocessed GC×GC /MS data can be found at: http://dx.doi.org/10.25833/jxyj-8w58.
