# Supplementary material for: Untargeted/Targeted 2D Gas Chromatography/Mass Spectrometry Detection of the Total Volatile Tea Metabolome
Source: Molecules. 2019 Oct 18;24(20):3757. doi: 10.3390/molecules24203757 (PMC6832143; doi:10.3390/molecules24203757)
Supplement: Supplementary file 1 [file molecules-24-03757-s001.pdf]

Supplementary material

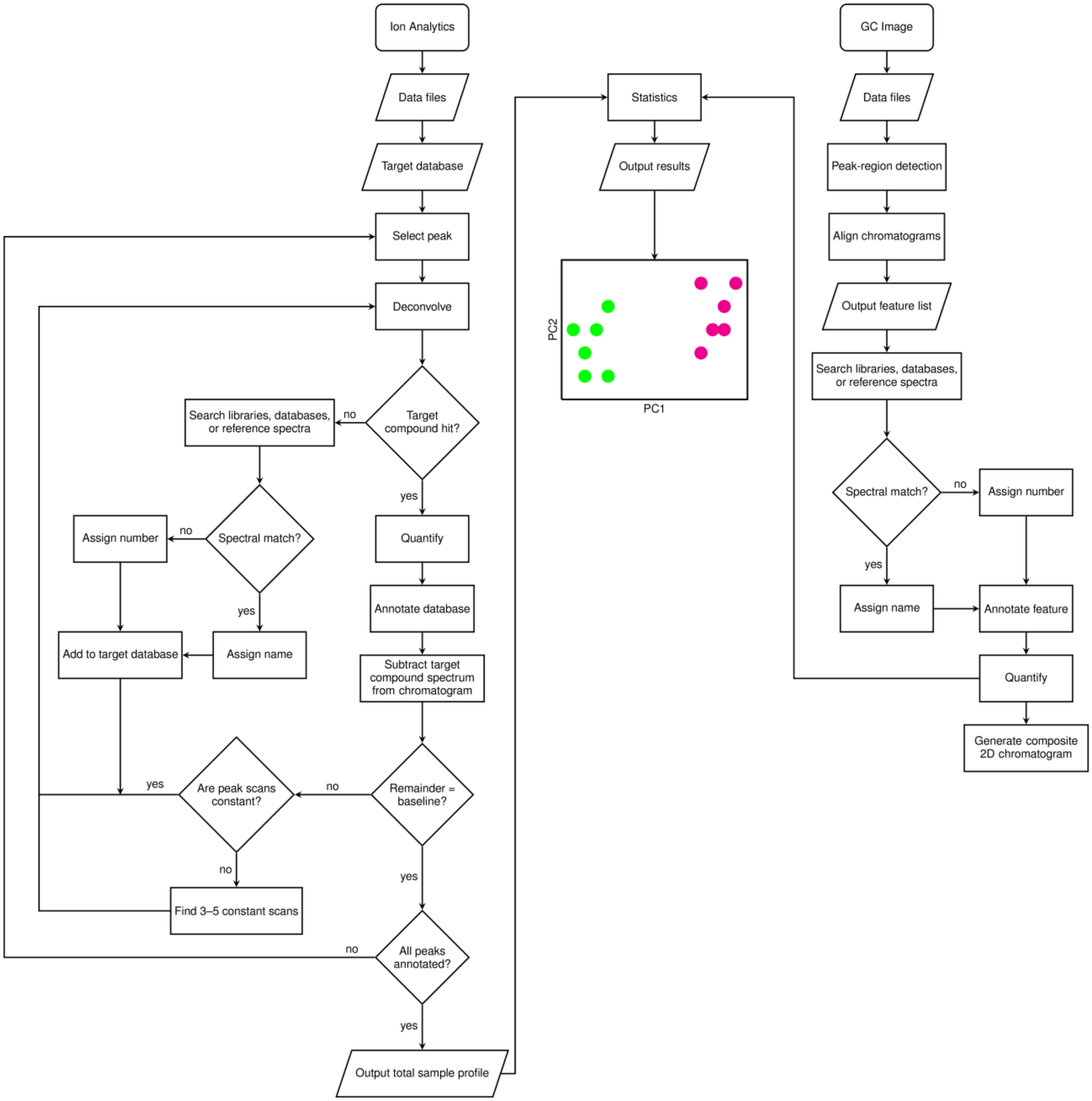

Figure S1. Ion Analytics and GC Image workflow schematic for the analysis of targeted and untargeted compounds

Table S1. Features detected by GC Image and confirmatory analysis

			Volume				Percent Response			
**Compound Name**	**RT 1D (min)**	**RT 2D (s)**	**HM_1**	**HM_2**	**LM_1**	**LM_2**	**HM_1**	**HM_2**	**LM_1**	**LM_2**
(387)	3.6879	1.5794	128851	60764	88018	59701	0.013	0.006	0.009	0.006
(409)	3.6879	2.4019	199545	88818	136440	85023	0.020	0.009	0.014	0.009
(412)	3.6879	3.2617	186262	84481	128164	82253	0.018	0.008	0.013	0.008
(417)	3.6879	3.1308	69015	36185	44010	31005	0.007	0.004	0.004	0.003
(418)	3.6879	2.7477	179661	84264	118668	78152	0.018	0.008	0.012	0.008
(421)	3.6879	1.7477	127290	57034	83363	49563	0.013	0.006	0.008	0.005
(427)	3.6879	3.5794	152499	70770	99250	62448	0.015	0.007	0.010	0.006
(430)	3.6879	2.0935	113025	52871	74864	46346	0.011	0.005	0.008	0.005
(432)	3.6879	2.6075	92846	45619	64151	41420	0.009	0.005	0.007	0.004
(437)	3.6879	3.8318	128748	63624	90193	58953	0.013	0.006	0.009	0.006
(447)	3.6879	1.4393	124668	61680	86702	58078	0.012	0.006	0.009	0.006
(452)	3.6879	1.1682	78976	36996	54192	34834	0.008	0.004	0.006	0.004
(459)	3.6879	1.9813	46306	23913	32447	19889	0.005	0.002	0.003	0.002
(470)	3.6879	0.8318	43837	23413	36762	20068	0.004	0.002	0.004	0.002
(413)	3.7213	0.6636	186204	98510	129354	88097	0.018	0.010	0.013	0.009
(441)	3.7213	0.3645	99149	53767	68954	49051	0.010	0.005	0.007	0.005
(451)	3.7213	0.9439	80174	40913	46442	36383	0.008	0.004	0.005	0.004
(453)	3.7213	0.1308	84533	45512	56828	40279	0.008	0.005	0.006	0.004
(454)	3.7213	1.0561	45541	25234	37435	22578	0.004	0.003	0.004	0.002
2,3-Butanedione* (11)	4.2046	0.1028	975895	3319080	2450161	1597105	0.096	0.332	0.249	0.161
(152)	4.2046	3.7290	782945	166682	262750	236772	0.077	0.017	0.027	0.024
(166)	4.2046	3.4019	3308398	247319	392562	363584	0.327	0.025	0.040	0.037
Dimethyl sulfide (157)	4.2379	1.3364	454787	366093	503736	512900	0.045	0.037	0.051	0.052
(174)	4.2379	2.0561	266216	206343	282585	275326	0.026	0.021	0.029	0.028
(188)	4.2379	1.8411	310323	219772	318514	301818	0.031	0.022	0.032	0.030
(185)	4.2546	2.9065	128662	96476	147371	129110	0.013	0.010	0.015	0.013
(186)	4.2546	2.3925	202551	155174	230483	206090	0.020	0.016	0.023	0.021
(199)	4.2546	2.7196	267460	195228	275545	252690	0.026	0.020	0.028	0.025
Acetone* (18)	4.2713	0.6262	2133748	3695207	4015256	3767929	0.211	0.370	0.409	0.380
(171)	4.2713	3.1121	283199	217459	296569	306552	0.028	0.022	0.030	0.031
Acetic acid* (43)	4.5213	0.5701	190281	836705	314422	557530	0.019	0.084	0.032	0.056
(605)	4.5213	3.1589	29981	4908	21014	21403	0.003	0.000	0.002	0.002
(316)	4.6713	0.0748	83083	60523	65259	55669	0.008	0.006	0.007	0.006
(402)	4.7046	0.1869	61996	67618	45498	35882	0.006	0.007	0.005	0.004
(693)	4.7046	3.2991	27590	21369	20782	17897	0.003	0.002	0.002	0.002
(740)	4.7046	2.8224	64525	50529	51286	40450	0.006	0.005	0.005	0.004
(882)	4.7046	2.3925	34367	39687	28179	20622	0.003	0.004	0.003	0.002
(247)	4.7213	1.2243	93414	58750	70253	66942	0.009	0.006	0.007	0.007

Compound Name	RT 1D (min)	RT 2D (s)	Volume HM_1	HM_2	LM_1	LM_2	Percent Response HM_1	HM_2	LM_1	LM_2
(268)	4.7213	1.3832	115510	75901	92596	96922	0.011	0.008	0.009	0.010
(295)	4.7213	1.6262	137119	114526	131254	123095	0.014	0.011	0.013	0.012
(478)	4.7213	2.0841	46921	59746	50772	44178	0.005	0.006	0.005	0.004
2-Methylfuran (1785)	4.7379	0.7850	670405	647660	566261	605281	0.066	0.065	0.058	0.061
Hexane* (26)	4.7379	0.6449	2890427	1301428	1335304	1299899	0.285	0.130	0.136	0.131
(683)	4.7879	3.7009	30126	13653	18920	17672	0.003	0.001	0.002	0.002
(829)	4.7879	3.4953	43627	22156	26259	23635	0.004	0.002	0.003	0.002
(884)	4.8213	3.1682	30862	11499	16967	14084	0.003	0.001	0.002	0.001
(1025)	4.8546	2.9813	22037	11702	14121	12176	0.002	0.001	0.001	0.001
(960)	4.8713	2.7103	44354	18820	26047	23852	0.004	0.002	0.003	0.002
(304)	4.9213	0.2243	99014	86175	78431	58811	0.010	0.009	0.008	0.006
2-Methyl-1,3-pentadiene (173)	5.1213	0.8318	222216	176631	115439	117865	0.022	0.018	0.012	0.012
(1178)	5.2213	2.7477	11509	7708	8623	8545	0.001	0.001	0.001	0.001
(357)	5.3546	1.8224	43442	41853	50797	64046	0.004	0.004	0.005	0.006
(358)	5.4213	2.7570	77858	50040	86543	94049	0.008	0.005	0.009	0.009
(389)	5.4213	2.6075	44035	37853	53881	56154	0.004	0.004	0.005	0.006
2-Methylpentene* (52)	5.4379	0.8972	977558	773362	947044	1001249	0.097	0.077	0.096	0.101
(356)	5.4379	3.1776	105345	73620	131667	133789	0.010	0.007	0.013	0.013
(372)	5.4379	3.4206	45635	24782	49658	50410	0.005	0.002	0.005	0.005
3-Methylbutanal* (39)	5.4546	0.4019	562200	589451	678859	532983	0.056	0.059	0.069	0.054
(349)	5.4546	3.6822	70275	40823	87949	80666	0.007	0.004	0.009	0.008
(367)	5.4546	3.5701	29090	15987	35551	35376	0.003	0.002	0.004	0.004
Cyclohexane (19)	5.5213	0.2056	194423	79482	1509736	986937	0.019	0.008	0.154	0.099
(368)	5.5213	2.3178	115435	101835	138034	135268	0.011	0.010	0.014	0.014
(400)	5.5379	2.2150	39065	40256	52496	52373	0.004	0.004	0.005	0.005
1-Penten-3-ol* (44)	5.6879	0.5701	713494	775236	698782	652505	0.070	0.078	0.071	0.066
(16)	5.6879	1.7850	530813	1922812	2748072	1926809	0.052	0.193	0.280	0.194
(956)	5.6879	3.0280	16585	14615	20661	16578	0.002	0.001	0.002	0.002
482 (653)	5.7879	0.2243	19625	66363	25858	18874	0.002	0.007	0.003	0.002
Pentanal* (93)	5.8713	0.5607	301002	264778	308919	205419	0.030	0.027	0.031	0.021
(1066)	5.8879	3.8411	47233	17845	25552	25186	0.005	0.002	0.003	0.003
(262)	5.9046	1.6729	4265904	155074	174238	125238	0.421	0.016	0.018	0.013
(1119)	5.9046	2.9159	33404	11414	14296	15375	0.003	0.001	0.001	0.002
(231)	5.9213	1.3084	168480	55364	64646	113546	0.017	0.006	0.007	0.011
(1032)	5.9213	3.1028	30070	10312	16393	14840	0.003	0.001	0.002	0.001
(1081)	5.9213	2.6916	34485	12536	16120	14081	0.003	0.001	0.002	0.001
(1034)	5.9379	3.2710	34921	13578	19628	17701	0.003	0.001	0.002	0.002
(1055)	5.9713	3.4766	34718	14410	20558	18686	0.003	0.001	0.002	0.002
(1068)	5.9879	2.4579	54653	18121	21458	19647	0.005	0.002	0.002	0.002
2-Ethylfuran* (79)	6.0213	0.3925	665263	781653	448024	324074	0.066	0.078	0.046	0.033
(1161)	6.0379	2.2430	25714	9236	11335	10440	0.003	0.001	0.001	0.001

			Volume				Percent Response			
**Compound Name**	**RT 1D (min)**	**RT 2D (s)**	**HM_1**	**HM_2**	**LM_1**	**LM_2**	**HM_1**	**HM_2**	**LM_1**	**LM_2**
(1004)	6.0546	0.0654	17570	7622	10510	8528	0.002	0.001	0.001	0.001
(1503)	6.2379	2.7009	20973	11093	12711	9974	0.002	0.001	0.001	0.001
(1596)	6.2379	3.0561	13848	7509	8694	7360	0.001	0.001	0.001	0.001
2-Methylbutanal* (293)	6.2713	0.4299	91135	124322	91680	99681	0.009	0.012	0.009	0.010
2-Methylbutanenitrile (535)	6.3879	0.9159	96825	51935	63082	44510	0.010	0.005	0.006	0.004
(1641)	6.4046	2.3178	13554	6288	7605	6019	0.001	0.001	0.001	0.001
3-Methylbutanol (825)	6.4546	0.7850	45617	31796	27596	23966	0.005	0.003	0.003	0.002
(1613)	6.5213	0.0374	4781	4568	3146	2656	0.000	0.000	0.000	0.000
(1071)	6.5713	1.5234	81508	21598	22783	19949	0.008	0.002	0.002	0.002
(1693)	6.5879	2.4860	10631	6592	7511	6338	0.001	0.001	0.001	0.001
2-Methyl-2-butenal* (103)	6.6379	0.9252	86515	147997	47327	109925	0.009	0.015	0.005	0.011
(1753)	6.6713	3.5981	11722	8543	8656	7697	0.001	0.001	0.001	0.001
(510)	6.6879	0.1963	24286	53708	19201	45342	0.002	0.005	0.002	0.005
(700)	6.6879	0.4953	14677	14884	14358	15258	0.001	0.001	0.001	0.002
(1636)	6.7546	2.2897	12686	8389	8393	8085	0.001	0.001	0.001	0.001
(1226)	6.7713	1.4860	32337	11802	11985	9356	0.003	0.001	0.001	0.001
(E)-3-Penten-2-one (1786)	6.7879	0.9065	237685	150522	207321	147251	0.023	0.015	0.021	0.015
(1328)	6.8046	1.7290	40176	26362	24966	24303	0.004	0.003	0.003	0.002
(1681)	6.9213	2.0374	17682	12883	13356	11263	0.002	0.001	0.001	0.001
2-Methyl-3-pentanone (176)	6.9379	0.8598	93641	32544	62891	24001	0.009	0.003	0.006	0.002
Pyridine (1788)	6.9379	0.9159	173102	133158	120269	138777	0.017	0.013	0.012	0.014
(1204)	6.9546	1.4486	33857	13460	13357	11481	0.003	0.001	0.001	0.001
(1713)	6.9546	2.5421	10126	6775	7255	5964	0.001	0.001	0.001	0.001
(1300)	6.9879	1.6916	14805	9482	9439	9151	0.001	0.001	0.001	0.001
(1570)	7.0379	3.5701	16033	10001	10589	9049	0.002	0.001	0.001	0.001
Dimethyl disulfide (232)	7.0713	0.6729	114308	110100	119841	130675	0.011	0.011	0.012	0.013
(553)	7.1046	0.2991	18095	30764	14515	26742	0.002	0.003	0.001	0.003
(1700)	7.1213	2.9346	8784	7188	6671	5998	0.001	0.001	0.001	0.001
(1697)	7.1879	3.7757	12543	10200	10692	9582	0.001	0.001	0.001	0.001
(1389)	7.2213	0.0467	3868	3315	3118	3388	0.000	0.000	0.000	0.000
Pentanol* (24)	7.3546	1.0374	2296725	1922945	1315765	1195813	0.227	0.193	0.134	0.121
(1563)	7.3713	3.5140	10611	8093	6230	5539	0.001	0.001	0.001	0.001
(1083)	7.3879	1.8785	28560	22074	20162	18062	0.003	0.002	0.002	0.002
(1294)	7.3879	2.6168	30699	24684	22659	22150	0.003	0.002	0.002	0.002
(Z)-2-Penten-1-ol* (40)	7.4046	1.1776	336245	570626	303351	303111	0.033	0.057	0.031	0.031
(1077)	7.4213	2.1776	36302	32893	27037	26945	0.004	0.003	0.003	0.003
(1238)	7.4379	2.4206	24254	20449	18924	17244	0.002	0.002	0.002	0.002
311 (71)	7.4546	0.9159	322980	189374	190729	156028	0.032	0.019	0.019	0.016
(972)	7.4546	0.4393	31491	21468	21560	19780	0.003	0.002	0.002	0.002
(468)	7.4713	0.2150	33780	60272	27684	57913	0.003	0.006	0.003	0.006
Toluene* (8)	7.4879	0.6542	2504525	2244127	2952305	2799800	0.247	0.225	0.301	0.282

Compound Name	RT 1D (min)	RT 2D (s)	Volume HM_1	HM_2	LM_1	LM_2	Percent Response HM_1	HM_2	LM_1	LM_2
(1704)	7.5379	3.5607	17393	9481	13291	8850	0.002	0.001	0.001	0.001
(1749)	7.5879	3.0654	14857	9101	11542	8639	0.001	0.001	0.001	0.001
(1257)	7.5879	0.0561	9730	7576	7590	7411	0.001	0.001	0.001	0.001
(1751)	7.6046	2.6262	8922	5490	7580	5762	0.001	0.001	0.001	0.001
(1447)	7.6213	1.8692	10983	7012	9632	6946	0.001	0.001	0.001	0.001
(1586)	7.6379	3.9159	9307	4730	7459	5605	0.001	0.000	0.001	0.001
2,4-Pentanedione* (299)	7.7879	0.9626	80866	60706	45946	42429	0.008	0.006	0.005	0.004
(582)	7.8046	0.2336	19592	27780	16076	29117	0.002	0.003	0.002	0.003
(1632)	7.8046	1.7944	12404	8748	9362	8712	0.001	0.001	0.001	0.001
(900)	7.8379	1.3178	19105	15232	13558	14130	0.002	0.002	0.001	0.001
(1473)	7.8379	1.5888	9106	5968	8019	6147	0.001	0.001	0.001	0.001
2-Hexanone (258)	7.9546	1.0374	113863	98623	98455	87569	0.011	0.010	0.010	0.009
(1718)	7.9546	1.8411	14746	8719	11276	9204	0.001	0.001	0.001	0.001
Octene (162)	7.9546	0.5421	123129	118385	94246	119729	0.012	0.012	0.010	0.012
2-Propylfuran (1787)	7.9713	0.6729	51992	49367	37309	37788	0.005	0.005	0.004	0.004
(1609)	7.9879	2.7664	9314	6413	7929	7374	0.001	0.001	0.001	0.001
(1649)	8.0213	0.0467	3020	2198	1883	1868	0.000	0.000	0.000	0.000
(513)	8.1546	0.2056	15614	29018	12009	31740	0.002	0.003	0.001	0.003
Octane* (42)	8.2379	0.4766	743110	714146	647031	630756	0.073	0.072	0.066	0.064
(1516)	8.2379	2.1402	18623	11985	12609	11219	0.002	0.001	0.001	0.001
4-Methyl-3-penten-2-one (34)	8.2713	1.0280	860595	779980	804450	663285	0.085	0.078	0.082	0.067
(576)	8.2713	0.2056	13492	18040	10373	17315	0.001	0.002	0.001	0.002
(1571)	8.2713	1.9065	8939	7004	7138	5604	0.001	0.001	0.001	0.001
(1690)	8.4379	2.3458	8690	5400	7074	5378	0.001	0.001	0.001	0.001
(629)	8.4546	0.2617	19088	25853	15537	26967	0.002	0.003	0.002	0.003
(E)-4-Octene (142)	8.6046	0.5701	76847	107894	66358	81252	0.008	0.011	0.007	0.008
(651)	8.6713	0.1869	15351	26043	14322	24427	0.002	0.003	0.001	0.002
Hexanal* (76)	8.6879	0.9439	201049	91580	326102	230426	0.020	0.009	0.033	0.023
(1573)	8.7213	1.8598	24756	19238	18103	17680	0.002	0.002	0.002	0.002
(1667)	8.7213	3.8972	5370	4555	4878	4716	0.001	0.000	0.000	0.000
(85)	8.7546	1.1682	79656	194685	39834	86799	0.008	0.019	0.004	0.009
(1354)	8.7546	1.5234	18740	17479	14739	14273	0.002	0.002	0.002	0.001
2-Ethyl-1H-Pyrrole (88)	8.7713	1.0935	371262	278090	160907	161683	0.037	0.028	0.016	0.016
2-Ethyl-5-methylfuran (110)	8.8379	0.5327	490309	367439	402252	350689	0.048	0.037	0.041	0.035
(718)	8.9713	0.1402	6920	14992	5437	13923	0.001	0.002	0.001	0.001
3-Ethyl-1H-pyrrole (479)	9.0379	0.9907	62652	54064	47953	38200	0.006	0.005	0.005	0.004
2-Methylpyrazine (1342)	9.0546	1.4299	12607	12944	8389	8190	0.001	0.001	0.001	0.001
1,3-Octadiene (204)	9.0713	0.6729	85461	67318	103399	80048	0.008	0.007	0.011	0.008
(1360)	9.1379	2.8224	15351	16137	12238	14781	0.002	0.002	0.001	0.001
3-Methylbutanoic acid (330)	9.1546	2.4673	57904	48788	38334	51323	0.006	0.005	0.004	0.005
(611)	9.1879	0.1776	12590	18234	10260	20020	0.001	0.002	0.001	0.002

			Volume				Percent Response			
Compound Name	RT 1D (min)	RT 2D (s)	HM_1	HM_2	LM_1	LM_2	HM_1	HM_2	LM_1	LM_2
Butyl acetate (583)	9.2379	0.9159	46440	39502	44795	31703	0.005	0.004	0.005	0.003
2-Methyl-2-pentenal* (507)	9.2879	1.2617	31239	35749	18029	21720	0.003	0.004	0.002	0.002
(1330)	9.3546	1.9626	10933	9598	8063	8594	0.001	0.001	0.001	0.001
(1386)	9.3546	0.3738	10076	7953	7279	7891	0.001	0.001	0.001	0.001
Furfural* (877)	9.4046	1.7570	43277	34596	27507	25917	0.004	0.003	0.003	0.003
(382)	9.4046	0.4953	60597	42461	44372	43060	0.006	0.004	0.005	0.004
(429)	9.4046	1.1776	49674	32383	22219	21891	0.005	0.003	0.002	0.002
(1217)	9.4379	0.1682	7409	8081	6004	8969	0.001	0.001	0.001	0.001
(1759)	9.4546	3.0561	5434	5091	4197	4456	0.001	0.001	0.000	0.000
2-Methylbutanoic acid (328)	9.5046	2.4766	60453	54212	63629	58688	0.006	0.005	0.006	0.006
(498)	9.5546	1.0374	31812	23600	36841	30737	0.003	0.002	0.004	0.003
(E)-2-Hexenal* (1403)	9.6046	1.5421	11030	8298	8605	7633	0.001	0.001	0.001	0.001
(1278)	9.6379	0.1682	19102	19649	13745	21122	0.002	0.002	0.001	0.002
(1205)	9.6879	1.4112	22421	18658	14544	16142	0.002	0.002	0.001	0.002
(396)	9.6879	0.4860	57945	40772	41585	42619	0.006	0.004	0.004	0.004
(587)	9.6879	1.9065	29698	27769	27128	25499	0.003	0.003	0.003	0.003
2,5,5-Trimethyl-1-hexen-3-yne (138)	9.7879	0.7103	150255	138766	155999	143963	0.015	0.014	0.016	0.015
(1741)	9.8213	1.3832	9355	5682	5727	5067	0.001	0.001	0.001	0.001
(426)	9.9546	0.4673	24326	24662	23227	22412	0.002	0.002	0.002	0.002
2-Furanmethanol (893)	10.0546	2.1495	63385	56974	14761	16168	0.006	0.006	0.002	0.002
(955)	10.0546	2.2897	19692	22695	7579	10071	0.002	0.002	0.001	0.001
(Z)-3-Hexen-1-ol (302)	10.0879	1.5234	1594426	1619555	83937	82808	0.157	0.162	0.009	0.008
(446)	10.1213	0.4393	68414	58695	46243	50801	0.007	0.006	0.005	0.005
(1633)	10.1213	2.5140	30543	29314	13758	16244	0.003	0.003	0.001	0.002
2-Allylfuran (363)	10.1379	1.0280	49511	51088	24345	39929	0.005	0.005	0.002	0.004
10 (476)	10.1713	0.9252	57562	45437	31109	38899	0.006	0.005	0.003	0.004
(1722)	10.2213	0.1589	16897	16301	9038	10347	0.002	0.002	0.001	0.001
1,3-trans,5-cis-octatriene (364)	10.3046	0.7477	40851	36289	33081	32917	0.004	0.004	0.003	0.003
(193)	10.4379	0.9813	76452	65686	59992	87406	0.008	0.007	0.006	0.009
(484)	10.4379	0.4299	62221	56248	49001	48066	0.006	0.006	0.005	0.005
(1770)	10.4879	3.5981	5139	4790	4070	4321	0.001	0.000	0.000	0.000
(E)-2-Hexen-1-ol* (314)	10.5046	1.5421	502173	522555	45930	51257	0.050	0.052	0.005	0.005
Ethylbenzene* (77)	10.6713	0.9907	137074	124066	98950	184202	0.014	0.012	0.010	0.019
(1114)	10.6879	2.8224	29560	35958	14928	20153	0.003	0.004	0.002	0.002
(495)	10.7046	0.4112	71325	63637	57976	58630	0.007	0.006	0.006	0.006
(698)	10.7046	1.4019	57905	53916	36039	25574	0.006	0.005	0.004	0.003
(1254)	10.7546	0.7009	10239	10372	10541	8388	0.001	0.001	0.001	0.001
Pentanoic acid* (1783)	10.8046	2.8037	11550	7009	8653	6320	0.001	0.001	0.001	0.001
1-Hexanol* (1232)	10.8546	1.4673	15092	14426	9307	9613	0.001	0.001	0.001	0.001
(1019)	10.9213	1.3178	19953	17926	14242	12248	0.002	0.002	0.001	0.001
p-Xylene (147)	10.9379	0.9720	108196	96977	202266	106986	0.011	0.010	0.021	0.011

			Volume				Percent Response			
**Compound Name**	**RT 1D (min)**	**RT 2D (s)**	**HM_1**	**HM_2**	**LM_1**	**LM_2**	**HM_1**	**HM_2**	**LM_1**	**LM_2**
(511)	11.1213	0.3832	40063	36722	34964	36942	0.004	0.004	0.004	0.004
(235)	11.1379	0.8598	34862	29041	78748	60235	0.003	0.003	0.008	0.006
(282)	11.2213	0.9813	40836	46454	43322	46710	0.004	0.005	0.004	0.005
(565)	11.2546	1.4579	33183	32691	9936	15107	0.003	0.003	0.001	0.002
(536)	11.2879	0.3738	20752	17097	17209	16919	0.002	0.002	0.002	0.002
(620)	11.3546	0.7757	19595	18649	25417	20477	0.002	0.002	0.003	0.002
2-Heptanone* (100)	11.4046	1.4579	230898	202378	33591	152491	0.023	0.020	0.003	0.015
2(5H)-Furanone (521)	11.4879	0.3645	39318	34991	23915	32095	0.004	0.004	0.002	0.003
2-Butylfuran (230)	11.5379	0.9907	90756	87645	64781	57373	0.009	0.009	0.007	0.006
Methoxy phenyl oxime (132)	11.5713	3.8972	2257133	163468	208208	138832	0.223	0.016	0.021	0.014
(622)	11.5879	2.0187	31207	28941	23856	33652	0.003	0.003	0.002	0.003
Styrene* (9)	11.6046	1.2056	1093559	1088635	2623663	2585436	0.108	0.109	0.267	0.261
(1348)	11.6379	2.3458	10426	8096	7586	9403	0.001	0.001	0.001	0.001
(1401)	11.6379	2.5607	9867	8001	6012	7965	0.001	0.001	0.001	0.001
(1521)	11.6713	2.8879	18094	14709	13045	15465	0.002	0.001	0.001	0.002
(554)	11.7379	0.3645	31494	16931	17249	14518	0.003	0.002	0.002	0.001
(89)	11.7713	0.7290	180056	145114	183583	186246	0.018	0.015	0.019	0.019
(1483)	11.7879	2.1589	8311	6881	8527	6457	0.001	0.001	0.001	0.001
Heptanal* (156)	11.8046	1.4766	223059	195538	110780	98815	0.022	0.020	0.011	0.010
(579)	11.9546	0.3364	24192	20645	22066	19814	0.002	0.002	0.002	0.002
(592)	12.0379	0.3364	22583	18196	17959	19826	0.002	0.002	0.002	0.002
Nonane (1341)	12.0379	0.7944	14199	12619	34406	11827	0.001	0.001	0.004	0.001
2-Heptanol* (431)	12.0713	1.6449	23601	23006	23969	33612	0.002	0.002	0.002	0.003
(1322)	12.1213	1.9439	9855	8178	7740	8595	0.001	0.001	0.001	0.001
483 (58)	12.1546	1.1495	163126	150516	462717	409033	0.016	0.015	0.047	0.041
(1343)	12.2213	0.8224	8596	9308	9180	8403	0.001	0.001	0.001	0.001
Amyl acetate (395)	12.3046	1.3084	71965	66025	43574	35991	0.007	0.007	0.004	0.004
Propylpropanoate (301)	12.3379	1.1121	36590	34412	146171	58219	0.004	0.003	0.015	0.006
2-Acetylfuran (463)	12.3546	2.0467	55760	49715	35830	35759	0.006	0.005	0.004	0.004
(504)	12.3879	2.0000	24946	19010	13185	16713	0.002	0.002	0.001	0.002
Butyrolactone* (567)	12.4879	3.6262	78479	53428	34239	37539	0.008	0.005	0.003	0.004
(1456)	12.5213	0.7944	8309	7793	6832	6889	0.001	0.001	0.001	0.001
(1365)	12.5713	1.5047	15102	13893	9473	10441	0.001	0.001	0.001	0.001
484 (410)	12.5879	1.1215	76994	69278	94024	80343	0.008	0.007	0.010	0.008
(925)	12.5879	0.0935	15044	12938	11072	12464	0.001	0.001	0.001	0.001
(1402)	12.5879	1.9720	20862	19378	20573	16183	0.002	0.002	0.002	0.002
(1750)	12.6046	0.6449	9451	7879	7724	7795	0.001	0.001	0.001	0.001
2,5-Dimethyl-pyrazine (588)	12.7879	1.2804	18661	18776	10203	14902	0.002	0.002	0.001	0.002
(1187)	12.8213	1.5701	12268	12750	8571	10498	0.001	0.001	0.001	0.001
(733)	12.9879	1.4299	17312	14656	16795	16075	0.002	0.001	0.002	0.002
(1135)	13.0046	1.5607	10549	9631	5909	6480	0.001	0.001	0.001	0.001

			Volume				Percent Response			
Compound Name	RT 1D (min)	RT 2D (s)	HM_1	HM_2	LM_1	LM_2	HM_1	HM_2	LM_1	LM_2
(1612)	13.0046	0.7477	6949	6344	7053	6752	0.001	0.001	0.001	0.001
(1267)	13.0546	2.0467	10038	10207	8840	9754	0.001	0.001	0.001	0.001
(442)	13.0713	0.9813	59157	52762	63151	65444	0.006	0.005	0.006	0.007
(1706)	13.1046	1.7290	9801	7796	7974	8013	0.001	0.001	0.001	0.001
(1728)	13.1379	1.8785	6584	5523	5848	5436	0.001	0.001	0.001	0.001
485 (111)	13.3379	1.2150	163633	153665	89674	136446	0.016	0.015	0.009	0.014
(Z)-4-Heptenal* (1105)	13.4046	1.8598	18945	18584	11564	13877	0.002	0.002	0.001	0.001
α-Pinene (119)	13.4046	0.9159	109318	104483	92958	119911	0.011	0.010	0.009	0.012
(1284)	13.5379	1.6449	22741	18819	17761	17077	0.002	0.002	0.002	0.002
3,5-Dimethylphenol (645)	13.5713	0.9159	12481	10312	61197	14441	0.001	0.001	0.006	0.001
486 (105)	13.6379	1.2243	264567	258948	223881	217243	0.026	0.026	0.023	0.022
(677)	13.7879	0.9439	15568	14640	18154	15924	0.002	0.001	0.002	0.002
3-Isopropylcyclohexene (475)	13.9213	1.1963	27163	31313	41021	32530	0.003	0.003	0.004	0.003
316 (616)	13.9713	2.1121	38607	21631	11399	19302	0.004	0.002	0.001	0.002
(1091)	14.0713	2.2804	12640	11171	8565	12556	0.001	0.001	0.001	0.001
Camphene (343)	14.1213	1.0000	60520	57633	56916	50487	0.006	0.006	0.006	0.005
6-Methyl-2-heptanone (280)	14.2046	1.6168	37027	32147	33132	41115	0.004	0.003	0.003	0.004
Propylbenzene (353)	14.2379	1.3084	36751	34632	33949	38517	0.004	0.003	0.003	0.004
(E)-2-Heptenal* (98)	14.2713	1.9159	130923	108902	180234	151932	0.013	0.011	0.018	0.015
(1784)	14.2879	3.0280	5314	5150	4527	4944	0.001	0.001	0.000	0.000
(1012)	14.3213	2.2710	8270	7578	6205	8221	0.001	0.001	0.001	0.001
(728)	14.3713	1.1121	22155	22726	26137	14894	0.002	0.002	0.003	0.002
(419)	14.4379	1.3084	22286	21192	15315	21961	0.002	0.002	0.002	0.002
(398)	14.4713	0.8411	35268	32404	31085	31626	0.003	0.003	0.003	0.003
(1566)	14.5213	3.7664	6888	6021	5269	6219	0.001	0.001	0.001	0.001
(1501)	14.5713	3.5794	13098	11297	8888	10561	0.001	0.001	0.001	0.001
(1558)	14.5713	3.2710	9791	9852	5243	7201	0.001	0.001	0.001	0.001
Benzaldehyde* (32)	14.5879	2.0654	1583428	1484686	412206	765152	0.156	0.149	0.042	0.077
(1355)	14.6046	3.0280	9850	8655	6737	8407	0.001	0.001	0.001	0.001
p-Ethyltoluene (240)	14.6046	1.2336	60034	59367	76619	70530	0.006	0.006	0.008	0.007
(1171)	14.6213	2.8131	13027	12715	9537	10648	0.001	0.001	0.001	0.001
(1477)	14.6546	0.1776	24268	22892	17037	21025	0.002	0.002	0.002	0.002
Heptanol* (298)	14.7546	1.8598	59265	70591	57200	49337	0.006	0.007	0.006	0.005
(143)	14.7546	2.0374	109859	141270	406104	83733	0.011	0.014	0.041	0.008
m-Ethyltoluene (527)	14.8879	1.2523	28588	27231	22914	27245	0.003	0.003	0.002	0.003
(1197)	14.9546	3.9065	8735	9252	3478	5506	0.001	0.001	0.000	0.001
(1463)	14.9546	0.5514	11944	12231	7517	9683	0.001	0.001	0.001	0.001
Hexanoic acid* (116)	14.9713	3.1776	557248	593497	155220	231392	0.055	0.059	0.016	0.023
(1194)	14.9713	0.2056	17388	17113	8025	12105	0.002	0.002	0.001	0.001
(938)	15.0213	2.7103	13796	12660	9808	12484	0.001	0.001	0.001	0.001
(1062)	15.0213	2.9626	15169	17192	11715	9520	0.001	0.002	0.001	0.001

Compound Name	RT 1D (min)	RT 2D (s)	Volume				Percent Response			
			HM_1	HM_2	LM_1	LM_2	HM_1	HM_2	LM_1	LM_2
3,5,5-Trimethyl-2-hexene (14)	15.0379	1.8224	1274529	1228935	313621	1371267	0.126	0.123	0.032	0.138
(641)	15.0379	0.8598	38677	36887	27175	32817	0.004	0.004	0.003	0.003
(324)	15.0713	1.5794	43841	34137	37267	36103	0.004	0.003	0.004	0.004
487 (331)	15.1213	1.1402	38559	37422	28419	35738	0.004	0.004	0.003	0.004
(602)	15.1879	3.0374	20263	19047	16833	16759	0.002	0.002	0.002	0.002
(1164)	15.2213	3.9626	4579	4637	4226	4203	0.000	0.000	0.000	0.000
(615)	15.2379	3.1495	32810	32644	26057	27422	0.003	0.003	0.003	0.003
1-Octen-3-ol* (6)	15.2713	1.7944	3125117	3070566	2298677	3974897	0.309	0.307	0.234	0.401
(383)	15.2713	2.5327	33439	36763	38745	39306	0.003	0.004	0.004	0.004
(414)	15.2713	2.6916	30811	35703	32855	37516	0.003	0.004	0.003	0.004
(1024)	15.2713	3.5421	13757	13599	11491	12013	0.001	0.001	0.001	0.001
(1072)	15.2879	3.8411	18875	18038	16549	16989	0.002	0.002	0.002	0.002
(1185)	15.3379	3.6262	8589	6646	7751	6475	0.001	0.001	0.001	0.001
Phenol (625)	15.3379	0.4579	83043	92402	36066	20102	0.008	0.009	0.004	0.002
β-Pinene* (228)	15.3546	1.0467	49700	48662	45018	50580	0.005	0.005	0.005	0.005
(287)	15.3546	0.6355	121928	87854	35209	59345	0.012	0.009	0.004	0.006
2,3-Octanedione (51)	15.4213	1.7009	281718	325961	3162768	364169	0.028	0.033	0.322	0.037
(519)	15.4379	1.3925	25162	23293	31152	25934	0.002	0.002	0.003	0.003
(1249)	15.4379	2.9533	9115	8563	9533	8023	0.001	0.001	0.001	0.001
(226)	15.4546	1.9159	41739	44104	35264	38684	0.004	0.004	0.004	0.004
cis-2,6-Dimethyl-2,6-octadiene (246)	15.6379	1.0093	92685	87588	49149	51608	0.009	0.009	0.005	0.005
(1201)	15.6379	2.8224	21005	19416	13762	13846	0.002	0.002	0.001	0.001
101 (890)	15.6713	2.5140	27659	25791	17516	16876	0.003	0.003	0.002	0.002
6-Methyl-5-hepten-2-one* (17)	15.6713	1.7664	1067179	975682	982356	1022287	0.105	0.098	0.100	0.103
(1347)	15.7046	3.5607	57592	60577	16913	14639	0.006	0.006	0.002	0.001
(1393)	15.7213	0.1963	25378	24354	18308	16200	0.003	0.002	0.002	0.002
(1410)	15.8046	2.7570	15537	14352	11289	11233	0.002	0.001	0.001	0.001
2-Octanone* (118)	15.8213	1.7290	201446	225558	633870	218621	0.020	0.023	0.065	0.022
(1520)	15.8546	3.1121	12096	12024	10195	10039	0.001	0.001	0.001	0.001
(1698)	15.8546	0.0748	5229	5070	3421	3750	0.001	0.001	0.000	0.000
β-Mircene* (50)	15.8713	1.0841	424244	390757	237819	258787	0.042	0.039	0.024	0.026
(958)	15.8879	2.2991	30441	29487	22466	23284	0.003	0.003	0.002	0.002
2-Pentylfuran* (12)	15.9213	1.2150	1892020	1932581	1297366	1356306	0.187	0.193	0.132	0.137
1,2,4-Trimethylbenzene (23)	16.0713	1.3738	228724	220643	851652	740006	0.023	0.022	0.087	0.075
(178)	16.0713	0.7664	103171	78163	49005	73865	0.010	0.008	0.005	0.007
(E,Z) 2,4-Heptadienal (175)	16.1379	2.2523	93599	62322	104573	103890	0.009	0.006	0.011	0.010
(1695)	16.1379	3.0374	9163	8834	8618	8681	0.001	0.001	0.001	0.001
(1763)	16.1879	3.4860	7616	6036	5676	5630	0.001	0.001	0.001	0.001
(296)	16.2046	2.1215	24436	22703	33337	36636	0.002	0.002	0.003	0.004
Decane, 2,5,9-trimethyl- (197)	16.2546	0.8972	109439	89932	85740	76232	0.011	0.009	0.009	0.008
(213)	16.3046	1.9439	31383	32958	29194	53837	0.003	0.003	0.003	0.005

			Volume				Percent Response			
**Compound Name**	**RT 1D (min)**	**RT 2D (s)**	**HM_1**	**HM_2**	**LM_1**	**LM_2**	**HM_1**	**HM_2**	**LM_1**	**LM_2**
(Z)-2,6-Dimethyl-2,6-octadiene (217)	16.3379	1.0000	84107	99264	88918	63889	0.008	0.010	0.009	0.006
(1470)	16.3546	3.8972	5549	5510	3790	4909	0.001	0.001	0.000	0.000
(638)	16.3713	0.7850	29227	24002	54208	21239	0.003	0.002	0.006	0.002
trans-2-(2-Pentenyl)furan (59)	16.3713	1.2804	307493	292089	198512	248767	0.030	0.029	0.020	0.025
Octanal* (139)	16.4046	1.6729	130038	117494	117092	113393	0.013	0.012	0.012	0.011
2-(2-Ethoxyethoxy)-ethanol (124)	16.4213	2.2150	151016	160974	139890	183490	0.015	0.016	0.014	0.018
(207)	16.4879	1.9626	63785	50077	63974	76272	0.006	0.005	0.007	0.008
(1175)	16.4879	3.1028	14729	14033	9941	12601	0.001	0.001	0.001	0.001
(1406)	16.5213	3.4393	35067	40285	18431	21313	0.003	0.004	0.002	0.002
α-Phellandrene* (190)	16.5546	1.1589	128525	130201	206649	98420	0.013	0.013	0.021	0.010
(752)	16.5546	0.7196	28378	25490	25702	21264	0.003	0.003	0.003	0.002
(985)	16.6879	2.6542	24224	19956	15238	19153	0.002	0.002	0.002	0.002
cis-Dehydroxy-linalool oxide (149)	16.7379	1.3271	164484	213158	79988	88765	0.016	0.021	0.008	0.009
(906)	16.7879	0.7009	14486	13234	13578	11586	0.001	0.001	0.001	0.001
(E,E) 2,4-Heptadienal* (82)	16.8046	2.2991	378385	280209	347488	352152	0.037	0.028	0.035	0.035
(998)	16.8213	1.6822	8988	7670	7395	8081	0.001	0.001	0.001	0.001
δ-3-Carene (291)	16.8546	1.0841	43066	40905	66745	44481	0.004	0.004	0.007	0.004
(765)	16.8879	0.9346	19241	18951	14872	16997	0.002	0.002	0.002	0.002
(1562)	16.9213	3.5794	6873	6881	5332	5961	0.001	0.001	0.001	0.001
(1337)	16.9379	3.0093	10597	10999	10548	10636	0.001	0.001	0.001	0.001
488 (83)	16.9546	2.0467	173224	156594	182858	229297	0.017	0.016	0.019	0.023
(784)	16.9546	0.6822	19105	18719	18138	16572	0.002	0.002	0.002	0.002
(1248)	16.9546	2.8131	7773	7222	7058	7716	0.001	0.001	0.001	0.001
(1559)	16.9546	3.2056	10093	9067	7899	9310	0.001	0.001	0.001	0.001
3-Hexenol acetate* (648)	16.9713	1.4393	36274	36654	25262	25336	0.004	0.004	0.003	0.003
(1525)	16.9879	3.7196	8126	8084	7028	7300	0.001	0.001	0.001	0.001
(1564)	17.0046	3.4206	9060	8249	8449	8442	0.001	0.001	0.001	0.001
(793)	17.0379	1.5888	18324	15458	18383	16344	0.002	0.002	0.002	0.002
(781)	17.0713	1.7196	22415	20825	13278	15158	0.002	0.002	0.001	0.002
(1766)	17.1713	2.9907	5398	4978	5329	5005	0.001	0.000	0.001	0.001
α-Terpinene* (80)	17.2046	1.1589	218598	216328	168780	165647	0.022	0.022	0.017	0.017
(763)	17.2213	2.3738	9913	9108	11128	11313	0.001	0.001	0.001	0.001
(886)	17.2546	0.6729	27228	26820	27690	24147	0.003	0.003	0.003	0.002
(1048)	17.3046	3.2523	22040	22702	15888	21634	0.002	0.002	0.002	0.002
102 (673)	17.3713	2.6636	36512	30499	20006	26880	0.004	0.003	0.002	0.003
(466)	17.4046	0.8879	44662	40818	32625	35791	0.004	0.004	0.003	0.004
(1073)	17.5379	2.5514	18048	16558	30465	17136	0.002	0.002	0.003	0.002
p-Cymene* (38)	17.5713	1.3271	384262	350859	111218	441196	0.038	0.035	0.011	0.044
2-Ethylhexanol* (54)	17.6046	1.8692	376824	320868	230528	338333	0.037	0.032	0.023	0.034
(558)	17.6879	0.9252	29555	24054	25725	23908	0.003	0.002	0.003	0.002
(1454)	17.7379	2.8411	25756	10184	8762	9511	0.003	0.001	0.001	0.001

Compound Name	RT 1D (min)	RT 2D (s)	Volume				Percent Response			
			HM_1	HM_2	LM_1	LM_2	HM_1	HM_2	LM_1	LM_2
(556)	17.7546	1.8318	29731	26964	131581	24429	0.003	0.003	0.013	0.002
(1098)	17.7546	2.4299	9531	10322	15788	10889	0.001	0.001	0.002	0.001
(657)	17.7713	2.1682	14251	14147	10834	13068	0.001	0.001	0.001	0.001
Limonene* (7)	17.7879	1.1308	3102886	2866352	3345589	2956642	0.306	0.287	0.341	0.298
(1567)	17.8713	3.7103	11428	11120	6978	8361	0.001	0.001	0.001	0.001
(434)	17.8879	2.0000	14854	22719	25000	26692	0.001	0.002	0.003	0.003
(1428)	17.9213	3.4299	11045	12552	5612	6824	0.001	0.001	0.001	0.001
Benzyl alcohol* (158)	17.9379	2.9252	438691	561153	125611	132972	0.043	0.056	0.013	0.013
Eucalyptol* (237)	17.9879	1.3084	15237	15608	53638	29447	0.002	0.002	0.005	0.003
(394)	18.0046	0.9533	39131	34188	27517	26808	0.004	0.003	0.003	0.003
Cyclohexanone, 2,2,6-trimethyl* (25)	18.0713	1.7664	420973	379533	754256	713721	0.042	0.038	0.077	0.072
(Z)-β-Ocimene (87)	18.1046	1.1308	263498	272124	218753	149828	0.026	0.027	0.022	0.015
(1281)	18.1213	3.5234	9858	11387	7055	9527	0.001	0.001	0.001	0.001
(953)	18.1213	2.4112	26333	23723	24052	21686	0.003	0.002	0.002	0.002
5-Ethyl-2(5H)-furanone (502)	18.1379	3.8131	36684	34242	23588	25586	0.004	0.003	0.002	0.003
3-Octen-2-one* (94)	18.1546	2.0467	238580	231779	212404	202933	0.024	0.023	0.022	0.020
Indane (375)	18.1713	1.5047	29101	26297	30738	28795	0.003	0.003	0.003	0.003
1,2,3-Trimethylbenzene (669)	18.2046	1.3364	16361	17256	17327	14613	0.002	0.002	0.002	0.001
(1188)	18.2546	0.0654	22443	19422	15738	17003	0.002	0.002	0.002	0.002
(974)	18.3046	1.7290	11259	9747	46611	11707	0.001	0.001	0.005	0.001
(1078)	18.3046	1.0748	11301	13403	13147	11173	0.001	0.001	0.001	0.001
(1131)	18.3379	3.4206	21494	21396	16157	17937	0.002	0.002	0.002	0.002
(932)	18.4379	2.7664	36290	34750	26220	27451	0.004	0.003	0.003	0.003
(1102)	18.4713	1.0187	15912	15603	11907	11834	0.002	0.002	0.001	0.001
Phenyl acetaldehyde* (72)	18.4879	2.3738	307476	275271	265682	282633	0.030	0.028	0.027	0.028
(1537)	18.4879	3.7944	6171	5874	5118	5994	0.001	0.001	0.001	0.001
(1489)	18.5213	3.6168	7019	6237	5459	6053	0.001	0.001	0.001	0.001
(719)	18.5379	2.0187	34115	35808	34402	30811	0.003	0.004	0.004	0.003
(1597)	18.5546	0.1589	12210	11863	10248	10870	0.001	0.001	0.001	0.001
Indene (568)	18.5879	1.7383	24911	24354	14084	18217	0.002	0.002	0.001	0.002
(E)-β-Ocimene* (57)	18.6213	1.1589	1003048	1195485	266490	298959	0.099	0.120	0.027	0.030
Isobutyl angelate (192)	18.7713	1.4206	63374	63876	88498	79503	0.006	0.006	0.009	0.008
(646)	18.7713	1.0841	606	536	15759	506	0.000	0.000	0.002	0.000
1-Ethyl-1H-pyrrole-2-carboxyaldehyde (53)	18.7879	2.3084	626057	502516	382024	309029	0.062	0.050	0.039	0.031
(946)	18.8046	3.7664	19305	17472	9447	16618	0.002	0.002	0.001	0.002
(1476)	18.8046	2.9907	10576	10014	8890	8081	0.001	0.001	0.001	0.001
(544)	18.8713	1.9252	36080	31276	37645	33810	0.004	0.003	0.004	0.003
(490)	18.8879	0.9907	63770	57929	43489	39294	0.006	0.006	0.004	0.004
(666)	18.8879	1.7383	24516	23720	26012	21167	0.002	0.002	0.003	0.002
(892)	18.9546	3.7196	50188	46069	22641	19338	0.005	0.005	0.002	0.002
(518)	19.0713	0.9346	31431	28918	27871	26759	0.003	0.003	0.003	0.003

Compound Name	RT 1D (min)	RT 2D (s)	Volume HM_1	HM_2	LM_1	LM_2	Percent Response HM_1	HM_2	LM_1	LM_2
(E)-2-Octenal* (170)	19.1046	2.0841	114833	98861	98528	106383	0.011	0.010	0.010	0.011
(1637)	19.1213	2.7850	7097	6821	5683	6403	0.001	0.001	0.001	0.001
(177)	19.1879	1.3925	32959	29888	31641	41314	0.003	0.003	0.003	0.004
2-Methylbenzenemethanol* (1139)	19.2046	2.5421	31953	34549	19337	21975	0.003	0.003	0.002	0.002
(1517)	19.2046	3.2617	16337	16381	13115	13921	0.002	0.002	0.001	0.001
γ-Terpinene* (30)	19.2379	1.1869	465637	411232	550274	532618	0.046	0.041	0.056	0.054
17 (97)	19.3046	1.8224	125227	117873	194430	153837	0.012	0.012	0.020	0.016
(326)	19.3379	0.9907	61739	50361	39988	37104	0.006	0.005	0.004	0.004
(416)	19.4713	0.9813	29407	28907	34668	23861	0.003	0.003	0.004	0.002
(500)	19.4713	1.4953	18845	17205	20237	24892	0.002	0.002	0.002	0.003
(E)-2-Octenol (10)	19.5046	2.0374	1019337	916165	1188894	1419509	0.101	0.092	0.121	0.143
(607)	19.5213	2.7944	13876	12680	8336	13605	0.001	0.001	0.001	0.001
(926)	19.5546	3.4953	11490	11288	6769	9385	0.001	0.001	0.001	0.001
(1108)	19.5546	3.8505	9176	8662	6339	8869	0.001	0.001	0.001	0.001
(1361)	19.5713	0.7290	5883	5966	5268	6700	0.001	0.001	0.001	0.001
(1170)	19.6046	0.0654	11232	9013	6857	9551	0.001	0.001	0.001	0.001
1-Octanol* (37)	19.6046	1.9346	550372	704725	907470	696394	0.054	0.071	0.092	0.070
Heptanoic acid* (351)	19.6046	3.1869	172014	159131	75068	99719	0.017	0.016	0.008	0.010
(1154)	19.6213	0.3925	16705	14939	12805	16079	0.002	0.001	0.001	0.002
(370)	19.6546	0.9439	49160	41818	38687	34717	0.005	0.004	0.004	0.003
(747)	19.6546	1.4486	23880	23472	26331	22978	0.002	0.002	0.003	0.002
491 (21)	19.7046	2.1589	1399944	1415399	1485087	1515406	0.138	0.142	0.151	0.153
(233)	19.7213	3.4953	278196	114924	122281	102571	0.027	0.012	0.012	0.010
(448)	19.8213	1.8692	47926	83979	63590	31229	0.005	0.008	0.006	0.003
Acetophenone (879)	19.8713	2.5981	26066	30385	20969	20542	0.003	0.003	0.002	0.002
(514)	19.8713	0.0654	79083	73963	61353	68447	0.008	0.007	0.006	0.007
cis-Linalool oxide (furanoid)* (198)	19.9046	1.6729	1506253	1609721	78980	68554	0.149	0.161	0.008	0.007
(428)	19.9713	1.2897	24578	21472	21394	27039	0.002	0.002	0.002	0.003
(1345)	20.0046	2.8785	20002	22146	17931	17762	0.002	0.002	0.002	0.002
(1726)	20.0213	3.5981	5912	6353	5020	5117	0.001	0.001	0.001	0.001
(1421)	20.0546	3.3738	9648	10240	9018	9713	0.001	0.001	0.001	0.001
(1664)	20.0713	3.2336	7469	6319	5386	5888	0.001	0.001	0.001	0.001
494 (96)	20.0879	1.0374	292271	263967	165890	238350	0.029	0.026	0.017	0.024
(808)	20.0879	1.6075	18347	18604	12591	11282	0.002	0.002	0.001	0.001
(1047)	20.0879	0.0374	10868	9117	9047	8619	0.001	0.001	0.001	0.001
490 (303)	20.2213	0.9907	97893	83813	127596	55919	0.010	0.008	0.013	0.006
(532)	20.2213	1.4206	17711	19290	18258	17360	0.002	0.002	0.002	0.002
(815)	20.2379	1.8411	20613	18973	21976	19518	0.002	0.002	0.002	0.002
(1600)	20.2546	2.9439	11549	10904	9262	10896	0.001	0.001	0.001	0.001
(1723)	20.2713	0.3925	5704	5827	4916	5706	0.001	0.001	0.001	0.001
123 (460)	20.2879	2.0467	47780	45233	61956	47519	0.005	0.005	0.006	0.005

Compound Name	RT 1D (min)	RT 2D (s)	Volume HM_1	HM_2	LM_1	LM_2	Percent Response HM_1	HM_2	LM_1	LM_2
(1326)	20.2879	3.8505	10497	10711	10526	9328	0.001	0.001	0.001	0.001
(288)	20.3213	1.2336	32167	27218	35889	36100	0.003	0.003	0.004	0.004
(670)	20.4546	1.8785	31833	28301	19494	20669	0.003	0.003	0.002	0.002
(436)	20.5213	1.4393	31579	24100	19263	24637	0.003	0.002	0.002	0.002
o-Methoxyphenol (783)	20.5213	2.4393	20314	18231	12329	15576	0.002	0.002	0.001	0.002
(791)	20.5379	2.1402	62579	81677	19311	19266	0.006	0.008	0.002	0.002
(1301)	20.5379	3.8411	9093	10069	7270	8573	0.001	0.001	0.001	0.001
(1370)	20.6046	3.6262	14093	14405	10321	10023	0.001	0.001	0.001	0.001
3,3,5-Trimethyl-1,4-hexadiene (241)	20.6213	1.0000	215605	185184	112731	115267	0.021	0.019	0.011	0.012
(1544)	20.6546	2.8785	19039	19023	6388	6910	0.002	0.002	0.001	0.001
trans-Linalool oxide (furanoid)* (129)	20.6879	1.7664	4024784	4856390	136225	167253	0.397	0.486	0.014	0.017
Terpinolene (49)	20.7046	1.1869	407970	399949	288568	273432	0.040	0.040	0.029	0.028
p-Cymenene (218)	20.7046	1.5421	711059	114239	67620	74641	0.070	0.011	0.007	0.008
(Z,E)-3,5-octadien-2-one* (191)	20.8379	2.2991	216803	243525	117105	121576	0.021	0.024	0.012	0.012
(273)	20.8546	0.9813	109761	81401	57297	47983	0.011	0.008	0.006	0.005
2-Nonanone* (420)	21.0213	1.3551	64281	66237	54970	49572	0.006	0.007	0.006	0.005
4,7-Dimethylundecane (86)	21.0879	0.9626	382889	316165	150265	196269	0.038	0.032	0.015	0.020
Linalool* (15)	21.1213	1.8037	11207481	11982831	945937	1109491	1.106	1.200	0.096	0.112
(462)	21.1213	2.2056	145110	172144	31623	31678	0.014	0.017	0.003	0.003
(778)	21.1213	2.4766	85197	99545	18759	20221	0.008	0.010	0.002	0.002
(1340)	21.1213	2.8879	32432	35528	10251	10612	0.003	0.004	0.001	0.001
(1654)	21.1213	3.2523	26118	29155	10449	10227	0.003	0.003	0.001	0.001
(1740)	21.2046	0.3925	9432	10150	6485	6305	0.001	0.001	0.001	0.001
(1703)	21.2046	0.0748	7239	6922	4129	3815	0.001	0.001	0.000	0.000
(267)	21.2546	0.9813	144592	124380	105087	70512	0.014	0.012	0.011	0.007
(649)	21.2713	1.4112	23660	22760	18446	18402	0.002	0.002	0.002	0.002
Nonanal* (48)	21.3213	1.7477	656435	657406	672197	331507	0.065	0.066	0.068	0.033
Hotrienol* (28)	21.3713	1.9439	1618234	1838281	456214	579394	0.160	0.184	0.046	0.058
(1292)	21.3713	3.0280	12771	12957	8524	8552	0.001	0.001	0.001	0.001
(1325)	21.3713	3.3738	18429	19334	14469	14866	0.002	0.002	0.001	0.001
(1502)	21.3713	3.1963	9753	11360	7050	7422	0.001	0.001	0.001	0.001
6-Methyl-3,5-heptadien-2-one* (159)	21.3879	2.3738	150378	152871	92285	114821	0.015	0.015	0.009	0.012
(868)	21.3879	2.7664	21143	21854	13012	15426	0.002	0.002	0.001	0.002
(318)	21.4546	1.0000	103598	88677	65535	59275	0.010	0.009	0.007	0.006
1,3,8-p-Menthatriene (206)	21.5046	1.5327	80178	66451	77103	61991	0.008	0.007	0.008	0.006
(1046)	21.5046	3.8131	18880	16379	12313	11905	0.002	0.002	0.001	0.001
α-Thujone* (20)	21.5879	1.9533	766652	647171	885511	758843	0.076	0.065	0.090	0.076
(563)	21.6713	2.8037	22240	19070	14561	21381	0.002	0.002	0.001	0.002
(1377)	21.6713	3.2523	12640	10704	8476	9796	0.001	0.001	0.001	0.001
105 (106)	21.7046	2.1963	164594	151113	140201	177359	0.016	0.015	0.014	0.018
(160)	21.7213	0.9720	338390	246023	111866	128925	0.033	0.025	0.011	0.013

Compound Name	RT 1D (min)	RT 2D (s)	Volume				Percent Response			
			HM_1	HM_2	LM_1	LM_2	HM_1	HM_2	LM_1	LM_2
(1335)	21.7879	3.7570	27020	22903	13403	13291	0.003	0.002	0.001	0.001
495 (520)	21.8546	1.6168	63221	62760	36246	35653	0.006	0.006	0.004	0.004
2-Phenylethanol* (270)	21.8546	2.7664	669653	321651	35619	47832	0.066	0.032	0.004	0.005
(1399)	21.8546	3.1589	20765	18885	8198	8125	0.002	0.002	0.001	0.001
(1561)	21.8546	3.3178	13553	13967	5956	5535	0.001	0.001	0.001	0.001
(E,E)-Octadienal* (333)	21.8713	2.6916	279585	609303	65168	42345	0.028	0.061	0.007	0.004
114 (545)	21.8879	2.1869	42700	48201	31760	31340	0.004	0.005	0.003	0.003
497 (128)	21.9213	0.9813	422062	358035	183914	169721	0.042	0.036	0.019	0.017
498 (145)	21.9379	1.2430	257744	277920	110061	97292	0.025	0.028	0.011	0.010
(1760)	22.0213	0.1402	8883	8918	6743	6907	0.001	0.001	0.001	0.001
(552)	22.0546	1.4953	26114	28206	20787	21773	0.003	0.003	0.002	0.002
(243)	22.0879	1.0000	92808	85618	58062	48644	0.009	0.009	0.006	0.005
β-Thujone* (117)	22.1046	1.9720	121693	104720	128588	116744	0.012	0.010	0.013	0.012
(1431)	22.1046	2.9252	9183	10176	6664	8219	0.001	0.001	0.001	0.001
(229)	22.1713	1.8318	38436	38404	52722	42177	0.004	0.004	0.005	0.004
2-Ethyl-m-xylene (405)	22.2546	1.4953	63584	62984	49840	43867	0.006	0.006	0.005	0.004
(1234)	22.2713	2.6636	13200	12332	9638	9605	0.001	0.001	0.001	0.001
(608)	22.3213	1.8505	18470	19993	13140	15624	0.002	0.002	0.001	0.002
(671)	22.4546	1.6355	30127	28993	19245	17816	0.003	0.003	0.002	0.002
(1349)	22.4546	2.5794	13814	14651	10938	10149	0.001	0.001	0.001	0.001
500 (134)	22.4546	1.0000	460733	376108	178015	173144	0.045	0.038	0.018	0.017
allo-Ocimene (366)	22.6046	1.3645	83017	62999	33267	35426	0.008	0.006	0.003	0.004
501 (381)	22.6879	1.4766	58403	94385	38313	35174	0.006	0.009	0.004	0.004
(531)	22.7046	1.8879	43952	46827	26330	27143	0.004	0.005	0.003	0.003
502 (219)	22.7213	1.0000	260088	172542	81682	73901	0.026	0.017	0.008	0.007
(978)	22.7713	2.1028	10634	10311	8833	9012	0.001	0.001	0.001	0.001
1-Ethyl-2,5-pyrrolidinedione (456)	22.8546	3.5140	71661	70898	33809	33054	0.007	0.007	0.003	0.003
503 (242)	22.8713	0.9907	171595	148161	67660	65924	0.017	0.015	0.007	0.007
(1436)	22.8713	1.7009	10878	11660	10085	8981	0.001	0.001	0.001	0.001
504 (257)	23.0213	1.0093	141101	126017	66963	53434	0.014	0.013	0.007	0.005
(1026)	23.0213	1.4206	11688	12232	6415	8727	0.001	0.001	0.001	0.001
3-Nonen-2-one (482)	23.0546	2.0841	79890	76411	54512	56257	0.008	0.008	0.006	0.006
Benzylnitrile* (585)	23.1379	3.3271	764360	784509	54572	49342	0.075	0.079	0.006	0.005
(1315)	23.1713	2.3925	8471	7886	7341	7370	0.001	0.001	0.001	0.001
(606)	23.2046	1.5607	23864	25918	18155	19045	0.002	0.003	0.002	0.002
(922)	23.2046	1.3925	24931	24847	12250	11242	0.002	0.002	0.001	0.001
5-Propyldecane (572)	23.2546	0.9813	43749	32046	13742	14907	0.004	0.003	0.001	0.002
505 (467)	23.3379	1.0280	69654	57953	23689	27780	0.007	0.006	0.002	0.003
(933)	23.3546	2.6449	23264	22272	16419	20357	0.002	0.002	0.002	0.002
(1314)	23.3546	3.1682	45088	58965	25979	17260	0.004	0.006	0.003	0.002
(1640)	23.4046	2.4860	6123	7040	5773	5484	0.001	0.001	0.001	0.001

			Volume				Percent Response			
Compound Name	RT 1D (min)	RT 2D (s)	HM_1	HM_2	LM_1	LM_2	HM_1	HM_2	LM_1	LM_2
(R,S)-5-Ethyl-6-methyl-3E-hepten-2-one (260)	23.4213	2.0374	84511	89901	43705	47657	0.008	0.009	0.004	0.005
(95)	23.5546	1.5047	178899	165560	128372	142140	0.018	0.017	0.013	0.014
(655)	23.5713	2.1589	17584	14583	13578	16383	0.002	0.001	0.001	0.002
(1363)	23.6546	3.0935	20360	23921	13758	12830	0.002	0.002	0.001	0.001
3-Nonen-1-ol* (276)	23.6879	1.9346	62103	57853	62576	49929	0.006	0.006	0.006	0.005
2-Ethylhexyl acetate (560)	23.7046	1.4673	18572	15871	32788	15871	0.002	0.002	0.003	0.002
(68)	23.7379	0.9252	695987	559040	406432	388906	0.069	0.056	0.041	0.039
(1157)	23.7546	2.2336	19201	17048	11792	14026	0.002	0.002	0.001	0.001
506 (121)	23.7713	1.0280	436899	321547	119240	131407	0.043	0.032	0.012	0.013
(472)	23.8213	1.5421	42290	42648	31993	31446	0.004	0.004	0.003	0.003
Menthone* (127)	23.9213	2.0093	186972	162087	229747	217051	0.018	0.016	0.023	0.022
(1233)	23.9713	0.4019	20484	20879	17027	19481	0.002	0.002	0.002	0.002
508 (144)	23.9879	0.9813	300792	239028	96965	92245	0.030	0.024	0.010	0.009
(1619)	24.0213	0.3084	6178	5487	5201	5367	0.001	0.001	0.001	0.001
(839)	24.0379	1.4579	21173	18652	13477	13977	0.002	0.002	0.001	0.001
(E)-2-Nonenal* (497)	24.1046	2.3178	15433	15279	12687	17371	0.002	0.002	0.001	0.002
118 (209)	24.1379	2.5794	37991	36349	95319	87910	0.004	0.004	0.010	0.009
(1545)	24.1713	0.2430	7707	7606	6367	7379	0.001	0.001	0.001	0.001
4-Methylundecane (165)	24.1879	0.9907	429607	315889	122713	123846	0.042	0.032	0.012	0.012
(637)	24.3046	2.1028	22969	20578	8435	10444	0.002	0.002	0.001	0.001
(440)	24.3046	1.4673	24868	22700	31508	32384	0.002	0.002	0.003	0.003
Octanoic acid* (81)	24.3379	3.0187	289921	325376	114940	322191	0.029	0.033	0.012	0.032
(779)	24.3379	3.8598	24694	23334	15540	24254	0.002	0.002	0.002	0.002
(912)	24.3379	3.6822	14100	13293	7820	12817	0.001	0.001	0.001	0.001
Menthofuran (392)	24.4046	1.5421	61732	50668	54709	57063	0.006	0.005	0.006	0.006
(1009)	24.4213	0.1308	26639	25430	17781	24702	0.003	0.003	0.002	0.002
(1136)	24.4213	0.4673	19490	19064	14132	19708	0.002	0.002	0.001	0.002
(714)	24.4379	2.2897	22926	19473	14095	15668	0.002	0.002	0.001	0.002
Nonanol* (182)	24.5213	1.9252	141771	125925	72932	102585	0.014	0.013	0.007	0.010
(90)	24.5213	1.0000	531451	422365	165191	167595	0.052	0.042	0.017	0.017
Epoxylinalool* (1195)	24.5546	2.6355	12422	11252	6452	8712	0.001	0.001	0.001	0.001
(597)	24.5879	1.5514	19104	18492	15432	16482	0.002	0.002	0.002	0.002
cis-Linalool oxide (pyranoid) (169)	24.6046	2.2243	269408	345214	64994	75567	0.027	0.035	0.007	0.008
Ethyl benzoate* (41)	24.6879	1.9720	669718	621722	567128	554292	0.066	0.062	0.058	0.056
3-Methylundecane (681)	24.7379	1.0280	61749	48300	26715	29038	0.006	0.005	0.003	0.003
2,4-Dimethylbenzaldehyde (63)	24.7713	2.2617	1627826	1349409	72388	171285	0.161	0.135	0.007	0.017
(848)	24.7879	1.4486	38917	40497	32241	33051	0.004	0.004	0.003	0.003
(944)	24.7879	2.6168	34214	33971	14642	16240	0.003	0.003	0.001	0.002
(1208)	24.7879	2.8692	24328	21790	11349	12851	0.002	0.002	0.001	0.001
(1305)	24.7879	3.1402	18949	19447	11609	13066	0.002	0.002	0.001	0.001
(1687)	24.7879	3.6636	7744	8617	5016	6983	0.001	0.001	0.001	0.001

Compound Name	RT 1D (min)	RT 2D (s)	Volume HM_1	HM_2	LM_1	LM_2	Percent Response HM_1	HM_2	LM_1	LM_2
(1588)	24.7879	3.8598	8916	9218	6169	7107	0.001	0.001	0.001	0.001
(1618)	24.8213	3.5794	7254	8691	5210	5643	0.001	0.001	0.001	0.001
trans-Linalool oxide (pyranoid)* (1789)	24.8379	2.2243	169589	502798	221762	145663	0.017	0.050	0.023	0.015
(634)	24.8713	1.0093	44209	38394	19905	19535	0.004	0.004	0.002	0.002
(1655)	24.9213	0.6542	13832	13793	9576	11736	0.001	0.001	0.001	0.001
(1642)	24.9546	3.2150	6133	6302	4968	5513	0.001	0.001	0.001	0.001
(1774)	24.9713	0.2897	6709	6486	6121	6434	0.001	0.001	0.001	0.001
Terpine-4-ol* (205)	25.0713	1.7850	113088	107459	86044	88326	0.011	0.011	0.009	0.009
(891)	25.0713	1.5701	17150	14801	9781	11512	0.002	0.001	0.001	0.001
(1743)	25.0713	2.9720	7209	7510	5392	6187	0.001	0.001	0.001	0.001
(584)	25.1046	1.0467	60531	46650	19754	21923	0.006	0.005	0.002	0.002
(277)	25.2546	1.0748	196968	161166	70430	74395	0.019	0.016	0.007	0.007
(Z)-3-Hexenyl butyrate* (664)	25.2879	1.5514	246258	267391	13460	14583	0.024	0.027	0.001	0.001
(508)	25.3379	1.7009	29526	33771	20288	22448	0.003	0.003	0.002	0.002
2,6-Dimethyl-3,7-octadiene-2,6-diol (183)	25.4379	2.8972	212902	209675	87407	84770	0.021	0.021	0.009	0.009
511 (342)	25.4546	2.2430	108806	103690	66249	84554	0.011	0.010	0.007	0.009
Naphthalene (102)	25.4546	2.0187	253481	258274	137108	150180	0.025	0.026	0.014	0.015
(1758)	25.4879	0.0935	3663	3531	2470	2721	0.000	0.000	0.000	0.000
(148)	25.5379	1.0748	328988	261366	119632	118420	0.032	0.026	0.012	0.012
Hexyl butyrate* (676)	25.5713	1.5140	95682	65999	16466	19583	0.009	0.007	0.002	0.002
2-Decanone (146)	25.6213	1.8505	50714	51318	64461	61785	0.005	0.005	0.007	0.006
(674)	25.6213	1.3832	24005	25923	16059	14674	0.002	0.003	0.002	0.001
α-Terpineol* (70)	25.6879	2.0000	447871	476152	254511	270953	0.044	0.048	0.026	0.027
(667)	25.6879	2.3458	35670	33453	21291	24628	0.004	0.003	0.002	0.002
(E)-2-Hexenyl butyrate (406)	25.7546	1.5327	73769	98909	38096	38357	0.007	0.010	0.004	0.004
Methyl salicylate* (27)	25.8879	2.1589	5170121	6007809	739293	764344	0.510	0.602	0.075	0.077
(1324)	25.8879	3.3925	19608	20126	7428	9296	0.002	0.002	0.001	0.001
(1395)	25.8879	3.4860	15765	16897	6629	6963	0.002	0.002	0.001	0.001
(1732)	25.8879	3.9626	4291	4204	2387	2204	0.000	0.000	0.000	0.000
(826)	25.9046	2.8224	32620	36677	9704	10380	0.003	0.004	0.001	0.001
(1033)	25.9046	3.1028	56984	62621	26159	25866	0.006	0.006	0.003	0.003
(1504)	25.9046	3.7944	16949	18595	7412	7337	0.002	0.002	0.001	0.001
Dodecane (36)	25.9213	1.0280	1535009	1204106	561722	583517	0.152	0.121	0.057	0.059
(1629)	25.9546	0.6636	13146	13476	7278	7396	0.001	0.001	0.001	0.001
(1712)	25.9713	0.1308	11678	12226	6039	5779	0.001	0.001	0.001	0.001
(1678)	25.9879	0.4860	11205	11806	6148	6676	0.001	0.001	0.001	0.001
Estragole* (163)	26.0213	1.8037	81472	82343	90050	76273	0.008	0.008	0.009	0.008
(549)	26.0379	1.6075	42080	44740	32080	30726	0.004	0.004	0.003	0.003
(1708)	26.0879	3.4019	8634	10027	5623	6170	0.001	0.001	0.001	0.001
(642)	26.1379	2.4393	18191	22564	13001	12765	0.002	0.002	0.001	0.001
(1547)	26.1379	3.6075	15355	16681	10192	11728	0.002	0.002	0.001	0.001

Compound Name	RT 1D (min)	RT 2D (s)	Volume HM_1	HM_2	LM_1	LM_2	Percent Response HM_1	HM_2	LM_1	LM_2
Safranal (62)	26.1546	2.0935	506406	703750	341696	302642	0.050	0.070	0.035	0.031
(905)	26.1546	2.5794	34572	41135	26083	26320	0.003	0.004	0.003	0.003
(1591)	26.2046	0.0467	4634	4468	4029	3869	0.000	0.000	0.000	0.000
Decanal* (161)	26.2379	1.8131	139803	106528	98823	74067	0.014	0.011	0.010	0.007
(473)	26.2713	1.0561	188832	151239	74502	74965	0.019	0.015	0.008	0.008
(1013)	26.5046	1.5234	29664	28799	18359	17597	0.003	0.003	0.002	0.002
(1243)	26.5046	2.4112	8336	9435	6171	7637	0.001	0.001	0.001	0.001
(265)	26.5546	1.9907	69306	62671	54753	55962	0.007	0.006	0.006	0.006
513 (238)	26.6213	1.0561	325938	238318	108101	109565	0.032	0.024	0.011	0.011
129 (491)	26.6213	2.1402	15720	23049	10406	15680	0.002	0.002	0.001	0.002
(835)	26.6546	2.2991	11202	12324	9915	10169	0.001	0.001	0.001	0.001
(1581)	26.6879	2.6729	8449	8714	6778	7841	0.001	0.001	0.001	0.001
(E,E)-2,4-Nonadienal* (1052)	26.6879	2.4019	10916	13351	9044	10746	0.001	0.001	0.001	0.001
(1405)	26.7213	1.5514	14948	13662	8177	9189	0.001	0.001	0.001	0.001
(492)	26.8379	1.1121	30066	30489	13974	15726	0.003	0.003	0.001	0.002
(1041)	26.9546	2.6168	9640	8792	7168	8846	0.001	0.001	0.001	0.001
131 (46)	26.9713	2.0748	430743	370072	313850	330749	0.043	0.037	0.032	0.033
2-Phenoxyethanol (259)	26.9713	3.0467	85500	76764	57316	101568	0.008	0.008	0.006	0.010
(1444)	26.9713	3.6075	13786	12955	10034	12301	0.001	0.001	0.001	0.001
(1685)	26.9879	3.8598	7197	6165	5273	6204	0.001	0.001	0.001	0.001
(1699)	27.0046	0.5514	8843	8563	7990	7492	0.001	0.001	0.001	0.001
(874)	27.0213	1.6355	25472	26545	18556	20497	0.003	0.003	0.002	0.002
(391)	27.0379	1.1028	154324	120079	52474	58419	0.015	0.012	0.005	0.006
(1245)	27.0546	1.5234	14605	13840	10631	9184	0.001	0.001	0.001	0.001
(1721)	27.1213	0.0654	3139	3275	2560	3006	0.000	0.000	0.000	0.000
β-Cyclocitral* (22)	27.1546	2.0935	535811	491117	678725	652314	0.053	0.049	0.069	0.066
(1660)	27.1879	3.6355	7912	7161	7086	8107	0.001	0.001	0.001	0.001
(488)	27.2213	1.0654	91970	71289	33997	32056	0.009	0.007	0.003	0.003
(566)	27.2379	2.7850	37222	31233	32941	37278	0.004	0.003	0.003	0.004
(618)	27.2546	1.9346	20617	26543	13995	14543	0.002	0.003	0.001	0.001
(1744)	27.2879	0.1682	10201	10291	9366	10106	0.001	0.001	0.001	0.001
Nerol* (404)	27.3546	2.0374	73069	85912	33391	33728	0.007	0.009	0.003	0.003
(883)	27.3546	2.4393	23308	19837	16451	19551	0.002	0.002	0.002	0.002
(580)	27.3713	1.1308	33248	27799	16247	16313	0.003	0.003	0.002	0.002
(901)	27.4379	3.6449	29252	26102	22773	24535	0.003	0.003	0.002	0.002
(Z)-3-Hexenyl isovalerate* (210)	27.4379	1.5327	123115	96096	61058	59932	0.012	0.010	0.006	0.006
(546)	27.5213	1.0654	109553	90039	42750	41727	0.011	0.009	0.004	0.004
Bornylene (220)	27.5379	1.5701	113913	150579	45025	53994	0.011	0.015	0.005	0.005
(1486)	27.5879	2.6916	8579	7180	5603	6482	0.001	0.001	0.001	0.001
(633)	27.7046	1.0561	34620	24357	14293	13933	0.003	0.002	0.001	0.001
(767)	27.7546	1.7290	13627	12407	8714	9559	0.001	0.001	0.001	0.001

			Volume				Percent Response			
**Compound Name**	**RT 1D (min)**	**RT 2D (s)**	**HM_1**	**HM_2**	**LM_1**	**LM_2**	**HM_1**	**HM_2**	**LM_1**	**LM_2**
(E)-2-Hexenyl isovalerate* (114)	27.8213	1.5047	218301	209675	117724	116846	0.022	0.021	0.012	0.012
(988)	27.8213	2.6916	7402	6838	5158	5715	0.001	0.001	0.001	0.001
(872)	27.8713	2.0561	32807	32682	15357	18647	0.003	0.003	0.002	0.002
(1321)	27.9213	3.6262	15130	13505	11283	11592	0.001	0.001	0.001	0.001
(1359)	27.9213	3.7103	8188	8361	5170	6582	0.001	0.001	0.001	0.001
(528)	27.9379	1.9346	26169	26708	16250	19012	0.003	0.003	0.002	0.002
(1180)	27.9879	3.9626	1671	1373	988	1672	0.000	0.000	0.000	0.000
516 (120)	28.0046	1.1215	443833	347816	154310	156682	0.044	0.035	0.016	0.016
132 (75)	28.0213	2.5888	201287	192329	181775	185809	0.020	0.019	0.019	0.019
(425)	28.0213	2.9252	48794	44156	40556	42008	0.005	0.004	0.004	0.004
(1183)	28.0213	3.4486	10949	11194	9160	8924	0.001	0.001	0.001	0.001
(1415)	28.0213	3.8692	10506	9947	8620	9577	0.001	0.001	0.001	0.001
(1574)	28.1046	0.3925	9667	7959	15756	7788	0.001	0.001	0.002	0.001
518 (309)	28.1713	1.0935	147222	112469	54014	55060	0.015	0.011	0.006	0.006
(1319)	28.1713	3.5794	11118	10936	9405	9483	0.001	0.001	0.001	0.001
(494)	28.2213	2.2336	31921	25888	22380	23238	0.003	0.003	0.002	0.002
(610)	28.2213	1.8224	24801	21806	20661	24331	0.002	0.002	0.002	0.002
(1061)	28.2213	3.4206	13328	9519	10224	12845	0.001	0.001	0.001	0.001
(1109)	28.2546	2.5327	20771	19958	13800	15101	0.002	0.002	0.001	0.002
(1475)	28.2713	2.8411	9847	9091	8264	9357	0.001	0.001	0.001	0.001
(1620)	28.2879	0.1589	5135	4885	5022	5356	0.001	0.000	0.001	0.001
(806)	28.3046	1.5140	24640	19319	18060	16768	0.002	0.002	0.002	0.002
(525)	28.4213	1.0280	100896	76543	41965	41363	0.010	0.008	0.004	0.004
Carvone (465)	28.4713	2.2523	64986	64235	31783	35187	0.006	0.006	0.003	0.004
Geraniol* (312)	28.5213	2.0561	507123	537842	67925	73205	0.050	0.054	0.007	0.007
(1240)	28.5546	2.5234	24327	30652	13181	15414	0.002	0.003	0.001	0.002
Linalyl acetate (255)	28.6213	1.5888	58209	53826	48393	52925	0.006	0.005	0.005	0.005
(341)	28.7379	1.0841	206739	154507	65448	67013	0.020	0.015	0.007	0.007
(1253)	28.7713	2.8505	29736	29014	14030	13852	0.003	0.003	0.001	0.001
(1216)	28.8213	3.5701	13649	12463	9456	10519	0.001	0.001	0.001	0.001
Phenylethyl acetate (227)	28.8879	2.1589	71377	66670	44681	67538	0.007	0.007	0.005	0.007
(530)	28.8879	1.4766	51097	47153	28470	29297	0.005	0.005	0.003	0.003
Homocyclocitral (315)	28.9046	2.0187	56061	52251	61030	39169	0.006	0.005	0.006	0.004
(1312)	28.9046	2.6075	10262	9657	8070	8494	0.001	0.001	0.001	0.001
519 (266)	28.9213	1.0374	291502	235183	103571	115564	0.029	0.024	0.011	0.012
Nonanoic acid* (123)	28.9546	2.8692	270526	247719	106367	169736	0.027	0.025	0.011	0.017
(1132)	28.9546	3.7850	18251	15005	11202	13272	0.002	0.002	0.001	0.001
(1082)	28.9713	3.5794	14922	13360	8320	11541	0.001	0.001	0.001	0.001
(1364)	28.9713	3.9626	2951	2848	2008	2380	0.000	0.000	0.000	0.000
(1381)	29.0213	0.5234	19508	18216	12723	14859	0.002	0.002	0.001	0.001
(1351)	29.0379	0.0935	22925	21630	15198	18722	0.002	0.002	0.002	0.002

			Volume				Percent Response			
**Compound Name**	**RT 1D (min)**	**RT 2D (s)**	**HM_1**	**HM_2**	**LM_1**	**LM_2**	**HM_1**	**HM_2**	**LM_1**	**LM_2**
(594)	29.1046	2.5234	32272	29135	23172	27096	0.003	0.003	0.002	0.003
(487)	29.2379	1.0467	53429	40935	19763	22083	0.005	0.004	0.002	0.002
(992)	29.2713	1.4112	30403	28898	20946	21949	0.003	0.003	0.002	0.002
(738)	29.3046	1.9346	26841	28223	12729	16891	0.003	0.003	0.001	0.002
(1605)	29.3046	0.6075	10851	10054	8188	9349	0.001	0.001	0.001	0.001
(541)	29.3379	1.7570	27785	28486	14910	17259	0.003	0.003	0.002	0.002
(617)	29.3379	1.7103	38288	32499	25162	22887	0.004	0.003	0.003	0.002
Citral* (980)	29.3546	2.2804	50922	51579	28978	31334	0.005	0.005	0.003	0.003
(348)	29.3546	1.0280	160298	122326	55689	61094	0.016	0.012	0.006	0.006
(1400)	29.3713	2.7383	14725	11188	9156	9529	0.001	0.001	0.001	0.001
(1481)	29.3879	2.9907	19686	21602	9161	9532	0.002	0.002	0.001	0.001
(1684)	29.3879	3.4393	7614	7266	5854	6372	0.001	0.001	0.001	0.001
(1615)	29.5546	2.6916	11633	7475	5701	6725	0.001	0.001	0.001	0.001
(1111)	29.5879	1.9252	35647	36596	18520	20046	0.004	0.004	0.002	0.002
(991)	29.6546	1.4393	22941	20680	16722	16756	0.002	0.002	0.002	0.002
(493)	29.7213	3.0561	36394	31372	28198	31633	0.004	0.003	0.003	0.003
(1420)	29.7879	2.3645	11724	11847	8498	8655	0.001	0.001	0.001	0.001
(1466)	29.8046	2.6168	13358	11605	11069	11952	0.001	0.001	0.001	0.001
(283)	29.8379	1.0748	228194	171034	75256	81150	0.023	0.017	0.008	0.008
520 (254)	29.9546	1.1121	127304	111100	43704	49272	0.013	0.011	0.004	0.005
(1446)	29.9546	2.8224	9535	8372	8536	8355	0.001	0.001	0.001	0.001
Pentyl hexanoate (574)	30.0879	1.5047	57451	54142	26677	29902	0.006	0.005	0.003	0.003
(374)	30.1546	1.9813	58998	51863	46367	43129	0.006	0.005	0.005	0.004
(1279)	30.1546	2.4486	28403	28356	19457	21849	0.003	0.003	0.002	0.002
(1773)	30.1713	3.9346	4337	3702	3248	3738	0.000	0.000	0.000	0.000
Dihydrocitronellol (35)	30.2379	1.1121	1212350	964306	470750	527000	0.120	0.097	0.048	0.053
2-Undecanone (221)	30.3546	1.8505	114579	109049	92174	94307	0.011	0.011	0.009	0.010
(1450)	30.4046	2.2991	10054	9154	8052	7392	0.001	0.001	0.001	0.001
(1003)	30.5213	3.2710	22120	19385	16617	18614	0.002	0.002	0.002	0.002
(481)	30.5546	1.7383	45390	44486	34636	35300	0.004	0.004	0.004	0.004
(84)	30.5713	3.9533	162283	160721	578982	179334	0.016	0.016	0.059	0.018
Tridecane (56)	30.5879	1.0467	933964	737967	415872	436169	0.092	0.074	0.042	0.044
(1065)	30.5879	0.4206	27155	23951	28552	22851	0.003	0.002	0.003	0.002
(1458)	30.5879	2.5607	8343	8201	7484	7943	0.001	0.001	0.001	0.001
2-Methylnaphthalene (180)	30.6379	2.0654	129363	126217	82612	81983	0.013	0.013	0.008	0.008
(924)	30.6713	1.5888	31618	28796	22579	21506	0.003	0.003	0.002	0.002
(1261)	30.6713	0.6542	14374	12989	15326	12548	0.001	0.001	0.002	0.001
1-Nitro-2-phenylethane (1092)	30.7879	3.2710	43529	43761	23379	19835	0.004	0.004	0.002	0.002
(1356)	30.8046	2.9813	16710	13996	14540	14345	0.002	0.001	0.001	0.001
(550)	30.8379	2.2430	22236	24326	19607	20940	0.002	0.002	0.002	0.002
(1252)	30.8546	2.7757	11689	12108	9718	10388	0.001	0.001	0.001	0.001

Compound Name	RT 1D (min)	RT 2D (s)	Volume				Percent Response			
			HM_1	HM_2	LM_1	LM_2	HM_1	HM_2	LM_1	LM_2
Theaspirane A* (264)	30.9046	1.4673	62655	60865	37089	39877	0.006	0.006	0.004	0.004
522 (154)	30.9546	1.1776	189434	158031	65006	80859	0.019	0.016	0.007	0.008
(1060)	30.9879	3.7570	13071	12206	13189	16803	0.001	0.001	0.001	0.002
(200)	31.0046	1.1121	179167	120406	69918	53800	0.018	0.012	0.007	0.005
(29)	31.0379	1.9346	209288	179649	108041	875030	0.021	0.018	0.011	0.088
Indole (1140)	31.0879	0.0935	7030	6778	5845	10080	0.001	0.001	0.001	0.001
(687)	31.0879	2.9439	28186	26107	20704	41715	0.003	0.003	0.002	0.004
(1028)	31.0879	3.5794	12076	12005	10293	16283	0.001	0.001	0.001	0.002
(1152)	31.1046	3.9626	2148	2285	1689	3026	0.000	0.000	0.000	0.000
(1015)	31.1213	3.3645	11146	10619	8591	14112	0.001	0.001	0.001	0.001
(1275)	31.1379	0.5701	7629	6716	5463	9590	0.001	0.001	0.001	0.001
523 (194)	31.1713	1.1121	258962	194144	89485	120877	0.026	0.019	0.009	0.012
(1309)	31.1713	0.4766	7398	6616	5910	9017	0.001	0.001	0.001	0.001
(1427)	31.2213	0.7290	10892	9878	9842	12245	0.001	0.001	0.001	0.001
(707)	31.2379	2.8224	10617	10647	9124	12881	0.001	0.001	0.001	0.001
(942)	31.3046	1.4393	17457	15179	10640	12577	0.002	0.002	0.001	0.001
(632)	31.3713	1.7196	19803	18474	17843	19460	0.002	0.002	0.002	0.002
(1323)	31.3713	2.6075	11284	11493	8534	9827	0.001	0.001	0.001	0.001
(236)	31.4046	1.1121	197078	158986	72921	82778	0.019	0.016	0.007	0.008
1-Methylnaphthalene (224)	31.4213	2.0748	80642	74487	55518	62956	0.008	0.007	0.006	0.006
(654)	31.4379	2.3551	24710	25491	19731	24060	0.002	0.003	0.002	0.002
524 (189)	31.5546	1.1028	236282	186637	91101	102596	0.023	0.019	0.009	0.010
(573)	31.5546	1.9252	15294	15721	15505	14735	0.002	0.002	0.002	0.001
(290)	31.5713	0.1308	91552	97116	83162	92827	0.009	0.010	0.008	0.009
(1031)	31.5713	2.9439	25914	25736	23775	25376	0.003	0.003	0.002	0.003
(1686)	31.5713	3.5047	7631	7384	7547	6828	0.001	0.001	0.001	0.001
(1384)	31.6213	0.5140	14985	14592	13623	14091	0.001	0.001	0.001	0.001
Theaspirane B* (263)	31.6379	1.4953	120717	112036	70183	74601	0.012	0.011	0.007	0.008
(512)	31.7046	2.0000	37850	38749	27294	33283	0.004	0.004	0.003	0.003
(444)	31.7213	1.6822	34600	38105	18913	21897	0.003	0.004	0.002	0.002
(107)	31.8379	0.8692	215689	181362	153725	158338	0.021	0.018	0.016	0.016
(308)	31.8379	1.1215	71864	56469	27880	38413	0.007	0.006	0.003	0.004
(741)	31.9046	1.6168	14662	15165	10359	11275	0.001	0.002	0.001	0.001
(1719)	31.9546	3.0280	11141	10195	8542	9733	0.001	0.001	0.001	0.001
(1037)	31.9713	2.1308	20024	19221	15860	17547	0.002	0.002	0.002	0.002
(317)	32.0379	1.1215	110622	81848	41024	43247	0.011	0.008	0.004	0.004
(1094)	32.0379	3.6729	18951	17678	15605	17138	0.002	0.002	0.002	0.002
(1425)	32.0379	1.9720	7585	6999	5880	6803	0.001	0.001	0.001	0.001
(1350)	32.0546	2.8411	25049	25352	17643	19090	0.002	0.003	0.002	0.002
(708)	32.1379	1.6168	38990	37433	29737	32371	0.004	0.004	0.003	0.003
(184)	32.2213	1.0654	266674	190131	95965	113403	0.026	0.019	0.010	0.011

			Volume				Percent Response			
Compound Name	RT 1D (min)	RT 2D (s)	HM_1	HM_2	LM_1	LM_2	HM_1	HM_2	LM_1	LM_2
(1553)	32.2213	0.5234	13591	10793	10567	11151	0.001	0.001	0.001	0.001
(1532)	32.2546	0.7477	11066	9689	7732	9435	0.001	0.001	0.001	0.001
(1006)	32.3046	1.7196	14192	12343	8389	10143	0.001	0.001	0.001	0.001
(1184)	32.3379	1.4299	15832	13742	11034	12412	0.002	0.001	0.001	0.001
(1769)	32.3379	2.8692	5859	5042	4413	4529	0.001	0.001	0.000	0.000
(272)	32.3713	1.0654	126673	109839	48081	54475	0.013	0.011	0.005	0.005
(1577)	32.3713	3.5234	7224	5835	5168	5422	0.001	0.001	0.001	0.001
(1651)	32.5046	3.1308	20001	17593	15737	17161	0.002	0.002	0.002	0.002
(1080)	32.5213	1.6916	15392	14152	10100	10668	0.002	0.001	0.001	0.001
(1616)	32.5879	1.9813	7919	7324	5922	6426	0.001	0.001	0.001	0.001
Heptylcyclohexane (61)	32.6379	1.1869	559650	441745	248270	267386	0.055	0.044	0.025	0.027
(455)	32.6379	3.6262	24463	20234	20353	20773	0.002	0.002	0.002	0.002
170 (423)	32.6546	3.5607	29149	37268	28610	31326	0.003	0.004	0.003	0.003
(821)	32.6713	1.5607	39495	37567	27044	29858	0.004	0.004	0.003	0.003
525 (91)	32.7379	1.0935	404781	309844	163933	178158	0.040	0.031	0.017	0.018
(1057)	32.7546	2.1869	9950	10845	6602	8954	0.001	0.001	0.001	0.001
Triacetin (338)	32.8046	2.7850	54561	43326	24053	40385	0.005	0.004	0.002	0.004
(1627)	32.8379	3.1308	11097	11708	8808	9846	0.001	0.001	0.001	0.001
(737)	32.9046	1.7009	17517	16097	13013	14760	0.002	0.002	0.001	0.001
(1683)	32.9213	3.4953	7992	8309	5700	8282	0.001	0.001	0.001	0.001
(223)	33.0046	1.0467	266394	206388	109816	125974	0.026	0.021	0.011	0.013
(1707)	33.0379	2.9813	5996	5828	5018	4949	0.001	0.001	0.001	0.000
(1738)	33.0379	3.7103	7603	7056	5816	7234	0.001	0.001	0.001	0.001
26 (69)	33.1046	0.1028	415715	388270	342612	403624	0.041	0.039	0.035	0.041
(903)	33.1046	1.4673	17421	15149	11585	13095	0.002	0.002	0.001	0.001
(1391)	33.1046	0.7850	6902	6437	6255	6454	0.001	0.001	0.001	0.001
(1408)	33.1213	0.8879	7623	6422	6060	5772	0.001	0.001	0.001	0.001
(1528)	33.1546	2.7196	9950	10592	8823	9482	0.001	0.001	0.001	0.001
(355)	33.1713	2.3178	48687	45575	34232	41638	0.005	0.005	0.003	0.004
(306)	33.2046	1.0748	129559	93803	48748	54685	0.013	0.009	0.005	0.006
(917)	33.2879	1.7103	24485	17574	12836	13962	0.002	0.002	0.001	0.001
Decanoic acid* (239)	33.3713	2.7009	147772	104673	89503	68675	0.015	0.010	0.009	0.007
(509)	33.3713	1.3738	34255	30217	20520	26906	0.003	0.003	0.002	0.003
(1214)	33.3713	3.1589	22278	18502	15751	15500	0.002	0.002	0.002	0.002
(1498)	33.3713	3.4393	8763	7484	5923	5991	0.001	0.001	0.001	0.001
(1535)	33.4046	2.4673	8247	7504	6150	6416	0.001	0.001	0.001	0.001
(320)	33.4713	1.5981	70544	70604	52639	53508	0.007	0.007	0.005	0.005
(595)	33.5046	2.0935	31767	26895	25395	26847	0.003	0.003	0.003	0.003
(1049)	33.5546	1.9533	11819	11193	9559	10723	0.001	0.001	0.001	0.001
(539)	33.5713	3.4299	33063	33088	24300	27520	0.003	0.003	0.002	0.003
(853)	33.5879	1.4673	18765	18311	14670	14275	0.002	0.002	0.001	0.001

Compound Name	RT 1D (min)	RT 2D (s)	Volume HM_1	HM_2	LM_1	LM_2	Percent Response HM_1	HM_2	LM_1	LM_2
(1694)	33.7046	0.5701	8265	7609	5995	6845	0.001	0.001	0.001	0.001
(350)	33.7046	3.7944	54138	51914	47006	49126	0.005	0.005	0.005	0.005
526 (55)	33.7713	1.0748	1011566	807544	437565	498813	0.100	0.081	0.045	0.050
(1624)	33.8213	2.3551	9039	8883	6380	6859	0.001	0.001	0.001	0.001
(1696)	33.8879	2.8318	6869	6641	5090	5094	0.001	0.001	0.001	0.001
(695)	33.9379	1.8037	37802	36928	27179	29278	0.004	0.004	0.003	0.003
528 (137)	33.9713	1.2336	237676	191954	111863	125102	0.023	0.019	0.011	0.013
(153)	34.0046	1.0935	234164	182982	94533	109640	0.023	0.018	0.010	0.011
(1604)	34.0546	3.3551	15726	14242	12765	13710	0.002	0.001	0.001	0.001
Butyl benzoate (108)	34.0713	1.9907	190909	182164	172565	167563	0.019	0.018	0.018	0.017
3-Hydroxy-2,4,4-trimethylpentyl-2-methylpropanoate (187)	34.1046	2.2710	83457	82133	57531	73555	0.008	0.008	0.006	0.007
(1274)	34.1546	2.9533	33450	30058	26090	27466	0.003	0.003	0.003	0.003
(Z)-3-Hexenyl hexanoate* (212)	34.2379	1.5701	485672	512881	52755	73692	0.048	0.051	0.005	0.007
(894)	34.2379	2.4486	16856	14687	10798	12333	0.002	0.001	0.001	0.001
(590)	34.2546	2.2336	36288	24191	23273	19728	0.004	0.002	0.002	0.002
(1756)	34.2713	0.8037	6600	5942	4726	5815	0.001	0.001	0.000	0.001
α-Cubebene (300)	34.3879	1.3832	76537	66922	36211	40328	0.008	0.007	0.004	0.004
(647)	34.4046	2.0374	29106	27843	19261	22132	0.003	0.003	0.002	0.002
(1462)	34.4046	3.2056	17861	15817	13488	14885	0.002	0.002	0.001	0.002
(5E)-3-Methyl-5-undecene (78)	34.4379	1.1308	630888	488159	262138	288165	0.062	0.049	0.027	0.029
(1495)	34.4546	3.4299	12515	12287	10020	11313	0.001	0.001	0.001	0.001
(1777)	34.5379	0.4860	8761	7080	6396	6936	0.001	0.001	0.001	0.001
(1731)	34.5546	0.0654	2151	2039	1655	1649	0.000	0.000	0.000	0.000
(1207)	34.6046	1.8411	11654	10329	6619	6739	0.001	0.001	0.001	0.001
Hexyl hexanoate (313)	34.6213	1.4953	89128	95979	36016	38160	0.009	0.010	0.004	0.004
(1040)	34.6379	2.0561	18005	17486	10284	10019	0.002	0.002	0.001	0.001
(1221)	34.6546	2.5234	25911	23467	20469	20018	0.003	0.002	0.002	0.002
(E)-2-Hexenyl hexanoate (471)	34.6713	1.5794	42689	50532	20827	29287	0.004	0.005	0.002	0.003
5-Tetradecene (66)	34.7046	1.1215	629133	497597	295910	325155	0.062	0.050	0.030	0.033
(923)	34.7713	2.9252	20380	18366	18031	19056	0.002	0.002	0.002	0.002
(1550)	34.7713	2.2056	6257	6523	4735	5238	0.001	0.001	0.000	0.001
β-Bourbonene (335)	34.8046	1.3738	52947	51233	29416	30556	0.005	0.005	0.003	0.003
(855)	34.8379	1.8411	18436	17429	14589	16956	0.002	0.002	0.001	0.002
(1578)	34.8379	2.6636	8838	8224	8138	7559	0.001	0.001	0.001	0.001
(1782)	34.9879	0.3178	6688	5624	5787	5898	0.001	0.001	0.001	0.001
(609)	35.0213	1.7009	25173	22971	18602	20181	0.002	0.002	0.002	0.002
530 (33)	35.0379	1.0748	982607	783105	459725	540229	0.097	0.078	0.047	0.054
(1014)	35.0379	2.0374	17416	15954	13158	12584	0.002	0.002	0.001	0.001
(571)	35.1713	1.6916	20071	16738	13324	13570	0.002	0.002	0.001	0.001
(810)	35.1713	3.7103	90073	64650	35844	36156	0.009	0.006	0.004	0.004
(1650)	35.2046	2.7757	19666	20559	13125	11396	0.002	0.002	0.001	0.001

Compound Name	RT 1D (min)	RT 2D (s)	Volume HM_1	HM_2	LM_1	LM_2	Percent Response HM_1	HM_2	LM_1	LM_2
Jasmone* (939)	35.2379	2.4486	138055	130499	18811	19104	0.014	0.013	0.002	0.002
(1611)	35.2379	0.1589	13418	10849	8300	7728	0.001	0.001	0.001	0.001
2-Methyltridecane (73)	35.3046	1.1215	676080	523558	291362	333231	0.067	0.052	0.030	0.034
(334)	35.3046	1.8224	44994	39973	32588	34712	0.004	0.004	0.003	0.003
(1059)	35.3379	2.4486	35978	29612	19773	25545	0.004	0.003	0.002	0.003
Dodecanal (360)	35.4546	1.8131	113998	49573	51609	39601	0.011	0.005	0.005	0.004
(564)	35.5213	2.0467	39512	34124	30381	28713	0.004	0.003	0.003	0.003
(1203)	35.6879	0.8411	8905	8838	6193	8307	0.001	0.001	0.001	0.001
(222)	35.7046	1.1215	158512	125205	68074	77613	0.016	0.013	0.007	0.008
(1417)	35.7213	1.8318	8821	8467	7376	7618	0.001	0.001	0.001	0.001
(1104)	35.7713	2.3271	14784	13789	12535	12934	0.001	0.001	0.001	0.001
(362)	35.7879	1.4579	74694	67483	42959	44800	0.007	0.007	0.004	0.005
(140)	35.8879	1.1121	235499	178538	95897	115374	0.023	0.018	0.010	0.012
(1531)	35.9046	2.0935	15785	15349	12482	12928	0.002	0.002	0.001	0.001
544 (150)	36.0379	1.1121	301446	231714	127786	150478	0.030	0.023	0.013	0.015
(685)	36.0546	1.4299	25031	22878	16522	18300	0.002	0.002	0.002	0.002
(920)	36.1213	1.6449	24691	22226	18259	19569	0.002	0.002	0.002	0.002
(433)	36.1713	2.0748	54026	52426	51685	48401	0.005	0.005	0.005	0.005
(1710)	36.2046	2.3832	6494	6474	5333	6053	0.001	0.001	0.001	0.001
(1146)	36.2379	3.4486	18760	18961	16606	18296	0.002	0.002	0.002	0.002
(321)	36.3213	1.1308	178153	140780	84117	90378	0.018	0.014	0.009	0.009
β-Caryophyllene* (131)	36.3213	1.4860	334308	292619	101336	115171	0.033	0.029	0.010	0.012
(1112)	36.4379	2.8411	15071	14005	11535	12900	0.001	0.001	0.001	0.001
α-Ionone* (172)	36.4713	2.1869	110809	107606	84093	86856	0.011	0.011	0.009	0.009
(1118)	36.5213	1.7477	13997	13239	10356	11475	0.001	0.001	0.001	0.001
(203)	36.6379	1.0841	359700	283260	169281	196539	0.036	0.028	0.017	0.020
(1533)	36.6713	2.3364	7469	6727	5568	5280	0.001	0.001	0.001	0.001
(1500)	36.6879	3.4206	20838	19747	18710	18548	0.002	0.002	0.002	0.002
(652)	36.7046	1.4206	26001	24328	16773	16799	0.003	0.002	0.002	0.002
(1639)	36.7046	1.9439	7015	6917	6535	6187	0.001	0.001	0.001	0.001
(1209)	36.7546	1.6822	9499	7789	7250	8006	0.001	0.001	0.001	0.001
(1090)	36.9546	2.0093	12121	12417	9385	8753	0.001	0.001	0.001	0.001
(979)	37.0213	2.1121	18147	18078	15307	15974	0.002	0.002	0.002	0.002
Nonylcyclopentane (31)	37.1713	1.1963	1083517	880681	592245	693866	0.107	0.088	0.060	0.070
(762)	37.1713	1.6822	26438	24414	19598	24133	0.003	0.002	0.002	0.002
(1095)	37.1713	2.1121	13774	12149	10983	12955	0.001	0.001	0.001	0.001
(1297)	37.3379	2.8598	15439	14697	15131	15699	0.002	0.001	0.002	0.002
(1491)	37.3546	3.4766	18863	19746	17655	18932	0.002	0.002	0.002	0.002
(Z)-Geranyl acetone* (45)	37.3713	1.9346	301026	297814	334314	369735	0.030	0.030	0.034	0.037
(461)	37.3879	1.6822	32227	30929	30180	29867	0.003	0.003	0.003	0.003
536 (202)	37.4713	1.1308	247242	191862	128086	141327	0.024	0.019	0.013	0.014

			Volume				Percent Response			
Compound Name	RT 1D (min)	RT 2D (s)	HM_1	HM_2	LM_1	LM_2	HM_1	HM_2	LM_1	LM_2
Vanillin (1100)	37.5046	2.3925	8323	7988	7337	8233	0.001	0.001	0.001	0.001
(1366)	37.5713	2.8785	15996	14626	14194	13475	0.002	0.001	0.001	0.001
(1757)	37.6046	0.5140	8647	7791	7081	7746	0.001	0.001	0.001	0.001
(496)	37.6213	1.3925	37098	35755	25524	27531	0.004	0.004	0.003	0.003
537 (60)	37.6879	1.0841	603146	471453	343195	423263	0.060	0.047	0.035	0.043
(1007)	37.7046	2.1402	21678	20659	18340	20167	0.002	0.002	0.002	0.002
(1662)	37.7379	3.7664	11192	10889	10274	11261	0.001	0.001	0.001	0.001
(802)	37.7713	1.5234	26433	23593	14777	15525	0.003	0.002	0.002	0.002
(1779)	37.7713	3.3084	6831	6225	5103	5433	0.001	0.001	0.001	0.001
(954)	37.9713	1.7570	9063	8455	7191	8154	0.001	0.001	0.001	0.001
(196)	38.0046	1.1215	280731	217556	132162	158574	0.028	0.022	0.013	0.016
(411)	38.0046	1.4860	75157	70488	52408	54781	0.007	0.007	0.005	0.006
(986)	38.0879	3.3084	23552	20984	18592	23846	0.002	0.002	0.002	0.002
(1675)	38.1213	3.0654	10753	11240	9639	10459	0.001	0.001	0.001	0.001
(1689)	38.1546	2.9065	7140	6101	5840	7028	0.001	0.001	0.001	0.001
Dodecanol (167)	38.1713	1.8598	65440	57579	43172	84372	0.006	0.006	0.004	0.009
(469)	38.1713	2.1402	21875	20030	17602	22090	0.002	0.002	0.002	0.002
(543)	38.1879	2.4206	41977	39821	32123	42957	0.004	0.004	0.003	0.004
(1714)	38.2713	0.4579	7834	6549	6412	6710	0.001	0.001	0.001	0.001
(1725)	38.2713	0.3084	5605	4900	4832	5455	0.001	0.000	0.000	0.001
(818)	38.3713	1.4393	28628	25604	18762	21391	0.003	0.003	0.002	0.002
(214)	38.4046	1.1215	272709	222037	134900	163918	0.027	0.022	0.014	0.017
(688)	38.4213	1.8785	20069	20212	17144	27719	0.002	0.002	0.002	0.003
(1715)	38.4379	0.2710	6619	5564	5147	7038	0.001	0.001	0.001	0.001
(1601)	38.5379	3.4766	14227	12734	11637	14225	0.001	0.001	0.001	0.001
(1256)	38.6213	1.7477	8531	7653	6675	8060	0.001	0.001	0.001	0.001
(1737)	38.6213	3.2243	4333	4815	4235	4532	0.000	0.000	0.000	0.000
(636)	38.6379	1.4206	27757	24579	15381	17202	0.003	0.002	0.002	0.002
(179)	38.6879	1.1589	186065	146288	98529	109548	0.018	0.015	0.010	0.011
(399)	38.6879	2.0561	33130	30340	27416	29677	0.003	0.003	0.003	0.003
(936)	38.7046	2.9813	18740	17937	19158	20876	0.002	0.002	0.002	0.002
(1177)	38.7713	2.2804	12164	12420	10938	11503	0.001	0.001	0.001	0.001
538 (74)	38.9046	1.1589	550252	435618	277533	342975	0.054	0.044	0.028	0.035
(499)	38.9046	1.4673	33285	31332	20622	27681	0.003	0.003	0.002	0.003
(921)	38.9046	1.7757	16136	14475	12571	14936	0.002	0.001	0.001	0.002
β-Ionone* (64)	38.9213	2.2056	332592	314968	264273	276747	0.033	0.032	0.027	0.028
β-Ionone epoxide (125)	38.9213	2.5047	149103	134837	141514	151382	0.015	0.013	0.014	0.015
(1592)	38.9213	3.1589	8668	8891	6981	7215	0.001	0.001	0.001	0.001
Jasmine lactone* (1720)	39.0213	3.6168	11558	10073	9795	9360	0.001	0.001	0.001	0.001
(1023)	39.0879	3.1402	12461	11225	9834	12904	0.001	0.001	0.001	0.001
(438)	39.1046	1.8131	47586	46082	36942	44914	0.005	0.005	0.004	0.005

Compound Name	RT 1D (min)	RT 2D (s)	Volume				Percent Response			
			HM_1	HM_2	LM_1	LM_2	HM_1	HM_2	LM_1	LM_2
(1670)	39.2046	3.5794	12582	10845	8093	7916	0.001	0.001	0.001	0.001
Pentadecane (99)	39.2213	1.0935	556208	470533	274347	347647	0.055	0.047	0.028	0.035
(136)	39.2379	0.9065	66198	60939	51008	95361	0.007	0.006	0.005	0.010
(1231)	39.2713	2.0467	14733	14268	12743	14571	0.001	0.001	0.001	0.001
(867)	39.3046	2.9346	20338	21326	21938	24385	0.002	0.002	0.002	0.002
(234)	39.5046	1.1402	97700	78395	51089	59867	0.010	0.008	0.005	0.006
(1552)	39.5046	2.2523	8190	6829	5760	6387	0.001	0.001	0.001	0.001
(E,E)-α-Farnesene* (547)	39.6213	1.3832	71927	68035	33787	35827	0.007	0.007	0.003	0.004
(208)	39.6546	1.1308	214016	176061	99983	125264	0.021	0.018	0.010	0.013
(505)	39.7046	1.7944	55055	31640	28329	32493	0.005	0.003	0.003	0.003
(1311)	39.7213	2.0280	20572	19112	16437	15895	0.002	0.002	0.002	0.002
2,4-Di-tert-butylphenol (918)	39.8213	2.4206	22760	19610	14326	15221	0.002	0.002	0.001	0.002
(279)	39.8546	1.1308	174300	134610	80657	99122	0.017	0.013	0.008	0.010
(1116)	39.9379	2.3738	11270	10370	10788	11550	0.001	0.001	0.001	0.001
(1242)	39.9713	2.7850	12921	11331	11539	12108	0.001	0.001	0.001	0.001
(1266)	39.9713	2.0187	7277	7144	5857	6492	0.001	0.001	0.001	0.001
Butylated hydroxytoluene (168)	39.9879	1.7196	92916	82654	78058	87873	0.009	0.008	0.008	0.009
(1229)	40.0213	2.2243	25982	22917	22694	24781	0.003	0.002	0.002	0.002
(284)	40.1379	1.1589	133838	105016	68934	78935	0.013	0.011	0.007	0.008
(1594)	40.1546	2.8598	10440	9509	8905	9918	0.001	0.001	0.001	0.001
(529)	40.2213	1.4579	55372	49778	31779	38423	0.005	0.005	0.003	0.004
(337)	40.2379	3.2897	46354	39072	44719	47240	0.005	0.004	0.005	0.005
(1373)	40.2879	1.8598	12508	11544	10130	11159	0.001	0.001	0.001	0.001
(1371)	40.3046	2.0467	10533	10047	9471	10627	0.001	0.001	0.001	0.001
(250)	40.4713	1.1215	213029	171768	110970	133668	0.021	0.017	0.011	0.013
Butyl methyl phthalate (65)	40.5046	2.8131	412111	384681	378462	369977	0.041	0.039	0.039	0.037
δ-Cadinene (515)	40.5046	1.4112	53111	51986	23528	26294	0.005	0.005	0.002	0.003
(1457)	40.5213	3.6075	6182	6199	4940	5444	0.001	0.001	0.001	0.001
(1529)	40.5213	3.7383	11563	11898	10394	11233	0.001	0.001	0.001	0.001
(1692)	40.5213	2.4860	9816	8903	8259	9410	0.001	0.001	0.001	0.001
(Z)-Calamenene (623)	40.5546	1.5981	30778	33155	21351	23619	0.003	0.003	0.002	0.002
(1239)	40.5713	2.0561	12828	11439	11354	12068	0.001	0.001	0.001	0.001
(1724)	40.5713	0.4393	6916	6822	5573	6605	0.001	0.001	0.001	0.001
(626)	40.6213	1.7757	14921	13780	11103	16430	0.001	0.001	0.001	0.002
(1344)	40.6213	2.2523	9268	9518	8499	10279	0.001	0.001	0.001	0.001
(215)	40.7046	1.1402	168332	134750	84219	107755	0.017	0.013	0.009	0.011
(1107)	40.7879	2.0654	13272	12884	13549	13081	0.001	0.001	0.001	0.001
(1584)	40.8046	2.7009	9462	9335	8528	9543	0.001	0.001	0.001	0.001
(245)	40.9546	1.1963	167509	137167	97371	123403	0.017	0.014	0.010	0.012
(1764)	40.9546	3.1308	4697	4694	4763	4983	0.000	0.000	0.000	0.001
Dihydroactinidiolide (135)	40.9713	0.0374	101491	99508	79551	108898	0.010	0.010	0.008	0.011

Compound Name	RT 1D (min)	RT 2D (s)	Volume				Percent Response			
			HM_1	HM_2	LM_1	LM_2	HM_1	HM_2	LM_1	LM_2
(1064)	40.9713	1.6729	16602	16879	13472	14705	0.002	0.002	0.001	0.001
(1398)	40.9713	0.6449	11018	11197	10904	12106	0.001	0.001	0.001	0.001
(1499)	40.9713	0.8224	6715	6756	6258	6557	0.001	0.001	0.001	0.001
(201)	41.0879	1.1215	127213	97083	63930	76777	0.013	0.010	0.007	0.008
(1518)	41.0879	2.4299	14631	14246	15508	13242	0.001	0.001	0.002	0.001
(1745)	41.2546	0.8037	6309	5479	5026	5743	0.001	0.001	0.001	0.001
540 (112)	41.2713	1.1402	356454	270705	186404	218073	0.035	0.027	0.019	0.022
(1219)	41.3046	2.0841	8466	8218	8364	7719	0.001	0.001	0.001	0.001
(1761)	41.3213	0.5140	9296	7873	7824	7598	0.001	0.001	0.001	0.001
(844)	41.3713	1.6729	29510	27585	19401	19117	0.003	0.003	0.002	0.002
(1005)	41.3713	2.8598	21508	19327	18050	20737	0.002	0.002	0.002	0.002
Nonylcyclohexane (67)	41.4213	1.2336	433009	365255	218271	288097	0.043	0.037	0.022	0.029
(1679)	41.4379	2.3458	6232	5649	8364	5506	0.001	0.001	0.001	0.001
(1733)	41.4379	2.5888	8274	6828	6989	7136	0.001	0.001	0.001	0.001
(1069)	41.5546	1.9159	36850	28275	27806	24803	0.004	0.003	0.003	0.002
(1211)	41.6046	2.1495	12541	9831	13447	10383	0.001	0.001	0.001	0.001
(1412)	41.6879	1.6449	9618	8442	6991	7005	0.001	0.001	0.001	0.001
(1490)	41.7046	3.4393	17242	16172	18338	16121	0.002	0.002	0.002	0.002
(294)	41.8379	1.1402	182640	145460	90927	109134	0.018	0.015	0.009	0.011
(1133)	41.8546	2.0561	22789	19727	14137	12141	0.002	0.002	0.001	0.001
(E)-Nerolidol* (907)	41.9046	1.7570	37105	36741	21889	22005	0.004	0.004	0.002	0.002
(619)	41.9379	1.4299	24771	25552	15492	18537	0.002	0.003	0.002	0.002
Dodecanoic acid (796)	41.9546	2.4206	39765	26165	25965	19159	0.004	0.003	0.003	0.002
(1626)	41.9713	2.6542	8934	8577	7592	8082	0.001	0.001	0.001	0.001
(569)	42.0546	2.1028	29694	26581	28213	23158	0.003	0.003	0.003	0.002
(122)	42.0713	1.1215	273090	215813	137005	164341	0.027	0.022	0.014	0.017
(1125)	42.1379	2.9065	22357	18278	41425	16246	0.002	0.002	0.004	0.002
(1067)	42.2213	1.5140	13618	14845	8596	9878	0.001	0.001	0.001	0.001
(376)	42.2379	1.1682	61001	46852	29302	36891	0.006	0.005	0.003	0.004
2,3,6-Trimethylnaphthalene (386)	42.2546	2.0748	64012	55287	50872	48454	0.006	0.006	0.005	0.005
(570)	42.2546	1.8131	26012	22957	15646	25562	0.003	0.002	0.002	0.003
(943)	42.4713	2.0187	8527	8062	8905	8571	0.001	0.001	0.001	0.001
(1079)	42.4879	0.8598	15338	14508	11581	14468	0.002	0.001	0.001	0.001
(278)	42.5046	1.1776	115569	87320	55999	70163	0.011	0.009	0.006	0.007
(385)	42.5046	1.3832	64759	58682	37828	43555	0.006	0.006	0.004	0.004
(1225)	42.5546	1.9533	12802	11063	12549	9494	0.001	0.001	0.001	0.001
(155)	42.7046	1.2710	211780	171436	110840	135376	0.021	0.017	0.011	0.014
452 (244)	42.8213	2.1028	163155	114510	131234	91884	0.016	0.011	0.013	0.009
(1580)	42.8379	2.7009	10294	8089	17659	6700	0.001	0.001	0.002	0.001
(1536)	42.8879	3.7850	12660	10929	9976	10270	0.001	0.001	0.001	0.001
(249)	42.9546	1.1589	186971	143842	115172	108589	0.018	0.014	0.012	0.011

Compound Name	RT 1D (min)	RT 2D (s)	Volume				Percent Response			
			HM_1	HM_2	LM_1	LM_2	HM_1	HM_2	LM_1	LM_2
(1471)	43.0046	1.7477	17326	14676	15229	12420	0.002	0.001	0.002	0.001
Caryophyllene oxide (526)	43.0879	2.1121	52876	44914	22958	24711	0.005	0.004	0.002	0.002
(1142)	43.1213	0.4860	24837	15837	25652	14949	0.002	0.002	0.003	0.002
Hexadecane (113)	43.2879	1.1121	338869	289848	191749	205812	0.033	0.029	0.020	0.021
2,2,4-Trimethyl-1,3-pentanediol diisobutyrate (104)	43.4213	1.8505	320215	276871	214060	254070	0.032	0.028	0.022	0.026
(881)	43.4213	2.4206	20444	9410	6779	6083	0.002	0.001	0.001	0.001
(101)	43.4713	1.1589	561357	461799	354578	393252	0.055	0.046	0.036	0.040
(327)	43.4879	1.4486	48424	41449	33015	33064	0.005	0.004	0.003	0.003
(216)	43.6046	0.8131	170205	155785	148086	137556	0.017	0.016	0.015	0.014
(2)	43.6879	3.3178	478641787	491641187	529876556	472084561	47.255	49.223	53.955	47.583
Ethylpentyl phtalate (4)	43.8046	3.1308	218954294	214169234	218397475	249036793	21.617	21.443	22.239	25.101
(1)	43.8213	3.2804	77602531	77596054	68130443	77640404	7.661	7.769	6.937	7.826
(2,4,4-Trimethyl-3-(2-methylpropanoyloxy)pentyl) 2-methylpropanoate (181)	43.8546	1.5794	123281	82718	73437	64015	0.012	0.008	0.007	0.006
(133)	43.8546	1.4486	544414	391581	305957	346252	0.054	0.039	0.031	0.035
(3)	43.8713	0.0374	3805671	3407198	3231418	3340558	0.376	0.341	0.329	0.337
(248)	43.8713	2.1869	189669	122520	108604	91154	0.019	0.012	0.011	0.009
(115)	43.8879	1.1215	855381	626384	511998	570981	0.084	0.063	0.052	0.058
(251)	43.8879	2.2897	104423	54343	60839	50142	0.010	0.005	0.006	0.005
(130)	43.9046	1.7383	524677	349571	290441	315402	0.052	0.035	0.030	0.032
(211)	43.9046	2.0280	196389	115949	110061	117906	0.019	0.012	0.011	0.012
(735)	44.1546	3.7757	39436	35110	34197	34647	0.004	0.004	0.003	0.003
(635)	44.1879	1.7850	26878	21105	17834	22136	0.003	0.002	0.002	0.002
(658)	44.1879	1.9626	36091	30871	23115	26043	0.004	0.003	0.002	0.003
(689)	44.1879	0.7383	45505	37479	33533	37811	0.004	0.004	0.003	0.004
(727)	44.1879	2.1215	29031	25307	22616	24229	0.003	0.003	0.002	0.002
(621)	44.2046	0.0467	19797	14342	14872	17336	0.002	0.001	0.002	0.002
(612)	44.2046	3.2617	77648	67138	65271	71434	0.008	0.007	0.007	0.007
(668)	44.2379	0.5234	24910	20893	18870	23124	0.002	0.002	0.002	0.002
(614)	44.2713	2.8972	22811	22361	20398	22354	0.002	0.002	0.002	0.002
(771)	44.2879	3.6168	31263	29073	28001	30564	0.003	0.003	0.003	0.003
(397)	44.3046	1.4673	80477	67043	54559	70569	0.008	0.007	0.006	0.007
(880)	44.3546	0.3084	37929	36486	33053	36056	0.004	0.004	0.003	0.004
(325)	44.3713	1.1589	126302	104642	75683	89536	0.012	0.010	0.008	0.009
(959)	44.4379	0.8411	18094	16618	15226	15885	0.002	0.002	0.002	0.002
(983)	44.5213	0.2056	12686	13172	12490	12692	0.001	0.001	0.001	0.001
(1038)	44.5213	3.8318	11516	12458	10245	11010	0.001	0.001	0.001	0.001
(126)	44.5546	2.5701	208932	209886	207699	213023	0.021	0.021	0.021	0.021
(661)	44.5879	1.6822	20894	20780	18725	20296	0.002	0.002	0.002	0.002
(371)	44.6046	1.1308	83199	66835	50456	56330	0.008	0.007	0.005	0.006
(967)	44.6046	0.5047	20985	19657	18323	18219	0.002	0.002	0.002	0.002
(377)	44.6213	1.3738	37693	36411	31769	31619	0.004	0.004	0.003	0.003

Compound Name	RT 1D (min)	RT 2D (s)	Volume				Percent Response			
			HM_1	HM_2	LM_1	LM_2	HM_1	HM_2	LM_1	LM_2
(195)	44.6546	2.2804	67963	71545	77161	76374	0.007	0.007	0.008	0.008
(1076)	44.7379	0.1963	12710	11853	10971	11884	0.001	0.001	0.001	0.001
(225)	44.7546	2.2336	221585	225272	223219	231820	0.022	0.023	0.023	0.023
(261)	44.7546	1.3832	51215	53076	43926	52594	0.005	0.005	0.004	0.005
(684)	44.8213	2.6168	20783	21944	18286	21563	0.002	0.002	0.002	0.002
(1010)	44.8213	1.8972	22014	20834	15268	15661	0.002	0.002	0.002	0.002
(1162)	44.8213	3.8411	9864	9935	9291	10712	0.001	0.001	0.001	0.001
(439)	44.8546	1.1589	65299	51587	38678	42307	0.006	0.005	0.004	0.004
(1110)	44.8546	0.0654	8626	7280	7108	8423	0.001	0.001	0.001	0.001
(1050)	44.8879	1.6262	16057	13314	11618	12187	0.002	0.001	0.001	0.001
(445)	44.9546	1.1215	50474	41562	24991	30974	0.005	0.004	0.003	0.003
(1264)	44.9546	0.7944	10087	9498	10455	10570	0.001	0.001	0.001	0.001
(1298)	44.9713	3.7009	13933	15780	13431	13591	0.001	0.002	0.001	0.001
(285)	45.0213	3.1028	63963	77299	80647	68802	0.006	0.008	0.008	0.007
(1191)	45.0213	3.5421	11822	10971	11718	12652	0.001	0.001	0.001	0.001
(1220)	45.0546	0.1682	10214	10543	10363	10065	0.001	0.001	0.001	0.001
(378)	45.0879	1.1215	112120	89529	41777	50384	0.011	0.009	0.004	0.005
(352)	45.1046	1.3925	62994	59023	46207	51736	0.006	0.006	0.005	0.005
(1376)	45.1213	0.8131	8021	7218	7506	8031	0.001	0.001	0.001	0.001
(1332)	45.1879	0.2336	12690	12201	11576	12754	0.001	0.001	0.001	0.001
(274)	45.2713	2.1121	81707	87186	74220	80671	0.008	0.009	0.008	0.008
(256)	45.2879	1.1869	82900	68019	47066	51366	0.008	0.007	0.005	0.005
(686)	45.3046	1.9720	46364	49909	26602	19916	0.005	0.005	0.003	0.002
(1424)	45.3046	0.8879	8097	6742	7455	7646	0.001	0.001	0.001	0.001
(1379)	45.3546	3.9533	3431	2667	2746	3309	0.000	0.000	0.000	0.000
(310)	45.3879	2.0935	58653	64963	52863	56489	0.006	0.007	0.005	0.006
543 (109)	45.4213	1.2243	245383	205053	133092	150936	0.024	0.021	0.014	0.015
(1172)	45.4713	3.6168	9507	9124	8208	9639	0.001	0.001	0.001	0.001
(1244)	45.4713	3.7477	12720	11717	11879	12456	0.001	0.001	0.001	0.001
(Z)-Methyl dihydrojasmonate (47)	45.4879	2.5327	214627	173984	157286	428930	0.021	0.017	0.016	0.043
(838)	45.5046	3.3832	17235	16932	15086	18141	0.002	0.002	0.002	0.002
(1339)	45.5213	0.2710	16546	16367	16350	16973	0.002	0.002	0.002	0.002
(1290)	45.5379	0.1121	6169	6699	6154	6989	0.001	0.001	0.001	0.001
(1524)	45.5546	0.8598	11353	10371	10651	10395	0.001	0.001	0.001	0.001
(319)	45.5879	2.0467	67634	69979	64489	65220	0.007	0.007	0.007	0.007
(1143)	45.6546	1.7664	12835	12698	10300	13007	0.001	0.001	0.001	0.001
(252)	45.6546	1.1589	172918	148935	84681	127124	0.017	0.015	0.009	0.013
(1070)	45.7046	2.2897	11232	11605	8918	11316	0.001	0.001	0.001	0.001
(1190)	45.7379	2.6822	9206	8005	7065	8230	0.001	0.001	0.001	0.001
(1522)	45.7379	3.6168	9237	8501	7476	8662	0.001	0.001	0.001	0.001
3,4-Diethyl-1,1'-biphenyl (311)	45.7546	2.0187	114351	118459	86984	96034	0.011	0.012	0.009	0.010

Compound Name	RT 1D (min)	RT 2D (s)	Volume HM_1	HM_2	LM_1	LM_2	Percent Response HM_1	HM_2	LM_1	LM_2
(1390)	45.7713	3.0093	12168	10995	9336	11457	0.001	0.001	0.001	0.001
(1418)	45.7879	0.1402	8411	8554	8329	8294	0.001	0.001	0.001	0.001
(1560)	45.7879	3.8131	10603	9612	8505	8883	0.001	0.001	0.001	0.001
(292)	45.8879	0.9346	46729	41592	34731	46903	0.005	0.004	0.004	0.005
(359)	45.8879	1.4206	52416	46982	36144	43820	0.005	0.005	0.004	0.004
(503)	45.8879	1.1589	60409	51368	30580	37865	0.006	0.005	0.003	0.004
(1582)	46.0879	3.6636	14680	13962	13400	13196	0.001	0.001	0.001	0.001
(1158)	46.1379	2.5327	11177	10822	9510	10689	0.001	0.001	0.001	0.001
(164)	46.1546	2.0187	113157	108643	108439	159629	0.011	0.011	0.011	0.016
(322)	46.1546	1.8318	61325	53412	47788	52660	0.006	0.005	0.005	0.005
(551)	46.1713	1.1869	75604	63088	41843	47309	0.007	0.006	0.004	0.005
(650)	46.1713	1.5794	27370	26597	21827	18089	0.003	0.003	0.002	0.002
(1331)	46.1713	3.0467	14973	15830	14444	15014	0.001	0.002	0.001	0.002
(1661)	46.1713	3.9065	3952	4275	4018	3864	0.000	0.000	0.000	0.000
(1505)	46.1879	3.5327	10345	9684	8369	9514	0.001	0.001	0.001	0.001
(480)	46.3713	2.0748	31537	31063	28501	36961	0.003	0.003	0.003	0.004
(873)	46.3713	2.6449	15564	15339	13909	15234	0.002	0.002	0.001	0.002
(1497)	46.4046	0.4860	13556	12875	11771	11944	0.001	0.001	0.001	0.001
(403)	46.4213	1.8692	33259	34343	32751	35039	0.003	0.003	0.003	0.004
(908)	46.4213	2.9065	17088	18279	15537	19881	0.002	0.002	0.002	0.002
(1058)	46.4213	3.1215	20808	19685	17592	20986	0.002	0.002	0.002	0.002
(1658)	46.4213	3.8037	8107	9044	8656	8930	0.001	0.001	0.001	0.001
(329)	46.5046	2.5607	44830	44039	36318	64409	0.004	0.004	0.004	0.006
(464)	46.5046	1.1963	118922	101190	73061	80229	0.012	0.010	0.007	0.008
(1527)	46.5213	0.8224	6463	5518	5351	5765	0.001	0.001	0.001	0.001
(1623)	46.5213	3.5421	8680	8076	7987	8031	0.001	0.001	0.001	0.001
(1648)	46.5546	3.9346	7037	6756	6214	7229	0.001	0.001	0.001	0.001
Bancroftinone (435)	46.5713	1.8505	67204	64978	49833	51756	0.007	0.007	0.005	0.005
(346)	46.7046	1.1963	104797	86355	60939	68616	0.010	0.009	0.006	0.007
(1277)	46.7713	2.9626	23613	23135	20676	26273	0.002	0.002	0.002	0.003
(1647)	46.7879	3.4860	11409	12877	11525	12467	0.001	0.001	0.001	0.001
(408)	46.8213	1.7850	40329	41386	31203	35896	0.004	0.004	0.003	0.004
(1327)	46.8379	2.3458	15463	16250	14549	16546	0.002	0.002	0.001	0.002
(336)	46.8379	1.5888	42223	38933	28600	47338	0.004	0.004	0.003	0.005
(1237)	46.8713	2.1682	9541	10163	9545	9127	0.001	0.001	0.001	0.001
(1672)	46.9213	0.1495	9272	8499	8198	8551	0.001	0.001	0.001	0.001
(344)	46.9546	1.1308	84004	75032	46668	55539	0.008	0.008	0.005	0.006
(1645)	46.9713	3.7290	6132	6577	5613	5537	0.001	0.001	0.001	0.001
(450)	46.9879	1.7477	55649	55261	49665	54979	0.005	0.006	0.005	0.006
(1230)	47.0213	2.1215	8908	8745	8511	9658	0.001	0.001	0.001	0.001
(1682)	47.0379	3.5234	5646	5565	5816	6205	0.001	0.001	0.001	0.001

Compound Name	RT 1D (min)	RT 2D (s)	Volume HM_1	HM_2	LM_1	LM_2	Percent Response HM_1	HM_2	LM_1	LM_2
(1680)	47.1713	0.6729	14464	12754	14479	13717	0.001	0.001	0.001	0.001
(424)	47.1713	1.1215	116663	95952	43174	56841	0.012	0.010	0.004	0.006
(517)	47.1879	1.7196	51309	50250	43471	48763	0.005	0.005	0.004	0.005
(483)	47.2379	1.4579	33740	29152	21914	26319	0.003	0.003	0.002	0.003
(696)	47.2379	2.1121	25600	23419	23025	28734	0.003	0.002	0.002	0.003
(1542)	47.2379	3.0841	7476	6549	6968	7932	0.001	0.001	0.001	0.001
(1285)	47.2713	2.3458	10702	10618	12962	11201	0.001	0.001	0.001	0.001
(1717)	47.2879	3.6542	6461	5295	6251	6259	0.001	0.001	0.001	0.001
(1656)	47.3046	3.2617	8980	8032	8044	8300	0.001	0.001	0.001	0.001
(281)	47.3879	1.1402	74006	64676	37674	70098	0.007	0.006	0.004	0.007
(1768)	47.4879	0.9533	8226	7362	7208	7077	0.001	0.001	0.001	0.001
(1316)	47.6046	2.2991	8128	8300	11297	8639	0.001	0.001	0.001	0.001
(562)	47.6379	1.6355	39170	35561	27255	33437	0.004	0.004	0.003	0.003
(449)	47.6379	1.8879	59172	53221	41137	52312	0.006	0.005	0.004	0.005
(1430)	47.6379	2.4953	6006	6534	5477	6130	0.001	0.001	0.001	0.001
(1461)	47.6713	3.8224	10014	9272	8551	9482	0.001	0.001	0.001	0.001
(1484)	47.6713	2.7103	17643	16121	15832	17538	0.002	0.002	0.002	0.002
(589)	47.7713	1.2617	106143	91410	54175	70058	0.010	0.009	0.006	0.007
(591)	47.7879	1.6075	28000	23701	16701	21928	0.003	0.002	0.002	0.002
(524)	47.8213	1.7570	35746	26674	26105	26523	0.004	0.003	0.003	0.003
(1218)	47.8546	2.2523	15756	17464	18980	16719	0.002	0.002	0.002	0.002
(1621)	47.8713	3.4299	15817	15119	13865	15549	0.002	0.002	0.001	0.002
(1735)	47.9046	3.6075	10808	10516	9282	10175	0.001	0.001	0.001	0.001
(559)	47.9713	2.5327	34909	32800	29250	32280	0.003	0.003	0.003	0.003
(624)	48.0046	1.5607	19054	19398	11857	16509	0.002	0.002	0.001	0.002
(1539)	48.0046	2.8318	16657	15411	15190	15082	0.002	0.002	0.002	0.002
(1565)	48.0379	3.1963	7232	7870	6510	7855	0.001	0.001	0.001	0.001
(1213)	48.0546	1.8505	15826	15009	11206	13398	0.002	0.002	0.001	0.001
(598)	48.0879	1.1869	32008	27473	18970	24337	0.003	0.003	0.002	0.002
(379)	48.1713	1.3832	51786	47985	34751	42711	0.005	0.005	0.004	0.004
(575)	48.2213	1.1776	44284	38594	25288	33922	0.004	0.004	0.003	0.003
(1296)	48.2546	2.1869	15279	16318	21112	15824	0.002	0.002	0.002	0.002
(1775)	48.2713	3.3551	5275	5523	4700	4957	0.001	0.001	0.000	0.000
(1236)	48.2879	1.8224	11636	11692	7984	10406	0.001	0.001	0.001	0.001
(1716)	48.3379	3.9346	5860	5896	5258	6052	0.001	0.001	0.001	0.001
(407)	48.3546	1.3925	61759	58906	35101	53283	0.006	0.006	0.004	0.005
(909)	48.4213	2.5421	20382	20351	18161	17046	0.002	0.002	0.002	0.002
(1781)	48.4213	0.4019	5659	5481	4640	4910	0.001	0.001	0.000	0.000
(706)	48.5046	1.1776	18086	17779	11050	14254	0.002	0.002	0.001	0.001
(949)	48.5213	2.1308	24987	25926	27501	25826	0.002	0.003	0.003	0.003
(577)	48.5879	1.1589	43824	37699	24265	31912	0.004	0.004	0.002	0.003

			Volume				Percent Response			
**Compound Name**	**RT 1D (min)**	**RT 2D (s)**	**HM_1**	**HM_2**	**LM_1**	**LM_2**	**HM_1**	**HM_2**	**LM_1**	**LM_2**
(1677)	48.6713	2.5327	7601	7439	7413	7603	0.001	0.001	0.001	0.001
(1020)	48.7046	2.0935	23648	22922	24501	23265	0.002	0.002	0.002	0.002
(1551)	48.7046	3.3925	8834	7625	7635	8083	0.001	0.001	0.001	0.001
(501)	48.7713	1.4206	59533	55458	36109	43332	0.006	0.006	0.004	0.004
(1688)	48.8546	2.7570	9006	8995	9003	8413	0.001	0.001	0.001	0.001
(1673)	48.8879	0.8318	13378	12813	11384	13404	0.001	0.001	0.001	0.001
(286)	48.9213	1.1589	90437	75226	50346	97067	0.009	0.008	0.005	0.010
(709)	49.0046	2.2991	30767	26188	27687	28737	0.003	0.003	0.003	0.003
(813)	49.0046	1.4206	22895	21818	12417	18295	0.002	0.002	0.001	0.002
(1353)	49.0213	1.8505	13685	12117	9719	11329	0.001	0.001	0.001	0.001
(1362)	49.0546	2.4299	12234	10458	10459	10067	0.001	0.001	0.001	0.001
(941)	49.1046	2.0467	9307	9623	12500	10822	0.001	0.001	0.001	0.001
(1762)	49.1046	2.8318	6208	5490	6525	5949	0.001	0.001	0.001	0.001
Undecylcyclohexane (339)	49.2379	1.2617	112641	100229	59155	83219	0.011	0.010	0.006	0.008
(897)	49.2379	2.0000	16988	16312	20639	18759	0.002	0.002	0.002	0.002
(1638)	49.3379	2.7850	8922	8875	7762	7572	0.001	0.001	0.001	0.001
(961)	49.4046	1.9720	10386	9449	12373	10227	0.001	0.001	0.001	0.001
(533)	49.5213	1.1589	54856	47473	28978	45918	0.005	0.005	0.003	0.005
(586)	49.5546	1.4299	35206	29836	20364	23314	0.003	0.003	0.002	0.002
(1746)	49.6046	3.0280	5374	5404	5595	5329	0.001	0.001	0.001	0.001
(1295)	49.6213	2.7196	8629	8939	8061	8579	0.001	0.001	0.001	0.001
(947)	49.6379	1.9720	25532	27830	25336	27435	0.003	0.003	0.003	0.003
(1449)	49.6379	2.4673	9248	9039	7665	8272	0.001	0.001	0.001	0.001
(1701)	49.7379	2.9252	6786	5964	5782	5916	0.001	0.001	0.001	0.001
(1378)	49.8213	1.5607	9265	7549	5500	6493	0.001	0.001	0.001	0.001
(726)	49.8379	1.2150	57438	49239	29547	46725	0.006	0.005	0.003	0.005
(993)	49.8379	1.9159	29588	27087	26348	24111	0.003	0.003	0.003	0.002
(1397)	50.0213	2.2056	13993	12704	12213	11346	0.001	0.001	0.001	0.001
(1422)	50.0379	1.5140	21021	18431	11739	13883	0.002	0.002	0.001	0.001
(1101)	50.0546	2.5888	20352	19268	18228	16404	0.002	0.002	0.002	0.002
(934)	50.0713	1.8598	16418	17008	15207	14479	0.002	0.002	0.002	0.001
(790)	50.1546	1.2150	49327	43593	31239	36322	0.005	0.004	0.003	0.004
(982)	50.2379	1.8318	16455	16359	15362	15034	0.002	0.002	0.002	0.002
(896)	50.4379	1.3178	24970	20615	11329	15120	0.002	0.002	0.001	0.002
(1494)	50.4879	1.5701	10398	9066	6903	7219	0.001	0.001	0.001	0.001
(557)	50.5046	1.1589	37112	32120	24209	34220	0.004	0.003	0.002	0.003
(962)	50.5046	1.8037	19028	18581	17425	16976	0.002	0.002	0.002	0.002
(1742)	50.5379	2.5607	8003	7318	7542	7709	0.001	0.001	0.001	0.001
(603)	50.7213	1.1963	33375	30173	16250	24963	0.003	0.003	0.002	0.003
(1628)	50.7379	2.1682	7209	7302	7125	7081	0.001	0.001	0.001	0.001
(1106)	50.7713	1.7664	11622	11471	10321	10201	0.001	0.001	0.001	0.001

Compound Name	RT 1D (min)	RT 2D (s)	Volume				Percent Response			
			HM_1	HM_2	LM_1	LM_2	HM_1	HM_2	LM_1	LM_2
(548)	50.8879	1.1308	35476	29089	11848	24850	0.004	0.003	0.001	0.003
(760)	50.9213	1.5234	29183	22860	13307	19902	0.003	0.002	0.001	0.002
(1303)	50.9879	1.9720	13816	12228	10683	12867	0.001	0.001	0.001	0.001
(1150)	51.1046	1.2336	23041	19240	13308	18842	0.002	0.002	0.001	0.002
(997)	51.2213	1.7103	14148	13303	11387	11971	0.001	0.001	0.001	0.001
(1346)	51.2379	1.8785	7684	7152	6940	7146	0.001	0.001	0.001	0.001
(1198)	51.3379	1.2243	11809	10798	7404	10850	0.001	0.001	0.001	0.001
(1776)	51.3546	0.8692	9059	7620	7476	7576	0.001	0.001	0.001	0.001
(1511)	51.3879	2.9439	15661	13465	13333	14181	0.002	0.001	0.001	0.001
(380)	51.4379	1.4953	39669	35246	29690	39563	0.004	0.004	0.003	0.004
(630)	51.4379	1.7196	11729	10853	8747	11742	0.001	0.001	0.001	0.001
(1519)	51.4546	2.4673	8310	8028	8800	8826	0.001	0.001	0.001	0.001
(1258)	51.4713	1.9439	11554	10207	12343	11524	0.001	0.001	0.001	0.001
(1396)	51.4713	2.0841	11066	9379	11590	9985	0.001	0.001	0.001	0.001
(656)	51.6213	1.4112	20483	17521	8471	15335	0.002	0.002	0.001	0.002
(842)	51.6213	0.9346	18776	16305	19491	18268	0.002	0.002	0.002	0.002
(1441)	51.6213	2.8411	8904	7981	8494	8795	0.001	0.001	0.001	0.001
(1778)	51.6379	0.7290	7266	6707	6414	7779	0.001	0.001	0.001	0.001
(1141)	51.6546	1.2150	12025	9704	6643	10822	0.001	0.001	0.001	0.001
(984)	51.7213	1.6355	14374	12947	12440	14122	0.001	0.001	0.001	0.001
(1382)	51.7213	2.1402	31864	27751	30444	30702	0.003	0.003	0.003	0.003
(1283)	51.7546	1.1589	12777	9792	8030	11329	0.001	0.001	0.001	0.001
(1453)	51.7546	2.7383	6403	5643	5511	6530	0.001	0.001	0.001	0.001
(1610)	51.7546	2.5514	6281	5695	5981	6421	0.001	0.001	0.001	0.001
(1126)	51.8379	1.4112	17889	16537	8514	12555	0.002	0.002	0.001	0.001
(930)	51.8879	1.6075	14706	14861	11847	14122	0.001	0.001	0.001	0.001
(1270)	51.9546	1.2336	14420	14139	9414	13085	0.001	0.001	0.001	0.001
(1193)	51.9879	2.7009	13449	12337	16972	15827	0.001	0.001	0.002	0.002
(542)	52.1046	2.0280	23171	20084	21412	30039	0.002	0.002	0.002	0.003
(1001)	52.1213	2.2710	11858	11031	11316	13083	0.001	0.001	0.001	0.001
(1250)	52.1213	2.4486	10307	10782	11265	12627	0.001	0.001	0.001	0.001
(422)	52.1546	1.7570	35781	32871	30445	37299	0.004	0.003	0.003	0.004
(1568)	52.1546	1.0187	5405	5667	5609	6466	0.001	0.001	0.001	0.001
(1255)	52.2213	1.2804	19304	17632	11715	17346	0.002	0.002	0.001	0.002
(929)	52.2879	1.5701	15535	16814	11881	14095	0.002	0.002	0.001	0.001
Caffeine (151)	52.3713	3.8224	213386	180931	186469	136361	0.021	0.018	0.019	0.014
(1053)	52.4379	0.9065	20820	19652	19974	19712	0.002	0.002	0.002	0.002
(1129)	52.4379	0.6729	12791	12389	12927	10029	0.001	0.001	0.001	0.001
(904)	52.4379	1.5514	31468	33145	26724	29315	0.003	0.003	0.003	0.003
(1432)	52.4546	2.1589	6842	7483	7739	6593	0.001	0.001	0.001	0.001
(1451)	52.4546	1.9346	9560	9660	12284	9466	0.001	0.001	0.001	0.001

Compound Name	RT 1D (min)	RT 2D (s)	Volume				Percent Response			
			HM_1	HM_2	LM_1	LM_2	HM_1	HM_2	LM_1	LM_2
(981)	52.4713	1.2336	25043	24890	17150	23262	0.002	0.002	0.002	0.002
(1478)	52.4713	2.6075	9604	10137	10667	10697	0.001	0.001	0.001	0.001
(1606)	52.4879	3.2991	7491	7466	7403	7927	0.001	0.001	0.001	0.001
(1676)	52.5213	3.0935	7251	6465	7048	7236	0.001	0.001	0.001	0.001
(1182)	52.6879	2.5794	13947	12166	17512	13694	0.001	0.001	0.002	0.001
(885)	52.7213	1.2617	36419	33789	19802	29621	0.004	0.003	0.002	0.003
(1747)	52.7213	0.5607	5886	5930	5411	5924	0.001	0.001	0.001	0.001
(538)	52.7379	1.8972	25680	23701	20302	30224	0.003	0.002	0.002	0.003
(1113)	52.7546	1.5234	19864	20125	15294	16431	0.002	0.002	0.002	0.002
(1736)	52.7879	0.7196	8315	8488	7220	8424	0.001	0.001	0.001	0.001
(1380)	52.8046	2.3084	17196	12371	12517	11775	0.002	0.001	0.001	0.001
Hept-3-yl isobutyl phthalate (13)	53.0379	2.4953	1458242	1438545	834065	1770640	0.144	0.144	0.085	0.178
(486)	53.0379	3.5607	23494	23573	18537	23536	0.002	0.002	0.002	0.002
(600)	53.0879	1.4953	40403	39498	28416	38580	0.004	0.004	0.003	0.004
(640)	53.1046	0.0561	5498	5558	4653	5959	0.001	0.001	0.000	0.001
(964)	53.1046	0.1682	16673	16331	13638	16744	0.002	0.002	0.001	0.002
(1287)	53.1046	0.6542	15365	15448	13275	15478	0.002	0.002	0.001	0.002
(1011)	53.1213	2.2056	16255	15638	13939	14987	0.002	0.002	0.001	0.002
(1189)	53.1213	0.3925	20012	19796	16644	19298	0.002	0.002	0.002	0.002
(1310)	53.1213	0.9346	20421	20736	17055	20476	0.002	0.002	0.002	0.002
(1268)	53.1213	1.9533	8797	7351	7754	7693	0.001	0.001	0.001	0.001
(1144)	53.1379	1.2617	24762	21621	15809	21378	0.002	0.002	0.002	0.002
(745)	53.2713	1.7757	22786	16854	12999	16584	0.002	0.002	0.001	0.002
(1480)	53.3379	2.1589	6982	6398	7224	6513	0.001	0.001	0.001	0.001
(1308)	53.3713	1.2710	9067	7993	5956	8023	0.001	0.001	0.001	0.001
(1739)	53.4213	3.1308	6288	5638	5883	5764	0.001	0.001	0.001	0.001
(1669)	53.4713	0.9065	9793	8475	9022	9667	0.001	0.001	0.001	0.001
(1506)	53.4879	1.8785	6618	5626	7280	6058	0.001	0.001	0.001	0.001
(639)	53.5046	1.6168	31590	24942	23726	24611	0.003	0.002	0.002	0.002
(1352)	53.5046	1.2710	10995	8836	7683	9351	0.001	0.001	0.001	0.001
(1598)	53.5213	3.4860	14283	13582	12839	13553	0.001	0.001	0.001	0.001
(1755)	53.5379	3.6636	8430	7662	8122	8096	0.001	0.001	0.001	0.001
(1691)	53.6213	0.6168	7835	7082	7298	7009	0.001	0.001	0.001	0.001
(1299)	53.7379	1.1589	15107	12591	9434	11777	0.001	0.001	0.001	0.001
(1385)	53.7879	2.1495	18455	15664	14350	13794	0.002	0.002	0.001	0.001
(1467)	53.7879	2.7477	12012	11434	12168	12335	0.001	0.001	0.001	0.001
(1199)	53.7879	1.4112	20176	22074	17421	17764	0.002	0.002	0.002	0.002
(1780)	53.8713	3.1589	4800	5611	4551	4519	0.000	0.001	0.000	0.000
(1336)	53.9213	1.2150	12001	9896	7633	9779	0.001	0.001	0.001	0.001
(1614)	54.0213	2.1308	15360	13160	13615	12573	0.002	0.001	0.001	0.001
(1088)	54.1713	1.3645	16173	16952	14889	15399	0.002	0.002	0.002	0.002

Compound Name	RT 1D (min)	RT 2D (s)	Volume HM_1	HM_2	LM_1	LM_2	Percent Response HM_1	HM_2	LM_1	LM_2
(1771)	54.2546	2.8598	6303	6994	5712	5822	0.001	0.001	0.001	0.001
(1748)	54.3379	2.5981	7377	6989	5962	6671	0.001	0.001	0.001	0.001
(1438)	54.3379	1.5794	7077	7526	5388	6403	0.001	0.001	0.001	0.001
(1156)	54.3546	1.3551	13297	14554	10415	13459	0.001	0.001	0.001	0.001
(1772)	54.3546	1.9907	8683	7314	8399	6973	0.001	0.001	0.001	0.001
(1602)	54.3713	2.2056	11989	9231	9993	8956	0.001	0.001	0.001	0.001
(1148)	54.5046	1.3178	21195	21497	16635	20590	0.002	0.002	0.002	0.002
(1426)	54.5046	3.0187	14203	12915	16731	14799	0.001	0.001	0.002	0.001
(1666)	54.5546	3.8318	7246	6419	5658	6406	0.001	0.001	0.001	0.001
(1513)	54.5713	3.2150	9894	9052	10216	10338	0.001	0.001	0.001	0.001
(361)	54.6046	2.2710	59079	52657	40087	73848	0.006	0.005	0.004	0.007
(1523)	54.6546	1.7570	9623	8374	8501	8616	0.001	0.001	0.001	0.001
(1437)	54.6879	2.0374	6879	6445	6994	6384	0.001	0.001	0.001	0.001
(1021)	54.7213	1.2897	10177	10169	7907	10127	0.001	0.001	0.001	0.001
(1045)	54.8046	1.4673	15342	14765	11537	14974	0.002	0.001	0.001	0.002
(1643)	54.8046	2.6542	9059	7076	6522	8133	0.001	0.001	0.001	0.001
(987)	54.8213	3.2897	22210	19796	16922	21345	0.002	0.002	0.002	0.002
(1124)	54.8213	1.0561	15388	13589	12078	16579	0.002	0.001	0.001	0.002
(1123)	54.8879	1.2243	19710	19976	16638	20708	0.002	0.002	0.002	0.002
(1625)	54.9879	2.0841	7246	6222	5332	5837	0.001	0.001	0.001	0.001
(1765)	55.0379	3.8411	5205	4424	4983	5362	0.001	0.000	0.001	0.001
(1556)	55.0546	1.9159	8690	6544	7743	7814	0.001	0.001	0.001	0.001
(1644)	55.1546	3.4019	9930	8739	9036	9749	0.001	0.001	0.001	0.001
(1115)	55.2046	1.1402	17578	16967	15328	17069	0.002	0.002	0.002	0.002
(945)	55.2379	2.3364	13196	11329	12293	13117	0.001	0.001	0.001	0.001
(1145)	55.3546	2.6542	16000	14374	13960	16779	0.002	0.001	0.001	0.002
(1548)	55.4046	0.9065	10947	10523	8538	10613	0.001	0.001	0.001	0.001
(1472)	55.4213	1.9626	14375	12074	11361	11470	0.001	0.001	0.001	0.001
(1054)	55.4379	1.1028	15844	15635	14666	14658	0.002	0.002	0.001	0.001
(1557)	55.4546	2.2243	6902	5898	5634	6109	0.001	0.001	0.001	0.001
(1734)	55.4879	2.4486	8550	7924	8002	8285	0.001	0.001	0.001	0.001
(1668)	55.5046	2.6355	5518	5066	4387	4807	0.001	0.001	0.000	0.000
(1729)	55.5546	1.3271	6611	6230	5763	4987	0.001	0.001	0.001	0.001
(1617)	55.5713	1.6636	13901	11062	12587	12101	0.001	0.001	0.001	0.001
(1206)	55.6879	1.0467	9088	9396	8754	7873	0.001	0.001	0.001	0.001
(1646)	55.7046	1.5327	8354	7275	7618	7179	0.001	0.001	0.001	0.001
Dibutyl phtalate (5)	55.9546	1.7944	9903034	7400349	4689719	19576016	0.978	0.741	0.478	1.973
(253)	55.9546	3.7944	46607	40681	35886	71723	0.005	0.004	0.004	0.007
(289)	56.0213	0.0935	65877	60832	47212	93819	0.007	0.006	0.005	0.009
(354)	56.0213	1.0000	108438	99193	81999	143224	0.011	0.010	0.008	0.014
(373)	56.0213	0.4860	40863	37408	30917	55979	0.004	0.004	0.003	0.006

			Volume				Percent Response			
Compound Name	RT 1D (min)	RT 2D (s)	HM_1	HM_2	LM_1	LM_2	HM_1	HM_2	LM_1	LM_2
(523)	56.0213	1.4393	13759	11867	12317	16067	0.001	0.001	0.001	0.002
(1103)	56.2879	1.4673	9832	9544	11008	13638	0.001	0.001	0.001	0.001
(1304)	56.3046	0.4206	6775	5950	6611	7361	0.001	0.001	0.001	0.001
(1374)	56.3379	2.1121	6792	6459	6970	8771	0.001	0.001	0.001	0.001
(1181)	56.4046	1.6636	11451	11324	11170	13476	0.001	0.001	0.001	0.001
(1653)	56.4546	2.5701	4947	4766	5907	5691	0.000	0.000	0.001	0.001
(1579)	56.5546	2.3458	6089	7030	7077	7532	0.001	0.001	0.001	0.001
(976)	56.6379	0.8972	20601	22016	21775	22662	0.002	0.002	0.002	0.002
(1554)	56.6379	3.9065	2533	3785	3487	3652	0.000	0.000	0.000	0.000
(1313)	56.6546	1.6916	8716	9051	9483	10251	0.001	0.001	0.001	0.001
(1375)	56.6546	1.2991	5196	6205	7792	6671	0.001	0.001	0.001	0.001
(1652)	56.6546	2.7850	4078	5299	6097	4726	0.000	0.001	0.001	0.000
(1526)	56.7379	2.4112	9338	11570	11674	10902	0.001	0.001	0.001	0.001
(1711)	56.7379	3.2243	7685	8520	9785	9059	0.001	0.001	0.001	0.001
(1186)	56.7546	1.9720	10573	10199	21332	11932	0.001	0.001	0.002	0.001
(1534)	56.8379	3.8037	5644	6462	6855	6784	0.001	0.001	0.001	0.001
(1659)	56.8546	3.5981	4935	5117	5081	5746	0.000	0.001	0.001	0.001
(1196)	56.8546	1.4766	16858	19186	21354	21621	0.002	0.002	0.002	0.002
(1044)	56.9879	0.8411	17790	18950	17212	19097	0.002	0.002	0.002	0.002
(1510)	57.0046	1.9159	6419	5961	6470	6929	0.001	0.001	0.001	0.001
(1435)	57.0213	1.7290	6201	7541	7203	7756	0.001	0.001	0.001	0.001
(1631)	57.0213	2.1869	6135	6168	5635	6986	0.001	0.001	0.001	0.001
(1151)	57.0879	1.3832	10428	9914	10857	12938	0.001	0.001	0.001	0.001
(1429)	57.1213	1.2243	8131	8481	8859	8797	0.001	0.001	0.001	0.001
(772)	57.2213	1.3832	17137	16183	17841	18911	0.002	0.002	0.002	0.002
(485)	57.2546	2.6168	21333	20255	27988	27582	0.002	0.002	0.003	0.003
(1002)	57.3046	0.8131	24441	23771	22113	21819	0.002	0.002	0.002	0.002
(1413)	57.3213	3.3551	19560	18638	20693	18553	0.002	0.002	0.002	0.002
(1259)	57.3379	3.0935	12814	12283	14648	13313	0.001	0.001	0.001	0.001
(1549)	57.3379	3.9533	2994	3030	3312	3352	0.000	0.000	0.000	0.000
(92)	57.3546	1.3832	84084	82084	79365	163762	0.008	0.008	0.008	0.017
(1443)	57.3546	3.8318	8144	7702	8428	8205	0.001	0.001	0.001	0.001
(1488)	57.3546	3.7290	7456	7380	7471	7762	0.001	0.001	0.001	0.001
(970)	57.5213	0.7850	12235	11703	10979	11936	0.001	0.001	0.001	0.001
(1593)	57.5713	2.5047	5663	6105	5656	6071	0.001	0.001	0.001	0.001
(1246)	57.5713	1.2430	10189	8223	8757	9980	0.001	0.001	0.001	0.001
(1702)	57.6379	3.0000	6954	7215	6318	6825	0.001	0.001	0.001	0.001
(1575)	57.6546	2.2430	6394	6380	6054	6717	0.001	0.001	0.001	0.001
(1635)	57.6713	3.6729	9712	8088	9259	8565	0.001	0.001	0.001	0.001
(596)	57.6879	0.6355	28119	25508	24224	23114	0.003	0.003	0.002	0.002
(1509)	57.7379	2.4766	13103	10756	10130	12066	0.001	0.001	0.001	0.001

			Volume				Percent Response			
**Compound Name**	**RT 1D (min)**	**RT 2D (s)**	**HM_1**	**HM_2**	**LM_1**	**LM_2**	**HM_1**	**HM_2**	**LM_1**	**LM_2**
(1663)	57.7379	2.7383	8150	7379	7381	6587	0.001	0.001	0.001	0.001
(1228)	57.7713	0.4299	16058	14597	14078	14213	0.002	0.001	0.001	0.001
(1479)	57.8379	1.6449	8649	8889	8948	9480	0.001	0.001	0.001	0.001
(1333)	57.8713	3.3832	17809	17258	16081	17806	0.002	0.002	0.002	0.002
(1168)	57.9379	0.3832	14954	13432	11065	13037	0.001	0.001	0.001	0.001
(1128)	58.0213	0.7383	18068	17922	16038	16306	0.002	0.002	0.002	0.002
(1286)	58.0213	1.3271	17806	16336	14124	17189	0.002	0.002	0.001	0.002
(1407)	58.0879	1.1495	8751	7121	7362	8070	0.001	0.001	0.001	0.001
(1608)	58.1213	3.2243	9044	8527	7855	8360	0.001	0.001	0.001	0.001
(1541)	58.1379	0.9813	7548	7197	6417	7204	0.001	0.001	0.001	0.001
(1241)	58.1546	0.4019	11704	11663	10043	10488	0.001	0.001	0.001	0.001
(1147)	58.3713	0.7009	18875	15673	13465	17770	0.002	0.002	0.001	0.002
(1179)	58.4713	0.3832	19647	18116	15311	17702	0.002	0.002	0.002	0.002
(995)	58.5879	0.6916	23996	23575	20815	24339	0.002	0.002	0.002	0.002
(965)	58.8713	0.4019	18545	17124	14807	18405	0.002	0.002	0.002	0.002
(1017)	59.1213	0.3738	14747	14054	12951	12419	0.001	0.001	0.001	0.001
(899)	59.5713	0.3738	18823	19276	16005	16642	0.002	0.002	0.002	0.002
(820)	59.7046	0.3832	23969	20794	22673	22378	0.002	0.002	0.002	0.002
(966)	59.8879	0.5981	12542	10438	9685	11171	0.001	0.001	0.001	0.001
(555)	60.2213	0.3738	79831	74262	66334	72560	0.008	0.007	0.007	0.007
(865)	60.4046	0.5794	55400	49476	49265	52426	0.005	0.005	0.005	0.005
(540)	60.7046	0.3738	44489	45054	42399	45078	0.004	0.005	0.004	0.005
(710)	60.7213	0.5607	42109	39918	39086	38514	0.004	0.004	0.004	0.004
(593)	60.8546	0.3738	31266	26343	27103	27492	0.003	0.003	0.003	0.003
(794)	60.9879	0.5607	30425	28213	28731	28958	0.003	0.003	0.003	0.003
(506)	61.0879	0.3738	39415	41112	35544	40499	0.004	0.004	0.004	0.004
(474)	61.3546	0.3738	35733	37039	29315	35520	0.004	0.004	0.003	0.004
(613)	61.4213	0.5514	37808	36730	34200	33744	0.004	0.004	0.003	0.003
(578)	61.6379	0.5607	28056	27757	26224	26995	0.003	0.003	0.003	0.003
(443)	61.7713	0.3738	52938	51654	44473	52799	0.005	0.005	0.005	0.005
(516)	61.8713	0.5607	21666	23381	23901	25887	0.002	0.002	0.002	0.003
(522)	62.0213	0.5607	41160	40218	39298	41248	0.004	0.004	0.004	0.004
(390)	62.3546	0.3738	77057	75779	67660	73098	0.008	0.008	0.007	0.007
(489)	62.3713	0.5514	84593	81733	79219	81466	0.008	0.008	0.008	0.008
(534)	62.6213	0.5607	28753	26882	27457	27039	0.003	0.003	0.003	0.003
(369)	62.8213	0.3738	90783	93840	77942	87604	0.009	0.009	0.008	0.009
(477)	62.9046	0.5607	46597	46397	41981	43900	0.005	0.005	0.004	0.004
(384)	62.9379	0.3738	53504	47537	41406	44645	0.005	0.005	0.004	0.004
(345)	63.2379	0.3738	51225	50639	44722	52344	0.005	0.005	0.005	0.005
(340)	63.3379	0.3738	51004	47336	42529	47320	0.005	0.005	0.004	0.005
(457)	63.3713	0.5607	64673	65578	58353	61990	0.006	0.007	0.006	0.006

			Volume				Percent Response			
**Compound Name**	**RT 1D (min)**	**RT 2D (s)**	**HM_1**	**HM_2**	**LM_1**	**LM_2**	**HM_1**	**HM_2**	**LM_1**	**LM_2**
(458)	63.6213	0.5607	45028	42398	43249	41673	0.004	0.004	0.004	0.004
(323)	63.7713	0.3738	156981	164975	134764	152382	0.015	0.017	0.014	0.015
(393)	63.9213	0.5607	74989	73533	76707	71444	0.007	0.007	0.008	0.007
(401)	64.2046	0.5607	47607	50213	48321	48838	0.005	0.005	0.005	0.005
(297)	64.2546	0.3738	131145	141182	118545	133386	0.013	0.014	0.012	0.013
(388)	64.3879	0.5607	64610	61806	62825	61506	0.006	0.006	0.006	0.006
(415)	64.5713	0.5607	38886	40194	39300	39094	0.004	0.004	0.004	0.004
(305)	64.7046	0.3738	165735	180590	144283	156499	0.016	0.018	0.015	0.016
(537)	64.7379	0.8037	26770	27822	28995	26342	0.003	0.003	0.003	0.003
(332)	64.8213	0.5607	110677	110372	108496	112516	0.011	0.011	0.011	0.011
(705)	65.1546	0.2056	22215	21855	24154	24533	0.002	0.002	0.002	0.002
(275)	65.1879	0.3738	150453	163587	136476	144155	0.015	0.016	0.014	0.015
(365)	65.2713	0.5607	111104	116449	106028	112307	0.011	0.012	0.011	0.011
(269)	65.4379	0.3738	79944	88845	77335	86367	0.008	0.009	0.008	0.009
(271)	65.6713	0.3738	83434	86497	73213	80898	0.008	0.009	0.007	0.008
(347)	65.7713	0.5607	137386	135839	127036	140036	0.014	0.014	0.013	0.014
(804)	65.7713	0.1589	20822	20869	23221	20589	0.002	0.002	0.002	0.002
(307)	65.9046	0.3738	72497	74770	51727	70865	0.007	0.007	0.005	0.007

Notes:

1) Listed are the 1450 peak-regions found by GC Image
2) HM: high elevation, monsoon pu-erh, LM: low elevation, monsoon pu-erh
3) Compounds with asterisks (107) were found by GC Image without the assistance of Ion Analytics
4) Compounds without asterisks (200) were confirmed by GC Image after analysis by Ion Analytics (307 total compounds detected)
5) Percent response calculated from the peak volume divided by the sum of all peak volumes

Table S2. Compounds identified by Ion Analytics

Compound	Target (T) / Untargeted (UT)	High Elevation		Low Elevation	
		RT	Relative Response*	RT	Relative Response*
Dimethyl sulfide	T	4.36	2.0852	4.30	3.1503
Acetic acid	T	4.96	6.4513	4.56	1.5136
Hexane	T	4.77	2.7920	4.70	2.6224
2-Methylfuran	T	4.83	1.5972	4.83	0.9846
(3E)-2-Methyl-1,3-pentadiene	T	5.17	0.4288	5.17	0.1315
3-Methylbutanal	T	5.43	0.0978	5.36	0.0381
(2E)-Butenal	T	5.43	0.0195	5.36	0.0622
2-Methylbutanal	T	5.49	1.1981	5.49	1.2191
Cyclohexane	UT	5.44	2.0038	5.56	5.0913
Penten-3-ol	T	5.70	4.2661	5.76	2.9236
319	UT	5.83	0.2382	5.83	1.1451
Pentanal	T	5.96	0.5525	6.03	0.4923
2-Ethylfuran	T	6.03	2.2178	6.09	1.4354
(3E)-Penten-2-one	T	6.77	1.0268	6.90	0.7779
2-Methyl-3-pentanone	T	7.03	0.0864	7.10	0.0199
Dimethyl disulfide	T	6.97	0.0315	7.17	0.0157
Pyridine	T	7.04	0.2614	7.17	0.1666
2-Methyl-2-butenal	UT	6.90	0.2657	6.97	0.1084
Pentanol	T	7.37	3.6966	7.51	1.6222
(2Z)-Pentenol	T	7.44	3.2772	7.57	2.0383
Toluene	T	7.50	10.8205	7.63	11.6565
311	T	7.50	0.8669	7.64	0.5672
Octene	T	8.03	0.0805	8.16	0.0258
2-Hexanone	T	7.97	0.1711	8.11	0.2025
2-Propylfuran	UT	8.03	0.1892	8.17	0.0721
Octane	UT	8.23	1.5460	8.36	1.0899
4-Methyl-3-penten-2-one	UT	8.24	1.1730	8.44	0.9497
Hexanal	T	8.24	0.6515	8.44	0.5220
2-Ethyl-5-methylfuran	UT	8.30	0.4258	8.50	0.0620
(3Z,5Z)-Octadiene	UT	8.63	0.0439	8.83	0.0424
(4E)-Octene	UT	8.70	0.1839	8.90	0.1301
2,4-Octadiene	UT	8.90	0.0701	9.10	0.0404
Butyl acetate	T	8.64	0.0674	8.84	0.3186
1-Ethyl-1H-pyrrole	T	8.77	1.8014	8.97	0.6792
Isovaleric acid	T	9.20	0.1667	9.33	0.0906
1,3-Octadiene	UT	9.03	0.1119	9.23	0.1500
2-Methyl-2-pentenal	T	9.31	0.0370	9.51	0.0143
Furfural	UT	9.38	0.0463	—	—
18	T	9.44	0.0203	—	—
2-Methylbutanoic acid	T	9.53	0.0706	9.73	0.0564
2,5,5-Trimethyl-1-hexen-3-yne	T	9.77	0.2764	9.97	0.2599
(2E)-Hexenal	UT	9.98	0.0547	—	—
2-Furanmethanol	T	10.06	0.0598	—	—
2-(2-Propenyl)furan	UT	10.10	0.0449	10.37	0.0156
(3Z)-Hexenol	T	10.05	3.2796	10.31	0.0286
10	T	10.30	0.0641	10.50	0.0514
Ethylbenzene	T	10.44	0.2014	10.64	0.3217
(2E)-Hexenol	T	10.45	1.0070	—	—
(2Z)-Hexenol	T	10.58	0.0471	10.25	0.0161
Hexanol	T	10.51	0.3811	10.71	0.0725
p-Xylene	T	10.70	0.5339	10.90	0.7051
1,3-trans,5-cis-octatriene	UT	10.97	0.2116	11.17	0.2235
n-Butyl ether	UT	11.10	0.0379	11.30	0.2329
2-Heptanone	T	11.38	0.6060	11.64	0.4306
2-Butylfuran	UT	11.50	0.4444	11.70	0.2201

Compound	Target (T) / Untargeted (UT)	High Elevation		Low Elevation	
		RT	Relative Response*	RT	Relative Response*
Styrene	T	11.51	2.7068	11.77	3.8074
Methoxy-phenyl-oxime	UT	11.69	8.1978	11.75	0.5309
Butyl-2-propenoate	T	11.57	0.6394	11.84	4.3467
Nonane	T	11.70	0.2603	12.37	0.6379
2-Heptanol	UT	11.71	0.4728	—	—
Heptanal	T	11.84	0.1199	12.04	0.1134
483	UT	12.11	0.0578	12.37	0.0432
Butyl propanoate	T	12.11	0.0747	12.31	1.1969
2,5-Dimethylpyrazine	UT	12.31	0.0971	—	—
Butyrolactone	T	12.55	0.1409	12.68	0.0412
Amyl acetate	T	12.31	0.0276	12.57	0.0198
2-Acetylfuran	UT	12.32	0.1684	12.59	0.0816
2(5H)-Furanone	T	12.62	0.0914	—	—
484	UT	12.64	0.0638	12.84	0.0190
485	UT	13.31	0.3394	13.51	0.2091
α-Pinene	T	13.37	0.2885	13.57	0.2410
486	UT	13.58	0.4476	13.84	0.2952
316	T	13.99	0.1005	14.25	0.0064
Camphene	T	14.04	0.0655	14.30	0.0334
6-Methyl-2-heptanone	T	14.18	0.0426	14.38	0.0524
Propylbenzene	UT	14.18	0.0858	14.44	0.1059
(2E)-Heptenal	UT	14.25	0.1583	14.45	0.1998
Benzaldehyde	T	14.52	3.7549	14.79	1.9456
p-Ethyltoluene	T	14.58	0.1801	14.84	0.2109
3,5,5-Trimethyl-2-hexene	UT	14.98	3.8800	15.25	3.8993
Heptanol	T	14.79	0.0493	—	—
487	UT	15.11	0.0412	15.37	0.0388
Dimethyl trisulfide	T	15.05	0.0797	15.25	0.0723
Hexanoic acid	T	15.01	1.8020	15.14	0.3366
Octen-3-one	T	15.25	0.6164	15.45	1.0975
Octen-3-ol	T	15.25	13.2913	15.45	15.4288
Phenol	T	15.30	0.8458	15.49	0.1682
β-Pinene	T	15.37	0.0776	15.57	0.0868
o-Ethyltoluene	T	15.44	0.0287	15.64	0.0267
2,3-Octanedione	T	15.45	1.1556	15.65	1.4751
(3E)-Hexenoic acid	UT	15.75	0.0728	—	—
101	T	15.66	0.0907	—	—
2,6-Dimethyl-2,6-octadiene	UT	15.64	0.2641	15.90	0.1098
6-Methyl-5-Hepten-2-one	T	15.65	0.6711	15.85	0.6170
Myrcene	T	15.84	1.0951	16.04	0.5794
2-Octanone	T	15.78	0.3535	16.05	0.3439
2-Pentylfuran	T	15.84	8.5018	16.11	5.5629
Dehydroxy-trans-linalool oxide	T	15.98	0.2803	16.18	0.1129
Butyl butanoate	T	15.98	0.2263	16.24	1.5925
1,2,4-Trimethylbenzene	T	16.04	0.2623	16.24	0.2793
(2E,4Z)-Heptadienal	T	16.79	1.2942	16.33	0.4595
Decane	UT	16.17	0.1086	16.44	0.0889
cis-2,6-Dimethyl-2,6-octadiene	UT	16.30	0.3976	16.50	0.1667
α-Phellandrene	T	16.57	0.2528	16.77	0.1933
trans-2-(2-Pentenyl)furan	UT	16.31	0.3036	16.58	0.1900
Octanal	T	16.38	0.0596	16.58	0.0788
2-(2-Ethoxyethoxy)-ethanol	UT	16.52	0.3603	16.66	0.2292
Dehydroxy-cis-linalool oxide	T	16.71	0.2875	16.91	0.1047
(3Z)-Hexenyl acetate	T	16.51	0.1655	—	—
(2E,4E)-Heptadienal	T	16.79	1.2356	16.99	1.1019
δ-3-Carene	T	16.84	0.0364	17.11	0.0448
488	UT	16.99	0.8505	17.19	0.2508

Compound	Target (T) / Untargeted (UT)	High Elevation		Low Elevation	
		RT	Relative Response*	RT	Relative Response*
α-Terpinene	T	17.17	0.2892	17.37	0.1692
102	T	17.40	0.0358	17.53	0.0261
1,2,3-Trimethylbenzene	T	17.45	0.1350	17.65	0.0592
para-Cymene	T	17.51	1.3794	17.71	1.6442
2-Ethyl-1-hexanol	T	17.59	1.0960	17.78	0.8661
Limonene	T	17.71	3.8650	17.91	3.1051
(Z)-β-Ocimene	UT	18.04	0.5992	18.24	0.2681
Eucalyptol	T	17.91	0.0128	18.11	0.0239
Benzyl alcohol	T	17.93	1.3626	18.14	0.1492
2,2,6-Trimethylcyclohexanone	T	18.05	1.1822	18.25	1.9650
Indane	T	18.11	0.0719	18.38	0.0715
(3E)-Octen-2-one	UT	18.12	0.6201	18.32	0.4330
5-Ethyl-2(5H)-furanone	T	18.15	0.1070	18.29	0.0533
(E)-β-Ocimene	T	18.57	2.1278	18.77	0.6000
Indene	T	18.58	0.0419	18.78	0.0258
Benzeneacetaldehyde	T	18.46	1.5868	18.66	1.5554
Isobutyl angelate	UT	18.71	0.0665	18.91	0.1054
1-Ethyl-1H-pyrrole-2-carboxaldehyde	T	18.79	1.4226	18.93	0.5986
(2E)-Octenal	UT	19.06	0.1182	19.26	0.1091
γ-Terpinene	T	19.17	1.0409	19.37	1.0396
17	T	19.25	0.6094	19.45	0.7917
(2E)-Octenol	T	19.45	1.7861	19.66	2.9604
Acetophenone	T	19.59	0.3528	19.79	0.3663
Octanol	T	19.59	0.7350	19.79	0.7200
Heptanoic acid	T	19.61	0.2764	—	—
123	T	19.66	1.4007	19.86	1.4363
para-Cresol	T	19.82	0.1128	20.02	0.1241
cis-Linalool oxide (furanoid)	T	19.85	2.7055	20.05	0.0778
489	UT	19.97	0.2001	20.17	0.1863
490	UT	19.91	0.0469	20.11	0.0948
491	UT	20.04	0.3653	20.24	0.3581
492	UT	19.97	0.1894	20.64	0.1351
493	UT	20.31	0.1118	20.44	0.2046
2-Nonanone	T	20.72	0.1432	20.85	0.2443
trans-Linalool oxide (furanoid)	T	20.65	8.1600	20.78	0.0576
494	UT	20.50	0.2837	20.64	0.1831
Terpinolene	T	20.64	0.6027	20.84	0.3725
para-Cymenene	T	20.71	0.1686	20.85	0.0874
2-Methoxyphenol	T	20.66	0.1263	—	—
(3E,5E)-Octadien-2-one	T	20.79	0.3273	20.99	0.2311
Nonanal	T	21.32	0.5859	21.45	0.3576
Undecane	UT	21.04	0.9798	21.24	0.3887
Linalool	T	21.12	13.0092	21.25	1.0975
495	UT	21.37	0.3598	21.44	0.1671
Hotrienol	T	21.32	5.1178	21.52	1.1974
105	T	21.66	0.1893	21.86	0.2314
6-Methyl-3,5-heptadiene-2-one	UT	21.39	0.3113	21.53	0.2243
Maltol	T	21.58	0.0443	—	—
cis-Thujone	UT	21.59	1.0000	21.72	1.0000
496	UT	21.77	0.8377	21.90	0.3849
497	UT	21.90	1.2425	22.04	0.4483
114	T	21.86	0.0518	—	—
Phenylethyl alcohol	T	21.87	4.1185	22.00	0.2573
498	UT	21.91	0.8848	22.04	0.3788
α-Cyclocitral	T	22.12	0.0362	22.32	0.0614
1,3,8-p-Menthatriene	UT	21.98	0.0317	22.38	0.0565
499	UT	22.17	0.2901	22.24	0.0850

Compound	Target (T) / Untargeted (UT)	High Elevation		Low Elevation	
		RT	Relative Response*	RT	Relative Response*
2-Ethyl-m-xylene	UT	22.25	0.0811	22.38	0.0400
trans-Thujone	UT	22.12	0.1066	22.25	0.1752
500	UT	22.44	0.8784	22.57	0.2697
allo-Ocimene	T	22.51	0.0509	22.71	0.0169
501	UT	22.65	0.1554	22.78	0.0420
502	UT	22.70	0.4021	22.84	0.0899
Pentylcyclohexane	UT	22.84	0.0724	23.04	0.0214
503	UT	22.84	0.3397	22.97	0.1156
3-Nonen-2-one	UT	23.06	0.0949	23.19	0.0349
1-Ethyl-2,5-pyrrolidinedione	T	22.88	0.1220	23.01	0.0377
504	UT	22.97	0.4666	23.10	0.1043
Benzyl nitrile	T	23.14	2.3935	23.28	0.1192
(R,S)-5-Ethyl-6-methyl-(3E)-hepten-2-one	T	23.39	0.0909	23.59	0.0343
Menthone	T	23.92	0.1146	24.05	0.1247
2-Ethylhexyl acetate	T	23.51	0.2497	23.65	0.2041
505	UT	23.64	0.5648	23.70	0.1018
506	UT	23.70	0.5727	23.84	0.1819
507	UT	23.77	0.4532	23.90	0.1137
93	T	23.99	0.0307	24.12	0.0611
86	T	23.79	0.0339	23.92	0.0320
508	UT	23.97	0.4938	24.10	0.1145
509	UT	24.17	1.1400	24.30	0.2431
Octanoic acid	T	24.34	0.4761	24.41	0.0880
Menthofuran	UT	24.38	0.0739	24.51	0.0838
510	UT	24.50	1.1067	24.64	0.2803
Nonanol	T	24.52	0.1567	24.65	0.0679
cis-Linalool oxide (pyranoid)	T	24.59	0.6798	24.73	0.0846
Ethyl benzoate	T	24.65	2.2252	24.79	1.8609
Menthol	T	24.79	0.0350	24.92	0.0264
trans-Linalool oxide (pyranoid)	T	24.79	3.3896	24.93	0.3980
Terpinen-4-ol	T	25.05	0.1369	25.18	0.1177
2,4-Dimethylbenzaldehyde	T	24.93	0.0457	24.99	0.0311
511	UT	25.24	0.2561	—	—
(3Z)-Hexenyl butyrate	T	25.31	0.5908	—	—
Naphthalene	T	25.46	1.5372	25.59	0.8007
2,6-Dimethyl-3,7-octadiene-2,6-diol	T	25.47	0.4280	25.54	0.1359
Dodecene	UT	25.51	0.2004	25.64	0.1125
(2E)-Hexenyl butyrate	UT	25.65	0.2955	—	—
Hexyl butyrate	UT	25.51	0.0448	—	—
2-Decanone	T	25.59	0.1954	25.72	0.2368
α-Terpineol	T	25.65	0.7392	25.79	0.3167
Methyl salicylate	T	25.86	16.7247	25.99	2.5181
Dodecane	UT	25.91	3.6915	26.04	1.1799
Methyl chavicol	UT	25.98	0.0824	26.12	0.0847
Safranal	T	26.12	0.2605	26.26	0.2679
513	UT	26.57	0.3849	26.70	0.1227
Decanal	T	26.25	0.1239	26.32	0.0444
512	UT	26.51	0.0560	—	NA
129	T	26.52	0.0633	26.65	0.0480
131	T	26.99	0.6501	27.06	0.3871
2-Phenoxyethanol	T	27.01	0.1049	27.07	0.0394
514	UT	27.04	0.0706	—	—
β-Cyclocitral	T	27.12	0.4802	27.26	0.5672
Nerol	T	27.32	0.0416	—	—
(3Z)-Hexenyl valerate	UT	27.51	0.2300	—	—
(3Z)-Hexenyl isovalerate	T	27.65	0.0198	—	—
Bornylene	T	27.52	0.0643	27.65	0.0338

Compound	Target (T) / Untargeted (UT)	High Elevation		Low Elevation	
		RT	Relative Response*	RT	Relative Response*
515	UT	27.78	0.5410	27.91	0.2642
516	UT	27.97	0.4282	28.11	0.1364
132	T	28.00	0.7552	28.13	0.5258
518	UT	28.17	0.1284	28.24	0.0280
Carvone	T	28.26	0.0119	28.33	0.0290
Geraniol	T	28.52	1.4990	28.59	0.0723
Linalool acetate	T	28.58	0.0358	28.71	0.0318
Phenylethyl acetate	T	28.72	0.0808	—	—
519	UT	28.91	0.1720	28.97	0.0555
β-Homocyclocitral	T	28.92	0.0270	28.99	0.0164
Nonanoic acid	T	29.00	0.3943	29.00	0.1058
Pentyl hexanoate	UT	30.05	0.0336	—	—
520	UT	29.91	0.1693	30.04	0.0728
(E)-Anethole	UT	30.19	0.0111	30.25	0.0285
521	UT	30.24	1.3423	30.31	0.4050
2-Undecanone	UT	30.32	0.1309	30.45	0.0880
Indole	T	30.56	2.2271	30.69	3.7285
Tridecane	UT	30.57	1.9677	30.64	0.7187
2-Methylnaphthalene	T	30.66	0.3123	30.72	0.1132
1-Nitro-2-phenyl ethane	UT	30.81	0.1114	—	—
Theaspirane A	T	30.91	0.1542	30.98	0.0634
522	UT	31.05	0.1273	31.12	0.0501
523	UT	31.17	0.4005	31.24	0.0855
1-Methylnaphthalene	T	31.39	0.1880	31.52	0.0971
524	UT	31.37	0.4260	31.64	0.1514
Theaspirane B	T	31.65	0.1971	31.71	0.0877
525	UT	32.71	0.5204	32.84	0.1662
Heptylcyclohexane	UT	32.64	1.1154	32.71	0.4385
170	T	32.68	0.0580	32.75	0.0375
Triacetin	UT	32.80	0.1582	32.87	0.0409
526	UT	32.97	0.2614	33.04	0.0782
26	T	33.09	1.5689	33.16	1.4158
Decanoic acid	T	33.40	0.1680	33.47	0.0913
γ-Nonalactone	T	33.61	0.0521	—	—
527	UT	33.77	1.4570	33.84	0.4936
528	UT	33.98	0.2369	34.04	0.1099
Butyl benzoate	UT	34.06	0.5459	34.12	0.4584
3-Hydroxy-2,4,4-trimethylpentyl-2-methylpropanoate	T	34.06	0.1122	34.13	0.0600
(3Z)-Hexenyl hexenoate	T	34.25	1.0678	—	—
529	UT	34.44	0.5411	34.44	0.2278
β-Cubebene	UT	34.38	0.0593	34.44	0.0239
Hexyl hexanoate	UT	34.45	0.0708	—	—
(2E)-Hexenyl caproate	UT	34.58	0.1576	—	—
530	UT	34.64	0.6903	34.71	0.2801
531	UT	34.65	0.0175	34.71	0.0259
β-Bourbonene	UT	34.78	0.1545	34.84	0.0676
Tetradecane	UT	35.04	2.2115	35.11	0.7495
(Z)-Jasmone	T	35.26	0.1465	—	—
Vanillin	T	35.22	0.1723	35.28	0.0377
532	UT	35.31	0.9584	35.37	0.3244
Dodecanal	T	35.45	0.0788	35.52	0.0257
534	UT	36.04	0.2769	36.11	0.1012
533	UT	35.84	0.3226	35.91	0.0893
535	UT	36.31	0.3376	36.38	0.0613
α-Ionone	T	36.46	0.1717	36.52	0.1024
536	UT	36.64	0.2834	36.71	0.0806

Compound	Target (T) / Untargeted (UT)	High Elevation RT	High Elevation Relative Response*	Low Elevation RT	Low Elevation Relative Response*
Nonylcyclopentane	UT	37.17	1.0775	37.24	0.5080
Geranylacetone	T	37.39	0.0584	37.45	0.0649
537	UT	37.71	0.9771	37.71	0.4554
Dodecanol	T	37.97	0.2047	38.04	0.1089
Octylbenzene	UT	37.98	0.0363	38.05	0.0276
538	UT	38.71	0.1346	38.71	0.1060
539	UT	38.91	0.4531	38.97	0.2702
(E)-β-Ionone	T	38.92	0.7622	38.99	0.5114
5,6-Epoxy-β-Ionone	T	38.93	0.4072	39.00	0.3417
Pentadecane	UT	39.24	0.6897	39.24	0.2839
(E,E)-α-Farnesene	T	39.71	0.0452	—	—
2,4-Di-tert-butylphenol	T	39.86	0.0290	—	—
Butylated hydroxytoluene	T	39.98	0.2349	40.05	0.1616
178	T	40.53	0.4065	40.53	0.2498
δ-Cadinene	T	40.51	0.0402	—	—
cis-Calamenene	T	40.58	0.0592	—	—
Dihydroactinidiolide	T	40.95	0.3877	41.02	0.3114
540	UT	41.24	0.4070	41.31	0.1446
Nonylcylohexane	UT	41.44	0.7154	41.44	0.3210
α-Calacorene	T	41.38	0.0298	41.45	0.0149
Dodecanoic acid	T	42.00	0.0322	—	—
541	UT	42.11	0.4323	42.11	0.2154
542	UT	42.86	0.1212	42.92	0.0858
Caryophyllene oxide	T	43.12	0.0216	—	—
Hexadecane	UT	43.31	0.5144	43.31	0.2129
(2,4,4-trimethyl-3-(2-methylpropanoyloxy)pentyl) 2-methylpropanoate	T	43.45	0.8169	43.45	0.5154
tau-Muurolol	T	45.32	0.0396	—	—
543	UT	45.44	0.2291	45.44	0.1141
cis-Methyl dihydrojasmonate	T	45.53	0.4615	45.53	0.2354
α-Cadinol	T	45.79	0.0303	—	—
Tetradecanol	T	46.71	0.0294	46.77	0.0319
3,4-Diethyl-1,1'-biphenyl	T	46.59	0.0480	46.65	0.0109
Bancroftinone	UT	47.65	0.0307	—	—
Undecylcyclohexane	UT	49.24	0.0742	49.31	0.0142
Caffeine	UT	52.42	0.6229	52.49	0.4457
Ethyl acetate	T	—	—	4.84	0.0247
Isoamyl alcohol	T	—	—	7.23	0.0832
(2E)-Pentenal	T	—	—	7.24	0.1036
m-Ethyltoluene	T	—	—	15.11	0.0254
118	T	—	—	24.26	0.1061

* Relative response normalized to cis-thujone internal standard response
